# Multi-criteria group decision making based on Archimedean power partitioned Muirhead mean operators of q-rung orthopair fuzzy numbers

**DOI:** 10.1371/journal.pone.0221759

**Published:** 2019-09-05

**Authors:** Yuchu Qin, Qunfen Qi, Paul J. Scott, Xiangqian Jiang

**Affiliations:** School of Computing and Engineering, University of Huddersfield, Huddersfield, West Yorkshire, England, United Kingdom; Shandong University of Science and Technology, CHINA

## Abstract

Two critical tasks in multi-criteria group decision making (MCGDM) are to describe criterion values and to aggregate the described information to generate a ranking of alternatives. A flexible and superior tool for the first task is q-rung orthopair fuzzy number (qROFN) and an effective tool for the second task is aggregation operator. So far, nearly thirty different aggregation operators of qROFNs have been presented. Each operator has its distinctive characteristics and can work well for specific purpose. However, there is not yet an operator which can provide desirable generality and flexibility in aggregating criterion values, dealing with the heterogeneous interrelationships among criteria, and reducing the influence of extreme criterion values. To provide such an aggregation operator, Muirhead mean operator, power average operator, partitioned average operator, and Archimedean T-norm and T-conorm operations are concurrently introduced into q-rung orthopair fuzzy sets, and an Archimedean power partitioned Muirhead mean operator of qROFNs and its weighted form are presented and a MCGDM method based on the weighted operator is proposed in this paper. The generalised expressions of the two operators are firstly defined. Their properties are explored and proved and their specific expressions are constructed. On the basis of the specific expressions, a method for solving the MCGDM problems based on qROFNs is then designed. Finally, the feasibility and effectiveness of the method is demonstrated via a numerical example, a set of experiments, and qualitative and quantitative comparisons.

## 1. Introduction

Multi-criteria group decision making (MCGDM) refers to the process of finding optimal alternatives in complex scenarios via synthetically evaluating the values of multiple criteria of all alternatives provided by a group of domain experts [1]. In this process, there are two critical tasks. One critical task is to describe the values of different criteria accurately and effectively. For such description, there are many different kinds of available tools, where fuzzy set is a well-known kind [2–12]. To date, nearly thirty different types of fuzzy sets have been presented [13]. Yager’s generalised orthopair fuzzy set [14], commonly known as q-rung orthopair fuzzy set (qROFS), is one of the most important and popular types among them.

A qROFS consists of an element and a q-rung orthopair membership grade (commonly known as q-rung orthopair fuzzy number (qROFN), which is used to quantify the degrees of membership and non-membership of the element to the qROFS. In a qROFS, both the degrees of membership and non-membership and the sum of the *q*-th (*q* = 1, 2, 3, …) power of the degree of membership and the *q*-th power of the degree of non-membership are restricted to [0, 1]. In other words, the rung *q* in a qROFS is adjustable under the premise of satisfying this condition. Because of this characteristic, qROFS can be regarded as the generalisation of Zadeh’s fuzzy set (FS) [15], Atanassov’s intuitionistic fuzzy set (IFS) [16], and Yager’s Pythagorean fuzzy set (PFS) [17]. This is because qROFS will reduce to FS when *q* = 1 and the sum of the degrees of membership and non-membership is equal to 1, will reduce to IFS when *q* = 1, and will reduce to PFS when *q* = 2. In addition, the expressiveness of a qROFS will continue to increase as *q* increases, which provides enough freedom for the description of fuzzy information. Due to such advantage, qROFSs have received extensive attention in the field of MCGDM during the past few years. Various research topics regarding qROFSs for MCGDM, such as correlation and correlation coefficient of qROFSs [18], distance measures of qROFSs [19], similarity measures of qROFSs [20], application of qROFSs in practical MCGDM problems [21, 22], operational rules of qROFNs [23–25], and aggregation operators of qROFNs [26−38], are gaining importance and popularity within academia.

The other critical task in MCGDM is to fuse the described criterion information to generate a ranking of all alternatives. For such fusion, aggregation operator is regarded as an effective tool. So far, over twenty different aggregation operators of qROFNs have been presented. They are the weighted averaging (WA) operator and the weighted geometric (WG) operator presented by Liu and Wang [26], the weighted Bonferroni mean (WBM) operator and the weighted geometric Bonferroni mean (WGBM) operator presented by Liu and Liu [27], the weighted Archimedean Bonferroni mean (WABM) operators presented by Liu and Wang [28], the weighted partitioned Bonferroni mean (WPBM) operator and the weighted partitioned geometric Bonferroni mean (WPGBM) operator presented by Yang and Pang [29], the weighted Heronian mean (WHM) operator and the weighted geometric Heronian mean (WGHM) operator presented by Wei et al. [30], the WHM* operator (This operator is different from the WHM operator presented by Wei et al. [30], though they have the same names) and the weighted partitioned Heronian mean (WPHM) operator presented by Liu et al. [31], the weighted Maclaurin symmetric mean (WMSM) operator and the weighted geometric Maclaurin symmetric mean (WGMSM) operator presented by Wei et al. [32], the weighted power Maclaurin symmetric mean (WPMSM) operator presented by Liu et al. [33], the weighted power partitioned Maclaurin symmetric mean (WPPMSM) operator presented by Bai et al. [34], the weighted Muirhead mean (WMM) operator and the weighted geometric Muirhead mean (WGMM) operator presented by Wang et al. [35], the weighted extended Bonferroni mean (WEBM) operator presented by Liu et al. [36], the weighted exponential (WE) operator presented by Peng et al. [37], and the weighted point (WP) operators presented by Xing et al. [38]. Each operator has its own characteristics and can work well for its specific purpose. But there is not yet an operator that has the following three characteristics at the same time: (1) Provide satisfying generality and flexibility in the aggregation of qROFNs; (2) Deal with the situation in which the criteria are divided into several parts and there are interrelationships among different criteria in each part whereas the criteria in different parts are independent of each other; (3) Reduce the negative effect of the unduly high or unduly low criterion values on the aggregation results.

In practical MCGDM problems, aggregation of criterion values is a complex process, in which the preferences of decision makers may change frequently. An ideal aggregation operator should be general and flexible enough to adapt to such change. Moreover, there are usually complex relationships among the different criteria considered in the problems. It is also of importance for an aggregation operator to capture the complex interrelationships of different criteria to generate more reasonable aggregation results [28]. Further, the values of criteria are generally assessed by domain experts. It is often difficult to ensure the absolute objectivity, which means that a few biased experts will give biased assessment values [33]. To obtain reasonable aggregation results, it is of necessity to reduce the negative influence of biased criterion values in the aggregation. Based on these considerations, the motivations of the present paper are explained as follows:

1. To develop an aggregation operator of qROFNs which can capture the complex interrelationships among criteria, the Muirhead mean (MM) operator [39] and partitioned average operator are introduced. The MM operator, which is a generalisation of the generalised arithmetic average operator, Bonferroni mean (BM) operator [40, 41], Maclaurin symmetric mean (MSM) operator [42, 43], and generalised geometric average operator, is an all-in-one aggregation operator for capturing the interrelationships of criteria. It is applicable in the cases where all criteria are independent of each other, where there are interrelationships between any two criteria, and where there are interrelationships between any multiple (three or more) criteria [44–49]. The partitioned average operator is an aggregation operator that has the capability of aggregating the parameters in different partitions using the same aggregation operator and aggregating the aggregation results of different partitions using the arithmetic average operator [50–55].
2. To enable the aggregation operator to reduce the negative effect of extreme criterion values on the aggregation results, the power average (PA) operator [56] is combined into the partitioned MM operator. The PA operator is an aggregation operator that can assign weights to the aggregated parameters by calculating the support degrees between these parameters, which makes it capable of reducing the negative influence of unreasonable parameter values on the aggregation results [57–62].
3. To improve the generality and flexibility of the combined aggregation operator, the operational rules of qROFNs based on the Archimedean T-norm and T-conorm (ATT) are leveraged to perform the operations in the operator. The ATT are operations for generalising the logical conjunction and disjunction to fuzzy logic. They are important tools that can generate versatile and flexible operational rules for fuzzy numbers and the aggregation operators based on them are rather general and flexible for aggregating fuzzy information [28, 63–68].

As can be summarised from the motivations above, this paper aims to present a set of Archimedean power partitioned MM operators of qROFNs and propose a MCGDM method based on them. This aim is achieved through combining the MM operator, the partitioned average operator, the PA operator, and the ATT. As a result, the presented aggregation operators combine all of their characteristics.

The remainder of the paper is organised as follows. A brief introduction of some basic concepts is provided Section 2. Sections 3 explains the details of the presented Archimedean power partitioned MM operators. The specific process of the proposed MCGDM method is described in Section 4. Section 5 demonstrates and evaluates the presented operators and proposed method via example, experiments, and comparisons. Section 6 ends the paper with a conclusion.

## 2. Preliminaries

In this section, some prerequisites in qROFS theory, operational rules of qROFNs based on ATT, PA operator, and MM operator are briefly introduced to facilitate the understanding of the present paper.

### 2.1. qROFS theory

qROFS [14] is the generalisation of FS [15], IFS [16], and PFS [17]. Its formal definition is as follow:

**Definition 1** [14]. A qROFS *S* in a finite universe of discourse *X* is *S* = {<*x*, *μ*_*S*_(*x*), *ν*_*S*_(*x*)> | *x*∈*X*}, where *μ*_*S*_: *X* → [0, 1] is the degree of membership of *x*∈*X* to *S*, and *ν*_*S*_: *X* → [0, 1] is the degree of non-membership of *x*∈*X* to *S*, such that 0 ≤ (*μ*_*S*_(*x*))^*q*^ + (*ν*_*S*_(*x*))^*q*^ ≤ 1 (*q* = 1, 2, 3,…). The degree of hesitancy of *x*∈*X* to *S* is *π*_*S*_(*x*) = (1 − (*μ*_*S*_(*x*))^*q*^ − (*ν*_*S*_(*x*))^*q*^)^1/*q*^.

For convenience, a pair <*μ*_*S*_(*x*), *ν*_*S*_(*x*)> is called a qROFN, which is commonly simplified as *Q* = <*μ*, *ν*>. To compare two qROFNs, their scores and accuracies are required, which can be calculated according to the following definitions:

**Definition 2** [26]. Let *Q* = <*μ*, *ν*> be a qROFN. Then its score is *S*(*Q*) = *μ*^*q*^ − *ν*^*q*^. Obviously, −1 ≤ *S*(*Q*) ≤ 1.

**Definition 3** [26]. Let *Q* = <*μ*, *ν*> be a qROFN. Then its accuracy is *A*(*Q*) = *μ*^*q*^ + *ν*^*q*^. Obviously, 0 ≤ *A*(*Q*) ≤ 1.

Using *S*(*Q*) and *A*(*Q*), two qROFNs can be compared via the following definition:

**Definition 4** [26]. Let *Q*_1_ = <*μ*_1_, *ν*_1_> and *Q*_2_ = <*μ*_2_, *ν*_2_> be any two qROFNs, *S*(*Q*_1_) and *S*(*Q*_2_) be respectively the scores of *Q*_1_ and *Q*_2_, and *A*(*Q*_1_) and *A*(*Q*_2_) be respectively the accuracies of *Q*_1_ and *Q*_2_. Then: (1) If *S*(*Q*_1_) > *S*(*Q*_2_), then *Q*_1_ > *Q*_2_; (2) If *S*(*Q*_1_) = *S*(*Q*_2_) and *A*(*Q*_1_) > *A*(*Q*_2_), then *Q*_1_ > *Q*_2_; (3) If *S*(*Q*_1_) = *S*(*Q*_2_) and *A*(*Q*_1_) = *A*(*Q*_2_), then *Q*_1_ = *Q*_2_.

To calculate the distance between two qROFNs, a distance measure of qROFNs is required. The following definition provides the Minkowski-type distance measure of qROFNs:

**Definition 5** [19]. Let *Q*_1_ = <*μ*_1_, *ν*_1_> and *Q*_2_ = <*μ*_2_, *ν*_2_> be any two qROFNs. Then the Minkowski-type distance between *Q*_1_ and *Q*_2_ is *D*(*Q*_1_, *Q*_2_) = (0.5|*μ*_1_−*μ*_2_|^*b*^ + 0.5|*ν*_1_−*ν*_2_|^*b*^)^1/*b*^ (*b* = 1, 2, …).

If *b* = 1, the Minkowski-type distance between *Q*_1_ and *Q*_2_ will reduce to the Hamming distance between *Q*_1_ and *Q*_2_: *D*(*Q*_1_, *Q*_2_) = 0.5(|*μ*_1_−*μ*_2_| + |*ν*_1_−*ν*_2_|); If *b* = 2, the Minkowski-type distance between *Q*_1_ and *Q*_2_ will reduce to the Euclidean distance between *Q*_1_ and *Q*_2_: (0.5|*μ*_1_−*μ*_2_|^2^ + 0.5|*ν*_1_−*ν*_2_|^2^)^1/2^; If *b* = ∞, the Minkowski-type distance between *Q*_1_ and *Q*_2_ will reduce to the Chebyshev distance between *Q*_1_ and *Q*_2_: *D*(*Q*_1_, *Q*_2_) = max{|*μ*_1_−*μ*_2_|, |*ν*_1_−*ν*_2_|}.

### 2.2. Operational rules of qROFNs based on ATT

Based on ATT, a set of general and versatile operational rules of qROFNs were established by Liu and Wang [28]. The formal definition of the rules is as follow:

**Definition 6** [28]. Let *Q* = <*μ*, *ν*>, *Q*_1_ = <*μ*_1_, *ν*_1_>, and *Q*_2_ = <*μ*_2_, *ν*_2_> be any three qROFNs, and *a* and *b* be any two real numbers and *a*, *b* > 0. Then the sum, product, multiplication, and power operations of qROFNs based on the Archimedean T-norm *T*(*x*, *y*) = *f*^−1^(*f*(*x*) + *f*(*y*)) and its T-conorm T*(*x*, *y*) = *g*^−1^(*g*(*x*) + *g*(*y*)) can be respectively defined as follows:

$$Q_{1}⊕Q_{2}=\langle T^{*}(\mu_{1},\mu_{2}),\;T(\nu_{1},\nu_{2})\rangle =\langle g^{-1}\left(g(\mu_{1})+g(\mu_{2})\right),f^{-1}\left(f(\nu_{1})+f(\nu_{2})\right)\rangle$$ (1)

$$Q_{1}⊗Q_{2}=\langle T(\mu_{1},\mu_{2}),\;T^{*}(\nu_{1},\nu_{2})\rangle =\langle f^{-1}\left(f(\mu_{1})+f(\mu_{2})\right),\;g^{-1}\left(g(\nu_{1})+g(\nu_{2})\right)\rangle$$ (2)

$$aQ=\langle g^{-1}\left(ag(\mu )\right),\;f^{-1}\left(af(\nu )\right)\rangle$$ (3)

$$Q^{b}=\langle f^{-1}\left(bf(\mu )\right),\;g^{-1}\left(bg(\nu )\right)\rangle$$ (4)

### 2.3. PA operator

The PA operator, introduced by Yager [56], can assign weights to the aggregated arguments via computing the degrees of support between these arguments. This makes it possible to reduce the negative influence of the unduly high or unduly low argument values on the aggregation results. The formal definition of the PA operator is as follow:

**Definition 7** [56]. Let (*a*_1_, *a*_2_, …, *a*_*n*_) be a collection of crisp numbers, *Sup*(*a*_*i*_, *a*_*j*_) = 1 − *D*(*a*_*i*_, *a*_*j*_) (*i*, *j* = 1, 2, …, *n* and *j* ≠ *i*; *D*(*a*_*i*_, *a*_*j*_) is the distance between *a*_*i*_ and *a*_*j*_) be the degree of support for *a*_*i*_ from *a*_*j*_ which has the following properties: (1) 0 ≤ *Sup*(*a*_*i*_, *a*_*j*_) ≤ 1; (2) *Sup*(*a*_*i*_, *a*_*j*_) = *Sup*(*a*_*j*_, *a*_*i*_); (3) *Sup*(*a*_*i*_, *a*_*j*_) ≥ *Sup*(*a*_*r*_, *a*_*s*_) if |*a*_*i*_−*a*_*j*_| ≤ |*a*_*r*_−*a*_*s*_|, and

$$T(a_{i})=\sum_{j=1,j\neq i}^{n}Sup(a_{i},\;a_{j})$$ (5)

Then the aggregation function

$$PA(a_{1},a_{2},\ldots ,a_{n})=\frac{\sum_{i=1}^{n}\left(\left(1+T(a_{i})\right)a_{i}\right)}{\sum_{i=1}^{n}\left(1+T(a_{i})\right)}$$ (6)

is called the PA operator.

### 2.4. Partitioned average operator

The partitioned average operator can aggregate the arguments in different partitions using the same aggregation operator and aggregate the aggregation results of different partitions using the arithmetic average operator [51]. Its formal definition is as follow:

**Definition 8** [51]. Let (*a*_1_, *a*_2_, …, *a*_*n*_) be a collection of crisp numbers, ***S*** = {*a*_1_, *a*_2_, …, *a*_*n*_} be a set of *a*_1_, *a*_2_, …, *a*_*n*_, ***S***_*k*_ = {*a*_1_, *a*_2_, …, *a*_|***S****k*|_} (*k* = 1, 2, …, *N*) be *N* partitions of ***S*** (i.e. ***S***_1_ ∪ ***S***_2_ ∪ … ∪ ***S***_*N*_ = ***S*** and ***S***_1_ ∩ ***S***_2_ ∩ … ∩ ***S***_*N*_ = Ø), and *AO* be a specific aggregation operator. Then the aggregation function

$$PtA(a_{1},a_{2},\ldots ,a_{n})=\frac{1}{N}\sum_{k=1}^{N}\left(\overset{\left|S_{k}\right|}{\underset{i_{k}=1}{AO}}\left(a_{i_{k}}\right)\right)$$ (7)

is called the partitioned average operator.

### 2.5. MM operator

The MM operator was firstly introduced to aggregate crisp numbers by Muirhead [39]. It has prominent characteristics in capturing the interrelationships among multiple aggregated arguments and providing a general form of a number of other aggregation operators. The formal definition of the MM operator is as follow:

**Definition 9** [39]. Let (*a*_1_, *a*_2_, …, *a*_*n*_) be a collection of crisp numbers, Δ = (*δ*_1_, *δ*_2_, …, *δ*_*n*_) (where *δ*_1_, *δ*_2_, …, *δ*_*n*_ ≥ 0 but not at the same time *δ*_1_ = *δ*_2_ = … = *δ*_*n*_ = 0) be a collection of *n* real numbers, *p*(*i*) be a permutation of (1, 2,…, *n*), and ***P***_*n*_ be the set of all permutations of (1, 2,…, *n*). Then the aggregation function

$$MM^{\Delta }(a_{1},a_{2},\ldots ,a_{n})={\left(\frac{1}{n!}\sum_{p\in P_{n}}\prod_{i=1}^{n}a_{p(i)}^{\delta_{i}}\right)}^{\frac{1}{\sum_{i=1}^{n}\delta_{i}}}$$ (8)

is called the MM operator.

In this operator, whether the interrelationships are considered depends on the values of *δ*_*i*_ (*i* = 1, 2, …, *n*): (1) If *δ*_1_ > 0 and *δ*_2_ = *δ*_3_ = … = *δ*_*n*_ = 0, then the interrelationships are not considered; (2) If *δ*_1_, *δ*_2_ > 0 and *δ*_3_ = *δ*_4_ = … = *δ*_*n*_ = 0, then the interrelationships between two crisp numbers are considered; (3) If *δ*_1_, *δ*_2_, …, *δ*_*k*_ > 0 (*k* = 3, 4, …, *n*) and *δ*_*k*+1_ = *δ*_*k*+2_ = … = *δ*_*n*_ = 0, then the interrelationships among *k* crisp numbers are considered.

## 3. Aggregation operators

In this section, a q-rung orthopair fuzzy Archimedean power partitioned MM (qROFAPPMM) operator and a q-rung orthopair fuzzy weighted Archimedean power partitioned MM (qROFWAPPMM) operator are presented. The properties of the two operators are explored and their specific cases are discussed.

### 3.1. qROFAPPMM operator

A qROFAPPMM operator is a power partitioned MM operator of qROFNs, in which the sum, product, multiplication, and power operations are performed using the operational rules of qROFNs based on ATT. Its formal definition is as follow:

**Definition 10**. Let *Q*_1_, *Q*_2_, …, *Q*_*n*_ (*Q*_*i*_ = <*μ*_*i*_, *ν*_*i*_>, *i* = 1, 2, …, *n*) be *n* qROFNs (*q* = 1, 2, 3, …), (*Q*_1_, *Q*_2_, …, *Q*_*n*_) be a collection of *Q*_1_, *Q*_2_, …, *Q*_*n*_, ***S*** = {*Q*_1_, *Q*_2_, …, *Q*_*n*_} be an ordered set of *Q*_1_, *Q*_2_, …, *Q*_*n*_, ***S***_*k*_ = {*Q*_1_, *Q*_2_, …, *Q*_|***S****k*|_} (*k* = 1, 2, …, *N*) be *N* partitions of ***S*** (i.e. ***S***_1_ ∪ ***S***_2_ ∪ … ∪ ***S***_*N*_ = ***S*** and ***S***_1_ ∩ ***S***_2_ ∩ … ∩ ***S***_*N*_ = Ø), *δ*_1_, *δ*_2_, …, *δ*_|***S****k*|_ (*k* = 1, 2, …, *N* and *δ*_1_, *δ*_2_, …, *δ*_|***S****k*|_ ≥ 0 but not at the same time *δ*_1_ = *δ*_2_ = … = *δ*_|***S****k*|_ = 0) be |***S***_*k*_| real numbers that respectively correspond to *Q*_1_, *Q*_2_, …, *Q*_|***S****k*|_, **Δ**_*k*_ = (*δ*_1_, *δ*_2_, …, *δ*_|***S****k*|_) be a collection of *δ*_1_, *δ*_2_, …, *δ*_|***S****k*|_, **Δ** = (**Δ**_1_, **Δ**_2_, …, **Δ**_*N*_) be a collection of **Δ**_1_, **Δ**_2_, …, **Δ**_*N*_, *p*(*i*_*k*_) be a permutation of (1, 2,…, |***S***_*k*_|), ***P***_|***S****k*|_ be a set of all permutations of (1, 2,…, |***S***_*k*_|), *Q*_*i*_⊕*Q*_*j*_ and *Q*_*i*_⊗*Q*_*j*_ (*i*, *j* = 1, 2, …, *n*) be respectively the sum and product operations of *Q*_*i*_ and *Q*_*j*_ based on ATT, *aQ*_*r*_ and *Q*_*s*_^*b*^ (*r*, *s* = 1, 2, …, *n*; *a*, *b* > 0) be respectively the multiplication operation of *Q*_*r*_ and the power operation of *Q*_*s*_ based on ATT, *Sup*(*Q*_*r*_, *Q*_*s*_) = 1 − *D*(*Q*_*r*_, *Q*_*s*_) (*r*, *s* = 1, 2, …, *n* and *s* ≠ *r*; *D*(*Q*_*r*_, *Q*_*s*_) is the distance between *Q*_*r*_ and *Q*_*s*_) be the degree of support for *Q*_*r*_ from *Q*_*s*_ which satisfy 0 ≤ *Sup*(*Q*_*r*_, *Q*_*s*_) ≤ 1, *Sup*(*Q*_*r*_, *Q*_*s*_) = *Sup*(*Q*_*s*_, *Q*_*r*_), and *Sup*(*Q*_*r*_, *Q*_*s*_) ≥ *Sup*(*Q*_*u*_, *Q*_*v*_) if |*Q*_*r*_−*Q*_*s*_| ≤ |*Q*_*u*_−*Q*_*v*_|, and

$$T(Q_{r})=\sum_{s=1,s\neq r}^{n}Sup(Q_{r},\;Q_{s})$$ (9)

Then the aggregation function

$$qROFAPPMM^{\Delta }(Q_{1},Q_{2},\ldots ,Q_{n})=\frac{1}{N}\left(\overset{N}{\underset{k=1}{⊕}}{\left(\frac{1}{\left|S_{k}\right|!}\underset{p\in P_{\left|S_{k}\right|}}{⊕}\overset{\left|S_{k}\right|}{\underset{i_{k}=1}{⊗}}{\left(\frac{n\left(1+T(Q_{p(i_{k})})\right)}{\sum_{j=1}^{n}\left(1+T(Q_{j})\right)}Q_{p(i_{k})}\right)}^{\delta_{i_{k}}}\right)}^{\frac{1}{\sum_{i_{k}=1}^{\left|S_{k}\right|}\delta_{i_{k}}}}\right)$$ (10)

is called the qROFAPPMM operator. In this operator, the values of *δ*_*ik*_ (*i*_*k*_ = 1, 2, …, |***S***_*k*_|) are used to capture the interrelationships among the aggregated qROFNs in each of the *N* partitions ***S***: (1) If *δ*_1_ > 0 and *δ*_2_ = *δ*_3_ = … = *δ*_|***S****k*|_ = 0, then the qROFNs in the *k*-th partition ***S***_*k*_ are independent of each other; (2) If *δ*_1_, *δ*_2_ > 0 and *δ*_3_ = *δ*_4_ = … = *δ*_|***S****k*|_ = 0, then the interrelationships between two qROFNs in ***S***_*k*_ are considered; (3) If *δ*_1_, *δ*_2_, …, *δ*_*r*_ > 0 (*r* = 3, 4, …, *n*) and *δ*_*r*+1_ = *δ*_*r*+2_ = … = *δ*_|***S****k*|_ = 0, then the interrelationships among *r* qROFNs in ***S***_*k*_ are considered.

According to Eq (1)‒(4) and (10), the following theorem is obtained:

**Theorem 1**. Let *Q*_1_, *Q*_2_, …, *Q*_*n*_ (*Q*_*i*_ = <*μ*_*i*_, *ν*_*i*_>, *i* = 1, 2, …, *n*) be *n* qROFNs (*q* = 1, 2, 3, …) and (*Q*_1_, *Q*_2_, …, *Q*_*n*_) be a collection of *Q*_1_, *Q*_2_, …, *Q*_*n*_. Then

$$qROFAPPMM^{\Delta }(Q_{1},Q_{2},\ldots ,Q_{n})=\langle \mu ,\;\nu \rangle$$ (11)

and it is still a qROFN, where

$$\mu =g^{-1}\left(\frac{1}{N}\sum_{k=1}^{N}g\left(f^{-1}\left(\frac{1}{\sum_{i_{k}=1}^{\left|S_{k}\right|}\delta_{i_{k}}}f\left(g^{-1}\left(\frac{1}{\left|S_{k}\right|!}\sum_{p\in P_{\left|S_{k}\right|}}g\left(f^{-1}\left(\sum_{i_{k}=1}^{\left|S_{k}\right|}\left(\delta_{i_{k}}f\left(g^{-1}\left(\left(n\xi_{p(i_{k})})g(\mu_{p(i_{k})}\right)\right)\right)\right)\right)\right)\right)\right)\right)\right)\right)$$ (12)

$$\nu =f^{-1}\left(\frac{1}{N}\sum_{k=1}^{N}f\left(g^{-1}\left(\frac{1}{\sum_{i_{k}=1}^{\left|S_{k}\right|}\delta_{i_{k}}}g\left(f^{-1}\left(\frac{1}{\left|S_{k}\right|!}\sum_{p\in P_{\left|S_{k}\right|}}f\left(g^{-1}\left(\sum_{i_{k}=1}^{\left|S_{k}\right|}\left(\delta_{i_{k}}g\left(f^{-1}\left(\left(n\xi_{p(i_{k})})f(\nu_{p(i_{k})}\right)\right)\right)\right)\right)\right)\right)\right)\right)\right)\right)$$ (13)

and *ξ*_*p*(*ik*)_ is a PA factor which can be calculated via the following Equation:

$$\xi_{i}=\left(1+\sum_{r=1,r\neq x}^{n}\left(1-D(Q_{i},Q_{r})\right)\right)/\sum_{j=1}^{n}\left(1+\sum_{s=1,s\neq j}^{n}\left(1-D(Q_{j},Q_{s})\right)\right)$$ (14)

For the details regarding the proof of this theorem, please refer to Appendix A in S1 File. The following two theorems respectively state the idempotency and boundedness of the qROFAPPMM operator:

**Theorem 2**. Let *Q*_1_, *Q*_2_, …, *Q*_*n*_ (*Q*_*i*_ = <*μ*_*i*_, *ν*_*i*_>, *i* = 1, 2, …, *n*) be *n* qROFNs (*q* = 1, 2, 3, …) and (*Q*_1_, *Q*_2_, …, *Q*_*n*_) be a collection of *Q*_1_, *Q*_2_, …, *Q*_*n*_. If *Q*_*i*_ = *Q* = <*μ*_*Q*_, *ν*_*Q*_> for all *i* = 1, 2, …, *n*, then *qROFAPPMM*^**Δ**^(*Q*_1_, *Q*_2_, …, *Q*_*n*_) = *Q* = <*μ*_*Q*_, *ν*_*Q*_>.

**Theorem 3**. Let *Q*_1_, *Q*_2_, …, *Q*_*n*_ (*Q*_*i*_ = <*μ*_*i*_, *ν*_*i*_>, *i* = 1, 2, …, *n*) be *n* qROFNs (*q* = 1, 2, 3, …), (*Q*_1_, *Q*_2_, …, *Q*_*n*_) be a collection of *Q*_1_, *Q*_2_, …, *Q*_*n*_, *Q*_UB_ = <max{*μ*_*i*_}, min{*ν*_*i*_}>, and *Q*_LB_ = <min{*μ*_*i*_}, max{*ν*_*i*_}>. Then *Q*_LB_ ≤ *qROFAPPMM*^Δ^(*Q*_1_, *Q*_2_, …, *Q*_*n*_) ≤ *Q*_UB_.

For the details regarding the proofs of these two theorems, please refer to Appendixes B and C in S1 File, respectively.

From Eq (11), it is not difficult to see that the qROFAPPMM operator has the capabilities to capture the interrelationships of the aggregated qROFNs and to reduce the negative influence of unreasonable aggregated qROFNs, because the operator is constructed via combining the MM, partitioned average, and PA operators. In addition, it can also be seen that the qROFAPPMM operator has desirable generality, since different specific qROFAPPMM operators will be obtained if different if specific functions are assigned to *f*. For example, if the additive generators of Algebraic, Einstein, Hamacher, and Frank T-norms and T-conorms are assigned to *f*, then four specific operators can be respectively constructed as follows:

1. If *f*(*t*) = −In*t*^*q*^, then *g*(*t*) = −In(1−*t*^*q*^), *f*^−1^(*t*) = (e^−*t*^)^1/*q*^, and *g*^−1^(*t*) = (1−e^−*t*^)^1/*q*^. A q-rung orthopair fuzzy Archimedean Algebraic power partitioned MM (qROFAAPPMM) operator is constructed according to Eq (11):
  $$\begin{array}{l} qROFAAPPMM^{\Delta }(Q_{1},Q_{2},\ldots ,Q_{n})=\langle {\left(1-{\left(\prod_{k=1}^{N}\left(1-{\left(1-{\left(\prod_{p\in P_{\left|S_{k}\right|}}\left(1-\prod_{i_{k}=1}^{\left|S_{k}\right|}{\left(1-{\left(1-\mu_{p(i_{k})}^{q}\right)}^{n\xi_{p(i_{k})}}\right)}^{\delta_{i_{k}}}\right)\right)}^{\frac{1}{\left|S_{k}\right|!}}\right)}^{\frac{1}{\sum_{i_{k}=1}^{\left|S_{k}\right|}\delta_{i_{k}}}}\right)\right)}^{\frac{1}{N}}\right)}^{1/q},\\ \;{\left({\left(\prod_{k=1}^{N}\left(1-{\left(1-{\left(\prod_{p\in P_{\left|S_{k}\right|}}\left(1-\prod_{i_{k}=1}^{\left|S_{k}\right|}{\left(1-\nu_{p(i_{k})}^{qn\xi_{p(i_{k})}}\right)}^{\delta_{i_{k}}}\right)\right)}^{\frac{1}{\left|S_{k}\right|!}}\right)}^{\frac{1}{\sum_{i_{k}=1}^{\left|S_{k}\right|}\delta_{i_{k}}}}\right)\right)}^{\frac{1}{N}}\right)}^{1/q}\rangle \end{array}$$ (15)
  where *ξ*_*p*(*ik*)_ is a PA factor which can be calculated via Eq (14).
2. If *f*(*t*) = In[(2−*t*^*q*^)/*t*^*q*^], then *g*(*t*) = In[(1+*t*^*q*^)/(1−*t*^*q*^)], *f*^−1^(*t*) = [2/(e^*t*^+1)]^1/*q*^, and *g*^−1^(*t*) = [(e^*t*^−1)/(e^*t*^+1)]^1/*q*^. A q-rung orthopair fuzzy Archimedean Einstein power partitioned MM (qROFAEPPMM) operator is constructed according to Eq (11):
  $$\begin{array}{l} qROFAEPPMM^{\Delta }(Q_{1},Q_{2},\ldots ,Q_{n})=\langle {\left(\frac{{\left(\prod_{k=1}^{N}\left(\frac{{\left(\mu^{\prime }+3\right)}^{1/\sum_{i_{k}=1}^{\left|S_{k}\right|}\delta_{i_{k}}}+3{\left(\mu^{\prime }-1\right)}^{1/\sum_{i_{k}=1}^{\left|S_{k}\right|}\delta_{i_{k}}}}{{\left(\mu^{\prime }+3\right)}^{1/\sum_{i_{k}=1}^{\left|S_{k}\right|}\delta_{i_{k}}}-{\left(\mu^{\prime }-1\right)}^{1/\sum_{i_{k}=1}^{\left|S_{k}\right|}\delta_{i_{k}}}}\right)\right)}^{\frac{1}{N}}-1}{{\left(\prod_{k=1}^{N}\left(\frac{{\left(\mu^{\prime }+3\right)}^{1/\sum_{i_{k}=1}^{\left|S_{k}\right|}\delta_{i_{k}}}+3{\left(\mu^{\prime }-1\right)}^{1/\sum_{i_{k}=1}^{\left|S_{k}\right|}\delta_{i_{k}}}}{{\left(\mu^{\prime }+3\right)}^{1/\sum_{i_{k}=1}^{\left|S_{k}\right|}\delta_{i_{k}}}-{\left(\mu^{\prime }-1\right)}^{1/\sum_{i_{k}=1}^{\left|S_{k}\right|}\delta_{i_{k}}}}\right)\right)}^{\frac{1}{N}}+1}\right)}^{1/q},\\ \;{\left(2/\left({\left(\prod_{k=1}^{N}\left(\frac{{\left(\nu^{\prime }+3\right)}^{1/\sum_{i_{k}=1}^{\left|S_{k}\right|}\delta_{i_{k}}}+3{\left(\nu^{\prime }-1\right)}^{1/\sum_{i_{k}=1}^{\left|S_{k}\right|}\delta_{i_{k}}}}{{\left(\nu^{\prime }+3\right)}^{1/\sum_{i_{k}=1}^{\left|S_{k}\right|}\delta_{i_{k}}}-{\left(\nu^{\prime }-1\right)}^{1/\sum_{i_{k}=1}^{\left|S_{k}\right|}\delta_{i_{k}}}}\right)\right)}^{\frac{1}{N}}+1\right)\right)}^{1/q}\rangle \end{array}$$ (16)
  where
  $$\mu^{\prime }={\left(\prod_{p\in P_{\left|S_{k}\right|}}\left(\frac{\prod_{i_{k}=1}^{\left|S_{k}\right|}{\left(\frac{{\left(1+\mu_{p(i_{k})}^{q}\right)}^{n\xi_{p(i_{k})}}+3{\left(1-\mu_{p(i_{k})}^{q}\right)}^{n\xi_{p(i_{k})}}}{{\left(1+\mu_{p(i_{k})}^{q}\right)}^{n\xi_{p(i_{k})}}-{\left(1-\mu_{p(i_{k})}^{q}\right)}^{n\xi_{p(i_{k})}}}\right)}^{\delta_{i_{k}}}+3}{\prod_{i_{k}=1}^{\left|S_{k}\right|}{\left(\frac{{\left(1+\mu_{p(i_{k})}^{q}\right)}^{n\xi_{p(i_{k})}}+3{\left(1-\mu_{p(i_{k})}^{q}\right)}^{n\xi_{p(i_{k})}}}{{\left(1+\mu_{p(i_{k})}^{q}\right)}^{n\xi_{p(i_{k})}}-{\left(1-\mu_{p(i_{k})}^{q}\right)}^{n\xi_{p(i_{k})}}}\right)}^{\delta_{i_{k}}}-1}\right)\right)}^{\frac{1}{\left|S_{k}\right|!}}$$ (17)
  $$\nu^{\prime }={\left(\prod_{p\in P_{\left|S_{k}\right|}}\left(\frac{\prod_{i_{k}=1}^{\left|S_{k}\right|}{\left(\frac{{\left(2-\nu_{p(i_{k})}^{q}\right)}^{n\xi_{p(i_{k})}}+3\nu_{p(i_{k})}^{qn\xi_{p(i_{k})}}}{{\left(2-\nu_{p(i_{k})}^{q}\right)}^{n\xi_{p(i_{k})}}-\nu_{p(i_{k})}^{qn\xi_{p(i_{k})}}}\right)}^{\delta_{i_{k}}}+3}{\prod_{i_{k}=1}^{\left|S_{k}\right|}{\left(\frac{{\left(2-\nu_{p(i_{k})}^{q}\right)}^{n\xi_{p(i_{k})}}+3\nu_{p(i_{k})}^{qn\xi_{p(i_{k})}}}{{\left(2-\nu_{p(i_{k})}^{q}\right)}^{n\xi_{p(i_{k})}}-\nu_{p(i_{k})}^{qn\xi_{p(i_{k})}}}\right)}^{\delta_{i_{k}}}-1}\right)\right)}^{\frac{1}{\left|S_{k}\right|!}}$$ (18)
  and *ξ*_*p*(*ik*)_ is a PA factor which can be calculated via Eq (14).
3. If *f*(*t*) = In{[*λ*+(1−*λ*)*t*^*q*^]/*t*^*q*^} (*λ* > 0), then *g*(*t*) = In{[*λ*+(1−*λ*)(1−*t*^*q*^)]/(1−*t*^*q*^)}, *f*^−1^(*t*) = [*λ*/(e^*t*^+*λ*−1)]^1/*q*^, and *g*^−1^(*t*) = [(e^*t*^−1)/ (e^*t*^+*λ*−1)]^1/*q*^. A q-rung orthopair fuzzy Archimedean Hamacher power partitioned MM (qROFAHPPMM) operator is constructed according to Eq (11):
  $$\begin{array}{l} qROFAHPPMM^{\Delta }(Q_{1},Q_{2},\ldots ,Q_{n})=\langle {\left(\frac{{\left(\prod_{k=1}^{N}\left(\frac{{\left(\mu^{\prime }+\lambda^{2}-1\right)}^{1/\sum_{i_{k}=1}^{\left|S_{k}\right|}\delta_{i_{k}}}+(\lambda^{2}-1){\left(\mu^{\prime }-1\right)}^{1/\sum_{i_{k}=1}^{\left|S_{k}\right|}\delta_{i_{k}}}}{{\left(\mu^{\prime }+\lambda^{2}-1\right)}^{1/\sum_{i_{k}=1}^{\left|S_{k}\right|}\delta_{i_{k}}}-{\left(\mu^{\prime }-1\right)}^{1/\sum_{i_{k}=1}^{\left|S_{k}\right|}\delta_{i_{k}}}}\right)\right)}^{\frac{1}{N}}-1}{{\left(\prod_{k=1}^{N}\left(\frac{{\left(\mu^{\prime }+\lambda^{2}-1\right)}^{1/\sum_{i_{k}=1}^{\left|S_{k}\right|}\delta_{i_{k}}}+(\lambda^{2}-1){\left(\mu^{\prime }-1\right)}^{1/\sum_{i_{k}=1}^{\left|S_{k}\right|}\delta_{i_{k}}}}{{\left(\mu^{\prime }+\lambda^{2}-1\right)}^{1/\sum_{i_{k}=1}^{\left|S_{k}\right|}\delta_{i_{k}}}-{\left(\mu^{\prime }-1\right)}^{1/\sum_{i_{k}=1}^{\left|S_{k}\right|}\delta_{i_{k}}}}\right)\right)}^{\frac{1}{N}}+\lambda -1}\right)}^{1/q},\\ \;{\left(\lambda /\left({\left(\prod_{k=1}^{N}\left(\frac{{\left(\nu^{\prime }+\lambda^{2}-1\right)}^{1/\sum_{i_{k}=1}^{\left|S_{k}\right|}\delta_{i_{k}}}+(\lambda^{2}-1){\left(\nu^{\prime }-1\right)}^{1/\sum_{i_{k}=1}^{\left|S_{k}\right|}\delta_{i_{k}}}}{{\left(\nu^{\prime }+\lambda^{2}-1\right)}^{1/\sum_{i_{k}=1}^{\left|S_{k}\right|}\delta_{i_{k}}}-{\left(\nu^{\prime }-1\right)}^{1/\sum_{i_{k}=1}^{\left|S_{k}\right|}\delta_{i_{k}}}}\right)\right)}^{\frac{1}{N}}+\lambda -1\right)\right)}^{1/q}\rangle \end{array}$$ (19)
  where
  $$\mu^{\prime }={\left(\prod_{p\in P_{\left|S_{k}\right|}}\left(\frac{\prod_{i_{k}=1}^{\left|S_{k}\right|}{\left(\frac{{\left(\lambda +(1-\lambda )(1-\mu_{p(i_{k})}^{q})\right)}^{n\xi_{p(i_{k})}}+(\lambda^{2}-1){\left(1-\mu_{p(i_{k})}^{q}\right)}^{n\xi_{p(i_{k})}}}{{\left(\lambda +(1-\lambda )(1-\mu_{p(i_{k})}^{q})\right)}^{n\xi_{p(i_{k})}}-{\left(1-\mu_{p(i_{k})}^{q}\right)}^{n\xi_{p(i_{k})}}}\right)}^{\delta_{i_{k}}}+\lambda^{2}-1}{\prod_{i_{k}=1}^{\left|S_{k}\right|}{\left(\frac{{\left(\lambda +(1-\lambda )(1-\mu_{p(i_{k})}^{q})\right)}^{n\xi_{p(i_{k})}}+(\lambda^{2}-1){\left(1-\mu_{p(i_{k})}^{q}\right)}^{n\xi_{p(i_{k})}}}{{\left(\lambda +(1-\lambda )(1-\mu_{p(i_{k})}^{q})\right)}^{n\xi_{p(i_{k})}}-{\left(1-\mu_{p(i_{k})}^{q}\right)}^{n\xi_{p(i_{k})}}}\right)}^{\delta_{i_{k}}}-1}\right)\right)}^{\frac{1}{\left|S_{k}\right|!}}$$ (20)
  $$\nu^{\prime }={\left(\prod_{p\in P_{\left|S_{k}\right|}}\left(\frac{\prod_{i_{k}=1}^{\left|S_{k}\right|}{\left(\frac{{\left(\lambda +(1-\lambda )\nu_{p(i_{k})}^{q}\right)}^{n\xi_{p(i_{k})}}+(\lambda^{2}-1)\nu_{p(i_{k})}^{qn\xi_{p(i_{k})}}}{{\left(\lambda +(1-\lambda )\nu_{p(i_{k})}^{q}\right)}^{n\xi_{p(i_{k})}}-\nu_{p(i_{k})}^{qn\xi_{p(i_{k})}}}\right)}^{\delta_{i_{k}}}+\lambda^{2}-1}{\prod_{i_{k}=1}^{\left|S_{k}\right|}{\left(\frac{{\left(\lambda +(1-\lambda )\nu_{p(i_{k})}^{q}\right)}^{n\xi_{p(i_{k})}}+(\lambda^{2}-1)\nu_{p(i_{k})}^{qn\xi_{p(i_{k})}}}{{\left(\lambda +(1-\lambda )\nu_{p(i_{k})}^{q}\right)}^{n\xi_{p(i_{k})}}-\nu_{p(i_{k})}^{qn\xi_{p(i_{k})}}}\right)}^{\delta_{i_{k}}}-1}\right)\right)}^{\frac{1}{\left|S_{k}\right|!}}$$ (21)
  and *ξ*_*p*(*ik*)_ is a PA factor which can be calculated via Eq (14).
4. If *f*(*t*) = −In[(*ε*−1)/(*ε*^*y*^−1)] (*y* = *t*^*q*^; *ε* > 1), then *g*(*t*) = −In[(*ε*−1)/(*ε*^1−*y*^−1)], *f*^−1^(*t*) = {log_*ε*_[(*ε*−1+e^−*t*^)/e^−*t*^]}^1/*q*^, and *g*^−1^(*t*) = {1−log_*ε*_[(*ε*−1+e^−*t*^)/e^−*t*^]}^1/*q*^. A q-rung orthopair fuzzy Archimedean Frank power partitioned MM (qROFAFPPMM) operator is constructed according to Eq (11):
  $$\begin{array}{l} qROFAFPPMM^{\Delta }(Q_{1},Q_{2},\ldots ,Q_{n})=\langle {\left(1-\log_{\varepsilon }\left(1+\left(\varepsilon -1\right)/{\left(\prod_{k=1}^{N}\left(\left(\varepsilon -1\right)/\left(\varepsilon^{1-\tilde{\mu }}-1\right)\right)\right)}^{\frac{1}{N}}\right)\right)}^{1/q},\\ \;{\left(\log_{\varepsilon }\left(1+\left(\varepsilon -1\right)/{\left(\prod_{k=1}^{N}\left(\left(\varepsilon -1\right)/\left(\varepsilon^{\tilde{\nu }}-1\right)\right)\right)}^{\frac{1}{N}}\right)\right)}^{1/q}\rangle \end{array}$$ (22)
  where
  $$\tilde{\mu }=\log_{\varepsilon }\left(1+\left(\varepsilon -1\right)/{\left(\left(\varepsilon -1\right)/\left(\varepsilon^{\mu^{\prime \prime \prime }}-1\right)\right)}^{1/\sum_{i_{k}=1}^{\left|S_{k}\right|}\delta_{i_{k}}}\right)$$ (23)
  $$\tilde{\nu }=1-\log_{\varepsilon }\left(1+\left(\varepsilon -1\right)/{\left(\left(\varepsilon -1\right)/\left(\varepsilon^{1-\nu^{\prime \prime \prime }}-1\right)\right)}^{1/\sum_{i_{k}=1}^{\left|S_{k}\right|}\delta_{i_{k}}}\right)$$ (24)
  $$\mu^{\prime \prime \prime }=1-\log_{\varepsilon }\left(1+\left(\varepsilon -1\right)/{\left(\prod_{p\in P_{\left|S_{k}\right|}}\left(\left(\varepsilon -1\right)/\left(\varepsilon^{1-\mu^{\prime \prime }}-1\right)\right)\right)}^{\frac{1}{\left|S_{k}\right|!}}\right)$$ (25)
  $$\nu^{\prime \prime \prime }=\log_{\varepsilon }\left(1+\left(\varepsilon -1\right)/{\left(\prod_{p\in P_{\left|S_{k}\right|}}\left(\left(\varepsilon -1\right)/\left(\varepsilon^{\nu^{\prime \prime }}-1\right)\right)\right)}^{\frac{1}{\left|S_{k}\right|!}}\right)$$ (26)
  $$\mu^{\prime \prime }=\log_{\varepsilon }\left(1+\left(\varepsilon -1\right)/\prod_{i_{k}=1}^{\left|S_{k}\right|}{\left(\left(\varepsilon -1\right)/\left(\varepsilon^{\mu^{\prime }}-1\right)\right)}^{\delta_{i_{k}}}\right)$$ (27)
  $$\nu^{\prime \prime }=1-\log_{\varepsilon }\left(1+\left(\varepsilon -1\right)/\prod_{i_{k}=1}^{\left|S_{k}\right|}{\left(\left(\varepsilon -1\right)/\left(\varepsilon^{1-\nu^{\prime }}-1\right)\right)}^{\delta_{i_{k}}}\right)$$ (28)
  $$\mu^{\prime }=1-\log_{\varepsilon }\left(1+{\left(\varepsilon^{1-\mu_{p(i_{k})}^{q}}-1\right)}^{n\xi_{p(i_{k})}}/{\left(\varepsilon -1\right)}^{n\xi_{p(i_{k})}-1}\right)$$ (29)
  $$\nu^{\prime }=\log_{\varepsilon }\left(1+{\left(\varepsilon^{\nu_{p(i_{k})}^{q}}-1\right)}^{n\xi_{p(i_{k})}}/{\left(\varepsilon -1\right)}^{n\xi_{p(i_{k})}-1}\right)$$ (30)
  and *ξ*_*p*(*ik*)_ is a PA factor which can be calculated via Eq (14).

### 3.2. qROFWAPPMM operator

The qROFAPPMM operator has advantages in having satisfying generality and flexibility, capturing the complex interrelationships among qROFNs, and reducing the negative effect of unreasonable qROFNs on the aggregation results. But it does not consider the relative importance of each aggregated qROFN. To overcome this limitation, weights are introduced and a qROFWAPPMM operator is presented. The formal definition of this operator is as follow:

**Definition 11**. On the basis of Definition 10, let *w*_1_, *w*_2_, …, *w*_*n*_ be respectively the weights of *Q*_1_, *Q*_2_, …, *Q*_*n*_ such that 0 ≤ *w*_1_, *w*_2_, …, *w*_*n*_ ≤ 1 and *w*_1_+*w*_2_+…+*w*_*n*_ = 1. Then the aggregation function

$$qROFWAPPMM^{\Delta }(Q_{1},Q_{2},\ldots ,Q_{n})=\frac{1}{N}\left(\overset{N}{\underset{k=1}{⊕}}{\left(\frac{1}{\left|S_{k}\right|!}\underset{p\in P_{\left|S_{k}\right|}}{⊕}\overset{\left|S_{k}\right|}{\underset{i_{k}=1}{⊗}}{\left(\frac{nw_{p(i_{k})}\left(1+T(Q_{p(i_{k})})\right)}{\sum_{j=1}^{n}\left(w_{j}\left(1+T(Q_{j})\right)\right)}Q_{p(i_{k})}\right)}^{\delta_{i_{k}}}\right)}^{\frac{1}{\sum_{i_{k}=1}^{\left|S_{k}\right|}\delta_{i_{k}}}}\right)$$ (31)

is called the qROFWAPPMM operator. In this operator, the functions of *δ*_*ik*_ (*i*_*k*_ = 1, 2, …, |***S***_*k*_|) are the same as the functions of *δ*_*ik*_ in the qROFAPPMM operator (see Eq (11)).

According to Eqs (1)‒(4) and (31), the following theorem is obtained:

**Theorem 4**. Let *Q*_1_, *Q*_2_, …, *Q*_*n*_ (*Q*_*i*_ = <*μ*_*i*_, *ν*_*i*_>, *i* = 1, 2, …, *n*) be *n* qROFNs (*q* = 1, 2, 3, …) and (*Q*_1_, *Q*_2_, …, *Q*_*n*_) be a collection of *Q*_1_, *Q*_2_, …, *Q*_*n*_. Then

$$qROFWAPPMM^{\Delta }(Q_{1},Q_{2},\ldots ,Q_{n})=\langle \mu ,\;\nu \rangle$$ (32)

and it is still a qROFN, where

$$\mu =g^{-1}\left(\frac{1}{N}\sum_{k=1}^{N}g\left(f^{-1}\left(\frac{1}{\sum_{i_{k}=1}^{\left|S_{k}\right|}\delta_{i_{k}}}f\left(g^{-1}\left(\frac{1}{\left|S_{k}\right|!}\sum_{p\in P_{\left|S_{k}\right|}}g\left(f^{-1}\left(\sum_{i_{k}=1}^{\left|S_{k}\right|}\left(\delta_{i_{k}}f\left(g^{-1}\left(\left(n\left(w_{p(i_{k})}\xi_{p(i_{k})}\right)/\sum_{t=1}^{n}\left(w_{t}\xi_{t}\right)\right)g(\mu_{p(i_{k})})\right)\right)\right)\right)\right)\right)\right)\right)\right)\right)$$ (33)

$$\nu =f^{-1}\left(\frac{1}{N}\sum_{k=1}^{N}f\left(g^{-1}\left(\frac{1}{\sum_{i_{k}=1}^{\left|S_{k}\right|}\delta_{i_{k}}}g\left(f^{-1}\left(\frac{1}{\left|S_{k}\right|!}\sum_{p\in P_{\left|S_{k}\right|}}f\left(g^{-1}\left(\sum_{i_{k}=1}^{\left|S_{k}\right|}\left(\delta_{i_{k}}g\left(f^{-1}\left(\left(n\left(w_{p(i_{k})}\xi_{p(i_{k})}\right)/\sum_{t=1}^{n}\left(w_{t}\xi_{t}\right)\right)f(\nu_{p(i_{k})})\right)\right)\right)\right)\right)\right)\right)\right)\right)\right)$$ (34)

and *ξ*_*p*(*ik*)_ and *ξ*_*t*_ are two PA factors which can be calculated via Eq (14).

The proof of Theorem 4 is similar to the proof of Theorem 1 (see Appendix A in S1 File) and is omitted here. It is worth nothing that the qROFWAPPMM operator no longer has the properties of idempotency and boundedness due to the introduce of weights.

For Eq (32), if the additive generators of Algebraic, Einstein, Hamacher, and Frank T-norms and T-conorms are assigned to *f*, then four specific operators can be respectively constructed as follows:

1. If *f*(*t*) = −In*t*^*q*^, then *g*(*t*) = −In(1−*t*^*q*^), *f*^−1^(*t*) = (e^−*t*^)^1/*q*^, and *g*^−1^(*t*) = (1−e^−*t*^)^1/*q*^. A q-rung orthopair fuzzy weighted Archimedean Algebraic power partitioned MM (qROFWAAPPMM) operator is constructed according to Eq (32):
  $$\begin{array}{l} qROFWAAPPMM^{\Delta }(Q_{1},Q_{2},\ldots ,Q_{n})=\\ \langle {\left(1-{\left(\prod_{k=1}^{N}\left(1-{\left(1-{\left(\prod_{p\in P_{\left|S_{k}\right|}}\left(1-\prod_{i_{k}=1}^{\left|S_{k}\right|}{\left(1-{\left(1-\mu_{p(i_{k})}^{q}\right)}^{n\left(w_{p(i_{k})}\xi_{p(i_{k})}\right)/\sum_{t=1}^{n}\left(w_{t}\xi_{t}\right)}\right)}^{\delta_{i_{k}}}\right)\right)}^{\frac{1}{\left|S_{k}\right|!}}\right)}^{\frac{1}{\sum_{i_{k}=1}^{\left|S_{k}\right|}\delta_{i_{k}}}}\right)\right)}^{\frac{1}{N}}\right)}^{1/q},\\ {\left({\left(\prod_{k=1}^{N}\left(1-{\left(1-{\left(\prod_{p\in P_{\left|S_{k}\right|}}\left(1-\prod_{i_{k}=1}^{\left|S_{k}\right|}{\left(1-\nu_{p(i_{k})}^{qn\left(w_{p(i_{k})}\xi_{p(i_{k})}\right)/\sum_{t=1}^{n}\left(w_{t}\xi_{t}\right)}\right)}^{\delta_{i_{k}}}\right)\right)}^{\frac{1}{\left|S_{k}\right|!}}\right)}^{\frac{1}{\sum_{i_{k}=1}^{\left|S_{k}\right|}\delta_{i_{k}}}}\right)\right)}^{\frac{1}{N}}\right)}^{1/q}\rangle \end{array}$$ (35)
  where *ξ*_*p*(*ik*)_ and *ξ*_*t*_ are two PA factors which can be calculated via Eq (14).
2. If *f*(*t*) = In[(2−*t*^*q*^)/*t*^*q*^], then *g*(*t*) = In[(1+*t*^*q*^)/(1−*t*^*q*^)], *f*^−1^(*t*) = [2/(e^*t*^+1)]^1/*q*^, and *g*^−1^(*t*) = [(e^*t*^−1)/(e^*t*^+1)]^1/*q*^. A q-rung orthopair fuzzy weighted Archimedean Einstein power partitioned MM (qROFWAEPPMM) operator is constructed according to Eq (32):
  $$\begin{array}{l} qROFWAEPPMM^{\Delta }(Q_{1},Q_{2},\ldots ,Q_{n})=\langle {\left(\frac{{\left(\prod_{k=1}^{N}\left(\frac{{\left(\mu^{\prime }+3\right)}^{1/\sum_{i_{k}=1}^{\left|S_{k}\right|}\delta_{i_{k}}}+3{\left(\mu^{\prime }-1\right)}^{1/\sum_{i_{k}=1}^{\left|S_{k}\right|}\delta_{i_{k}}}}{{\left(\mu^{\prime }+3\right)}^{1/\sum_{i_{k}=1}^{\left|S_{k}\right|}\delta_{i_{k}}}-{\left(\mu^{\prime }-1\right)}^{1/\sum_{i_{k}=1}^{\left|S_{k}\right|}\delta_{i_{k}}}}\right)\right)}^{\frac{1}{N}}-1}{{\left(\prod_{k=1}^{N}\left(\frac{{\left(\mu^{\prime }+3\right)}^{1/\sum_{i_{k}=1}^{\left|S_{k}\right|}\delta_{i_{k}}}+3{\left(\mu^{\prime }-1\right)}^{1/\sum_{i_{k}=1}^{\left|S_{k}\right|}\delta_{i_{k}}}}{{\left(\mu^{\prime }+3\right)}^{1/\sum_{i_{k}=1}^{\left|S_{k}\right|}\delta_{i_{k}}}-{\left(\mu^{\prime }-1\right)}^{1/\sum_{i_{k}=1}^{\left|S_{k}\right|}\delta_{i_{k}}}}\right)\right)}^{\frac{1}{N}}+1}\right)}^{1/q},\\ \;{\left(2/\left({\left(\prod_{k=1}^{N}\left(\frac{{\left(\nu^{\prime }+3\right)}^{1/\sum_{i_{k}=1}^{\left|S_{k}\right|}\delta_{i_{k}}}+3{\left(\nu^{\prime }-1\right)}^{1/\sum_{i_{k}=1}^{\left|S_{k}\right|}\delta_{i_{k}}}}{{\left(\nu^{\prime }+3\right)}^{1/\sum_{i_{k}=1}^{\left|S_{k}\right|}\delta_{i_{k}}}-{\left(\nu^{\prime }-1\right)}^{1/\sum_{i_{k}=1}^{\left|S_{k}\right|}\delta_{i_{k}}}}\right)\right)}^{\frac{1}{N}}+1\right)\right)}^{1/q}\rangle \end{array}$$ (36)
  where
  $$\mu^{\prime }={\left(\prod_{p\in P_{\left|S_{k}\right|}}\left(\frac{\prod_{i_{k}=1}^{\left|S_{k}\right|}{\left(\frac{{\left(1+\mu_{p(i_{k})}^{q}\right)}^{n\left(w_{p(i_{k})}\xi_{p(i_{k})}\right)/\sum_{t=1}^{n}\left(w_{t}\xi_{t}\right)}+3{\left(1-\mu_{p(i_{k})}^{q}\right)}^{n\left(w_{p(i_{k})}\xi_{p(i_{k})}\right)/\sum_{t=1}^{n}\left(w_{t}\xi_{t}\right)}}{{\left(1+\mu_{p(i_{k})}^{q}\right)}^{n\left(w_{p(i_{k})}\xi_{p(i_{k})}\right)/\sum_{t=1}^{n}\left(w_{t}\xi_{t}\right)}-{\left(1-\mu_{p(i_{k})}^{q}\right)}^{n\left(w_{p(i_{k})}\xi_{p(i_{k})}\right)/\sum_{t=1}^{n}\left(w_{t}\xi_{t}\right)}}\right)}^{\delta_{i_{k}}}+3}{\prod_{i_{k}=1}^{\left|S_{k}\right|}{\left(\frac{{\left(1+\mu_{p(i_{k})}^{q}\right)}^{n\left(w_{p(i_{k})}\xi_{p(i_{k})}\right)/\sum_{t=1}^{n}\left(w_{t}\xi_{t}\right)}+3{\left(1-\mu_{p(i_{k})}^{q}\right)}^{n\left(w_{p(i_{k})}\xi_{p(i_{k})}\right)/\sum_{t=1}^{n}\left(w_{t}\xi_{t}\right)}}{{\left(1+\mu_{p(i_{k})}^{q}\right)}^{n\left(w_{p(i_{k})}\xi_{p(i_{k})}\right)/\sum_{t=1}^{n}\left(w_{t}\xi_{t}\right)}-{\left(1-\mu_{p(i_{k})}^{q}\right)}^{n\left(w_{p(i_{k})}\xi_{p(i_{k})}\right)/\sum_{t=1}^{n}\left(w_{t}\xi_{t}\right)}}\right)}^{\delta_{i_{k}}}-1}\right)\right)}^{\frac{1}{\left|S_{k}\right|!}}$$ (37)
  $$\nu^{\prime }={\left(\prod_{p\in P_{\left|S_{k}\right|}}\left(\frac{\prod_{i_{k}=1}^{\left|S_{k}\right|}{\left(\frac{{\left(2-\nu_{p(i_{k})}^{q}\right)}^{n\left(w_{p(i_{k})}\xi_{p(i_{k})}\right)/\sum_{t=1}^{n}\left(w_{t}\xi_{t}\right)}+3\nu_{p(i_{k})}^{qn\left(w_{p(i_{k})}\xi_{p(i_{k})}\right)/\sum_{t=1}^{n}\left(w_{t}\xi_{t}\right)}}{{\left(2-\nu_{p(i_{k})}^{q}\right)}^{n\left(w_{p(i_{k})}\xi_{p(i_{k})}\right)/\sum_{t=1}^{n}\left(w_{t}\xi_{t}\right)}-\nu_{p(i_{k})}^{qn\left(w_{p(i_{k})}\xi_{p(i_{k})}\right)/\sum_{t=1}^{n}\left(w_{t}\xi_{t}\right)}}\right)}^{\delta_{i_{k}}}+3}{\prod_{i_{k}=1}^{\left|S_{k}\right|}{\left(\frac{{\left(2-\nu_{p(i_{k})}^{q}\right)}^{n\left(w_{p(i_{k})}\xi_{p(i_{k})}\right)/\sum_{t=1}^{n}\left(w_{t}\xi_{t}\right)}+3\nu_{p(i_{k})}^{qn\left(w_{p(i_{k})}\xi_{p(i_{k})}\right)/\sum_{t=1}^{n}\left(w_{t}\xi_{t}\right)}}{{\left(2-\nu_{p(i_{k})}^{q}\right)}^{n\left(w_{p(i_{k})}\xi_{p(i_{k})}\right)/\sum_{t=1}^{n}\left(w_{t}\xi_{t}\right)}-\nu_{p(i_{k})}^{qn\left(w_{p(i_{k})}\xi_{p(i_{k})}\right)/\sum_{t=1}^{n}\left(w_{t}\xi_{t}\right)}}\right)}^{\delta_{i_{k}}}-1}\right)\right)}^{\frac{1}{\left|S_{k}\right|!}}$$ (38)
  and *ξ*_*p*(*ik*)_ and *ξ*_*t*_ are two PA factors which can be calculated via Eq (14).
3. If *f*(*t*) = In{[*λ*+(1−*λ*)*t*^*q*^]/*t*^*q*^} (*λ* > 0), then *g*(*t*) = In{[*λ*+(1−*λ*)(1−*t*^*q*^)]/(1−*t*^*q*^)}, *f*^−1^(*t*) = [*λ*/(e^*t*^+*λ*−1)]^1/*q*^, and *g*^−1^(*t*) = [(e^*t*^−1)/ (e^*t*^+*λ*−1)]^1/*q*^. A q-rung orthopair fuzzy weighted Archimedean Hamacher power partitioned MM (qROFWAHPPMM) operator is constructed according to Eq (32):
  $$\begin{array}{l} qROFWAHPPMM^{\Delta }(Q_{1},Q_{2},\ldots ,Q_{n})=\langle {\left(\frac{{\left(\prod_{k=1}^{N}\left(\frac{{\left(\mu^{\prime }+\lambda^{2}-1\right)}^{1/\sum_{i_{k}=1}^{\left|S_{k}\right|}\delta_{i_{k}}}+(\lambda^{2}-1){\left(\mu^{\prime }-1\right)}^{1/\sum_{i_{k}=1}^{\left|S_{k}\right|}\delta_{i_{k}}}}{{\left(\mu^{\prime }+\lambda^{2}-1\right)}^{1/\sum_{i_{k}=1}^{\left|S_{k}\right|}\delta_{i_{k}}}-{\left(\mu^{\prime }-1\right)}^{1/\sum_{i_{k}=1}^{\left|S_{k}\right|}\delta_{i_{k}}}}\right)\right)}^{\frac{1}{N}}-1}{{\left(\prod_{k=1}^{N}\left(\frac{{\left(\mu^{\prime }+\lambda^{2}-1\right)}^{1/\sum_{i_{k}=1}^{\left|S_{k}\right|}\delta_{i_{k}}}+(\lambda^{2}-1){\left(\mu^{\prime }-1\right)}^{1/\sum_{i_{k}=1}^{\left|S_{k}\right|}\delta_{i_{k}}}}{{\left(\mu^{\prime }+\lambda^{2}-1\right)}^{1/\sum_{i_{k}=1}^{\left|S_{k}\right|}\delta_{i_{k}}}-{\left(\mu^{\prime }-1\right)}^{1/\sum_{i_{k}=1}^{\left|S_{k}\right|}\delta_{i_{k}}}}\right)\right)}^{\frac{1}{N}}+\lambda -1}\right)}^{1/q},\\ \;{\left(\lambda /\left({\left(\prod_{k=1}^{N}\left(\frac{{\left(\nu^{\prime }+\lambda^{2}-1\right)}^{1/\sum_{i_{k}=1}^{\left|S_{k}\right|}\delta_{i_{k}}}+(\lambda^{2}-1){\left(\nu^{\prime }-1\right)}^{1/\sum_{i_{k}=1}^{\left|S_{k}\right|}\delta_{i_{k}}}}{{\left(\nu^{\prime }+\lambda^{2}-1\right)}^{1/\sum_{i_{k}=1}^{\left|S_{k}\right|}\delta_{i_{k}}}-{\left(\nu^{\prime }-1\right)}^{1/\sum_{i_{k}=1}^{\left|S_{k}\right|}\delta_{i_{k}}}}\right)\right)}^{\frac{1}{N}}+\lambda -1\right)\right)}^{1/q}\rangle \end{array}$$ (39)
  where
  $$\mu^{\prime }={\left(\prod_{p\in P_{\left|S_{k}\right|}}\left(\frac{\prod_{i_{k}=1}^{\left|S_{k}\right|}{\left(\frac{{\left(\lambda +(1-\lambda )(1-\mu_{p(i_{k})}^{q})\right)}^{n\left(w_{p(i_{k})}\xi_{p(i_{k})}\right)/\sum_{t=1}^{n}\left(w_{t}\xi_{t}\right)}+(\lambda^{2}-1){\left(1-\mu_{p(i_{k})}^{q}\right)}^{n\left(w_{p(i_{k})}\xi_{p(i_{k})}\right)/\sum_{t=1}^{n}\left(w_{t}\xi_{t}\right)}}{{\left(\lambda +(1-\lambda )(1-\mu_{p(i_{k})}^{q})\right)}^{n\left(w_{p(i_{k})}\xi_{p(i_{k})}\right)/\sum_{t=1}^{n}\left(w_{t}\xi_{t}\right)}-{\left(1-\mu_{p(i_{k})}^{q}\right)}^{n\left(w_{p(i_{k})}\xi_{p(i_{k})}\right)/\sum_{t=1}^{n}\left(w_{t}\xi_{t}\right)}}\right)}^{\delta_{i_{k}}}+\lambda^{2}-1}{\prod_{i_{k}=1}^{\left|S_{k}\right|}{\left(\frac{{\left(\lambda +(1-\lambda )(1-\mu_{p(i_{k})}^{q})\right)}^{n\left(w_{p(i_{k})}\xi_{p(i_{k})}\right)/\sum_{t=1}^{n}\left(w_{t}\xi_{t}\right)}+(\lambda^{2}-1){\left(1-\mu_{p(i_{k})}^{q}\right)}^{n\left(w_{p(i_{k})}\xi_{p(i_{k})}\right)/\sum_{t=1}^{n}\left(w_{t}\xi_{t}\right)}}{{\left(\lambda +(1-\lambda )(1-\mu_{p(i_{k})}^{q})\right)}^{n\left(w_{p(i_{k})}\xi_{p(i_{k})}\right)/\sum_{t=1}^{n}\left(w_{t}\xi_{t}\right)}-{\left(1-\mu_{p(i_{k})}^{q}\right)}^{n\left(w_{p(i_{k})}\xi_{p(i_{k})}\right)/\sum_{t=1}^{n}\left(w_{t}\xi_{t}\right)}}\right)}^{\delta_{i_{k}}}-1}\right)\right)}^{\frac{1}{\left|S_{k}\right|!}}$$ (40)
  $$\nu^{\prime }={\left(\prod_{p\in P_{\left|S_{k}\right|}}\left(\frac{\prod_{i_{k}=1}^{\left|S_{k}\right|}{\left(\frac{{\left(\lambda +(1-\lambda )\nu_{p(i_{k})}^{q}\right)}^{n\left(w_{p(i_{k})}\xi_{p(i_{k})}\right)/\sum_{t=1}^{n}\left(w_{t}\xi_{t}\right)}+(\lambda^{2}-1)\nu_{p(i_{k})}^{qn\left(w_{p(i_{k})}\xi_{p(i_{k})}\right)/\sum_{t=1}^{n}\left(w_{t}\xi_{t}\right)}}{{\left(\lambda +(1-\lambda )\nu_{p(i_{k})}^{q}\right)}^{n\left(w_{p(i_{k})}\xi_{p(i_{k})}\right)/\sum_{t=1}^{n}\left(w_{t}\xi_{t}\right)}-\nu_{p(i_{k})}^{qn\left(w_{p(i_{k})}\xi_{p(i_{k})}\right)/\sum_{t=1}^{n}\left(w_{t}\xi_{t}\right)}}\right)}^{\delta_{i_{k}}}+\lambda^{2}-1}{\prod_{i_{k}=1}^{\left|S_{k}\right|}{\left(\frac{{\left(\lambda +(1-\lambda )\nu_{p(i_{k})}^{q}\right)}^{n\left(w_{p(i_{k})}\xi_{p(i_{k})}\right)/\sum_{t=1}^{n}\left(w_{t}\xi_{t}\right)}+(\lambda^{2}-1)\nu_{p(i_{k})}^{qn\left(w_{p(i_{k})}\xi_{p(i_{k})}\right)/\sum_{t=1}^{n}\left(w_{t}\xi_{t}\right)}}{{\left(\lambda +(1-\lambda )\nu_{p(i_{k})}^{q}\right)}^{n\left(w_{p(i_{k})}\xi_{p(i_{k})}\right)/\sum_{t=1}^{n}\left(w_{t}\xi_{t}\right)}-\nu_{p(i_{k})}^{qn\left(w_{p(i_{k})}\xi_{p(i_{k})}\right)/\sum_{t=1}^{n}\left(w_{t}\xi_{t}\right)}}\right)}^{\delta_{i_{k}}}-1}\right)\right)}^{\frac{1}{\left|S_{k}\right|!}}$$ (41)
  and *ξ*_*p*(*ik*)_ and *ξ*_*t*_ are two PA factors which can be calculated via Eq (14).
4. If *f*(*t*) = −In[(*ε*−1)/(*ε*^*y*^−1)] (*y* = *t*^*q*^; *ε* > 1), then *g*(*t*) = −In[(*ε*−1)/(*ε*^1−*y*^−1)], *f*^−1^(*t*) = {log_*ε*_[(*ε*−1+e^−*t*^)/e^−*t*^]}^1/*q*^, and *g*^−1^(*t*) = {1− log_*ε*_[(*ε*−1+e^−*t*^)/e^−*t*^]}^1/*q*^. A q-rung orthopair fuzzy weighted Archimedean Frank power partitioned MM (qROFWAFPPMM) operator is constructed according to Eq (32):
  $$\begin{array}{l} qROFWAFPPMM^{\Delta }(Q_{1},Q_{2},\ldots ,Q_{n})=\langle {\left(1-\log_{\varepsilon }\left(1+\left(\varepsilon -1\right)/{\left(\prod_{k=1}^{N}\left(\left(\varepsilon -1\right)/\left(\varepsilon^{1-\tilde{\mu }}-1\right)\right)\right)}^{\frac{1}{N}}\right)\right)}^{1/q},\\ \;{\left(\log_{\varepsilon }\left(1+\left(\varepsilon -1\right)/{\left(\prod_{k=1}^{N}\left(\left(\varepsilon -1\right)/\left(\varepsilon^{\tilde{\nu }}-1\right)\right)\right)}^{\frac{1}{N}}\right)\right)}^{1/q}\rangle \end{array}$$ (42)
  where
  $$\tilde{\mu }=\log_{\varepsilon }\left(1+\left(\varepsilon -1\right)/{\left(\left(\varepsilon -1\right)/\left(\varepsilon^{\mu^{\prime \prime \prime }}-1\right)\right)}^{1/\sum_{i_{k}=1}^{\left|S_{k}\right|}\delta_{i_{k}}}\right)$$ (43)
  $$\tilde{\nu }=1-\log_{\varepsilon }\left(1+\left(\varepsilon -1\right)/{\left(\left(\varepsilon -1\right)/\left(\varepsilon^{1-\nu^{\prime \prime \prime }}-1\right)\right)}^{1/\sum_{i_{k}=1}^{\left|S_{k}\right|}\delta_{i_{k}}}\right)$$ (44)
  $$\mu^{\prime \prime \prime }=1-\log_{\varepsilon }\left(1+\left(\varepsilon -1\right)/{\left(\prod_{p\in P_{\left|S_{k}\right|}}\left(\left(\varepsilon -1\right)/\left(\varepsilon^{1-\mu^{\prime \prime }}-1\right)\right)\right)}^{\frac{1}{\left|S_{k}\right|!}}\right)$$ (45)
  $$\nu^{\prime \prime \prime }=\log_{\varepsilon }\left(1+\left(\varepsilon -1\right)/{\left(\prod_{p\in P_{\left|S_{k}\right|}}\left(\left(\varepsilon -1\right)/\left(\varepsilon^{\nu^{\prime \prime }}-1\right)\right)\right)}^{\frac{1}{\left|S_{k}\right|!}}\right)$$ (46)
  $$\mu^{\prime \prime }=\log_{\varepsilon }\left(1+\left(\varepsilon -1\right)/\prod_{i_{k}=1}^{\left|S_{k}\right|}{\left(\left(\varepsilon -1\right)/\left(\varepsilon^{\mu^{\prime }}-1\right)\right)}^{\delta_{i_{k}}}\right)$$ (47)
  $$\nu^{\prime \prime }=1-\log_{\varepsilon }\left(1+\left(\varepsilon -1\right)/\prod_{i_{k}=1}^{\left|S_{k}\right|}{\left(\left(\varepsilon -1\right)/\left(\varepsilon^{1-\nu^{\prime }}-1\right)\right)}^{\delta_{i_{k}}}\right)$$ (48)
  $$\mu^{\prime }=1-\log_{\varepsilon }\left(1+{\left(\varepsilon^{1-\mu_{p(i_{k})}^{q}}-1\right)}^{n\left(w_{p(i_{k})}\xi_{p(i_{k})}\right)/\sum_{t=1}^{n}\left(w_{t}\xi_{t}\right)}/{\left(\varepsilon -1\right)}^{n\left(w_{p(i_{k})}\xi_{p(i_{k})}\right)/\sum_{t=1}^{n}\left(w_{t}\xi_{t}\right)-1}\right)$$ (49)
  $$\nu^{\prime }=\log_{\varepsilon }\left(1+{\left(\varepsilon^{\nu_{p(i_{k})}^{q}}-1\right)}^{n\left(w_{p(i_{k})}\xi_{p(i_{k})}\right)/\sum_{t=1}^{n}\left(w_{t}\xi_{t}\right)}/{\left(\varepsilon -1\right)}^{n\left(w_{p(i_{k})}\xi_{p(i_{k})}\right)/\sum_{t=1}^{n}\left(w_{t}\xi_{t}\right)-1}\right)$$ (50)
  and *ξ*_*p*(*ik*)_ and *ξ*_*t*_ are two PA factors which can be calculated via Eq (14).

## 4. MCGDM method

In this section, a MCGDM method based on the qROFWAPPMM operator is proposed to solve the MCGDM problems based on qROFNs.

In general, a MCGDM problem based on qROFNs can be described via a set of alternatives ***A*** = {*A*_1_, *A*_2_, …, *A*_*m*_}, a set of criteria ***C*** = {*C*_1_, *C*_2_, …, *C*_*n*_} such that ***C*** is divided into *N* partitions ***C***_*k*_ = {*C*_1_, *C*_2_, …, *C*_|***C****k*|_} (*k* = 1, 2, …, *N*) and there are interrelationships among different criteria in each ***C***_*k*_ whereas the criteria in different ***C***_*k*_ are independent of each other, a vector of weights of criteria ***w*** = [*w*_1_, *w*_2_, …, *w*_*n*_] such that 0 ≤ *w*_1_, *w*_2_, …, *w*_*n*_ ≤ 1, *w*_1_+*w*_2_+…+*w*_*n*_ = 1, and each element respectively denotes the relative importance of *C*_1_, *C*_2_, …, *C*_*n*_, a set of experts ***E*** = {*E*_1_, *E*_2_, …, *E*_*M*_}, a vector of weights of experts ***ϖ*** = [*ϖ*_1_, *ϖ*_2_, …, *ϖ*_*M*_] such that 0 ≤ *ϖ*_1_, *ϖ*_2_, …, *ϖ*_*M*_ ≤ 1, *ϖ*_1_+*ϖ*_2_+…+*ϖ*_*M*_ = 1, and each element respectively denotes the relative importance of *E*_1_, *E*_2_, …, *E*_*M*_, and *M* q-rung orthopair fuzzy decision matrices ***M***_*h*_ = [*Q*_*h*,*i*,*j*_]_*m*×*n*_ (*h* = 1, 2,…, *M*; *i* = 1, 2,…, *m*; *j* = 1, 2,…, *n*) such that *Q*_*h*,*i*,*j*_ = <*μ*_*h*,*i*,*j*_, *ν*_*h*,*i*,*j*_> is a qROFN which denotes the evaluation value of *C*_*j*_ with respect to *A*_*i*_ provided by *E*_*k*_. Based on these components, the MCGDM problem can be described as: Determining the optimal alternative with the help of a ranking of the elements of ***A*** based on ***C***, ***M***_*h*_, ***w***, and ***ϖ***. Using the qROFWAPPMM operator, the problem is solved according to the following steps:

1. Normalise the q-rung orthopair fuzzy decision matrices ***M***_*h*_. Generally, a MCGDM problem may contain two types of criteria, i.e. benefit and cost criteria, which affect the results of decision making positively and negatively, respectively. To eliminate the negative effect, the q-rung orthopair fuzzy decision matrices ***M***_*h*_ = [*Q*_*h*,*i*,*j*_]_*m*×*n*_ = <*μ*_*h*,*i*,*j*_, *ν*_*h*,*i*,*j*_> are normalised as
  $$M_{N,h}={\left[Q_{h,i,j}\right]}_{m\times n}=\left\{ \begin{array}{l} {\left[\langle \mu_{h,i,j},\nu_{h,i,j}\rangle \right]}_{m\times n},\;\mathrm{if}\;C_{j}\;\mathrm{is}\;a\;\mathrm{benefit}\;\mathrm{criterion}\\ {\left[\langle \nu_{h,i,j},\mu_{h,i,j}\rangle \right]}_{m\times n},\;\mathrm{if}\;C_{j}\;\mathrm{is}\;a\;\mathrm{cost}\;\mathrm{criterion} \end{array}\right.$$ (51)
2. Calculate the power weights of *Q*_*h*,*i*,*j*_. The power weights of *Q*_*h*,*i*,*j*_ are computed using
  $$W_{h,i,j}=\left(\varpi_{h}\xi_{h}\right)/\sum_{z=1}^{M}\left(\varpi_{z}\xi_{z}\right)=\left(\varpi_{h}\left(1+\sum_{x=1,x\neq h}^{M}\left(1-D(Q_{h,i,j},\;Q_{x,i,j})\right)\right)\right)/\sum_{z=1}^{M}\left(\varpi_{z}\left(1+\sum_{y=1,y\neq z}^{M}\left(1-D(Q_{z,i,j},\;Q_{y,i,j})\right)\right)\right)$$ (52)
  where *D*(*Q*_*h*,*i*,*j*_, *Q*_*x*,*i*,*j*_) (*D*(*Q*_*z*,*i*,*j*_, *Q*_*y*,*i*,*j*_)) is the Minkowski-type distance between *Q*_*h*,*i*,*j*_ and *Q*_*x*,*i*,*j*_ (*Q*_*z*,*i*,*j*_ and *Q*_*y*,*i*,*j*_) that can be computed via the Equation in Definition 5.
3. Calculate the collective values of *Q*_*h*,*i*,*j*_. Taking the normalised q-rung orthopair fuzzy decision matrices ***M***_**N**,*h*_ and the expert weight vector ***ϖ*** as input, the collective values of *Q*_*h*,*i*,*j*_ are computed using
  $$Q_{i,j}=\langle \mu_{i,j},\nu_{i,j}\rangle =qROFWAPPMM^{\Delta }(Q_{1,i,j},Q_{2,i,j},\ldots ,Q_{M,i,j})$$ (53)
  where *qROFWAPPMM* is an arbitrary specific qROFWAPPMM operator, such as the qROFWAAPPMM operator in Eq (35), the qROFWAEPPMM operator in Eq (36), the qROFWAHPPMM operator in Eq (39), and the qROFWAFPPMM operator in Eq (42), and the values of the elements in **Δ** = (*δ*_1_, *δ*_2_, …, *δ*_*M*_) (It is worth nothing that there is only one partition) are determined via identifying the interrelationships among different experts. In general, all experts should be mutually independent. Thus *δ*_1_ > 0 and *δ*_2_ = *δ*_3_ = … = *δ*_*M*_ = 0.
4. Calculate the power weights of *Q*_*i*,*j*_. The power weights of *Q*_*i*,*j*_ are computed using
  $$W_{i,j}=\left(w_{j}\xi_{j}\right)/\sum_{t=1}^{n}\left(w_{t}\xi_{t}\right)=\left(w_{j}\left(1+\sum_{r=1,r\neq j}^{n}\left(1-D(Q_{i,j},\;Q_{i,r})\right)\right)\right)/\sum_{t=1}^{n}\left(w_{t}\left(1+\sum_{s=1,s\neq t}^{n}\left(1-D(Q_{i,t},\;Q_{i,s})\right)\right)\right)$$ (54)
  where *D*(*Q*_*i*,*j*_, *Q*_*i*,*r*_) (*D*(*Q*_*i*,*r*_, *Q*_*i*,*s*_)) is the Minkowski-type distance between *Q*_*i*,*j*_ and *Q*_*i*,*r*_ (*Q*_*i*,*t*_ and *Q*_*i*,*s*_) that can be computed via the Equation in Definition 5.
5. Calculate the collective values of *Q*_*i*,*j*_. On the basis of the *N* partitions ***C***_*k*_ = {*C*_1_, *C*_2_, …, *C*_|***C****k*|_}, suppose ***S***_*i*_ = {*Q*_*i*,1_, *Q*_*i*,2_, …, *Q*_*i*,*n*_} is an ordered set of *Q*_*i*,1_, *Q*_*i*,2_, …, *Q*_*i*,*n*_, ***S***_*i*,*k*_ = {*Q*_*i*,1_, *Q*_*i*,2_, …, *Q*_*i*,|***S****k*|_} are *N* partitions of ***S*** that correspond to ***C***_*k*_, *δ*_1_, *δ*_2_, …, *δ*_|***S****i*,*k*|_ (*k* = 1, 2, …, *N* and *δ*_1_, *δ*_2_, …, *δ*_|***S****i*,*k*|_ ≥ 0 but not at the same time *δ*_1_ = *δ*_2_ = … = *δ*_|***S****i*,*k*|_ = 0) are |***S***_*i*,*k*_| real numbers that respectively correspond to *Q*_*i*,1_, *Q*_*i*,2_, …, *Q*_*i*,|***S****k*|_, **Δ**_*k*_ = (*δ*_1_, *δ*_2_, …, *δ*_|***S****i*,*k*|_) is a collection of *δ*_1_, *δ*_2_, …, *δ*_|***S****i*,*k*|_, **Δ** = (**Δ**_1_, **Δ**_2_, …, **Δ**_*N*_) is a collection of **Δ**_1_, **Δ**_2_, …, **Δ**_*N*_, *p*(*i*_*k*_) is a permutation of (1, 2,…, |***S***_*i*,*k*_|), and ***P***_|***S****i*,*k*|_ is a set of all permutations of (1, 2,…, |***S***_*i*,*k*_|). Then the collective values of *Q*_*i*,*j*_ are computed using
  $$Q_{i}=\langle \mu_{i},\nu_{i}\rangle =qROFWAPPMM^{\Delta }(Q_{i,1},Q_{i,2},\ldots ,Q_{i,n})$$ (55)
  where *qROFWAPPMM* is the same specific qROFWAPPMM operator used in Eq (53), and the values of the elements in **Δ** = (**Δ**_1_, **Δ**_2_, …, **Δ**_*N*_) (**Δ**_*k*_ = (*δ*_1_, *δ*_2_, …, *δ*_|***S****i*,*k*|_)) are determined via identifying the interrelationships among different criteria in ***C***_*k*_. When all criteria in ***C***_*k*_ are mutually independent, then *δ*_1_ > 0 and *δ*_2_ = *δ*_3_ = … = *δ*_|***S****i*,*k*|_ = 0; When there are interrelationships between any two criteria in ***C***_*k*_, *δ*_1_, *δ*_2_ > 0 and *δ*_3_ = *δ*_4_ = … = *δ*_|***S****i*,*k*|_ = 0; When there are interrelationships among any *d* (*d* = 3, 4, …, *n*) criteria in ***C***_*k*_, *δ*_1_, *δ*_2_, …, *δ*_*d*_ > 0 and *δ*_*d*+1_ = *δ*_*d*+2_ = … = *δ*_|***S****i*,*k*|_ = 0.
6. Calculate the scores and accuracies of *Q*_*i*_. The scores and accuracies of *Q*_*i*_ can be respectively computed using the Equations in Definitions 2 and 3.
7. Generate a ranking of *A*_*i*_. On the basis of the scores and accuracies of *Q*_*i*_, a ranking of *A*_*i*_ can be generated according to the comparison rules in Definition 4.
8. Determine the optimal alternative. The optimal alternative is determined with the help of the ranking.

## 5. Example, experiments, and comparisons

In this section, a numerical example is firstly used to illustrate the working process of the proposed MCGDM method. Then a set of experiments are carried out to explore the influence of different specific operators and parameter values on the aggregation results. Finally, qualitative and quantitative comparisons to the existing methods are made to demonstrate the feasibility and effectiveness of the proposed method.

### 5.1. Example

A numerical example about the determination of the best industry for investment from five possible industries (adapted on the basis of Reference [31]) is used to demonstrate the proposed MCGDM method.

To make full use of idle capital, the board of directors of a company decided to invest in a new industry. Five industries were identified as possible industries for investment after preliminary research. The five alternative industries are medical industry (*A*_1_), real estate development industry (*A*_2_), Internet industry (*A*_3_), education and training industry (*A*_4_), and manufacturing industry (*A*_5_). To select the best industry for investment, the board of directors appointed an expert panel, which consists of four different experts *E*_1_, *E*_2_, *E*_3_, and *E*_4_. The relative importance of these experts is quantified by the weight vector ***ϖ*** = [0.30, 0.22, 0.28, 0.20]. The four experts were asked to evaluate the five alternative industries on the basis of five criteria, which are the amount of capital profit (*C*_1_), the market potential (*C*_2_), the risk of capital loss (*C*_3_), the growth potential (*C*_4_), and the stability of policy (*C*_5_). The relative importance of these criteria is measured by the weight vector ***w*** = [0.20, 0.20, 0.15, 0.25, 0.20]. According to the structure of interrelationships, the five criteria are divided into two partitions ***C***_1_ = {*C*_1_, *C*_3_, *C*_5_} and ***C***_2_ = {*C*_2_, *C*_4_} and there are interrelationships among the three criteria in ***C***_1_, so do the two criteria in ***C***_2_, and ***C***_1_ and ***C***_2_ are independent of each other. To provide enough freedom in the evaluation of the values of the five criteria of each alternative industry, experts were allowed to use qROFNs. The evaluation results of the four experts are respectively listed in the following four matrices:

$$M_{1}=[\begin{array}{lllll} <0.7,\;0.2> & <0.8,\;0.2> & <0.5,\;0.4> & <0.7,\;0.1> & <0.9,\;0.2>\\ <0.8,\;0.6> & <0.7,\;0.6> & <0.5,\;0.4> & <0.5,\;0.3> & <0.7,\;0.2>\\ <0.6,\;0.5> & <0.5,\;0.4> & <0.6,\;0.5> & <0.8,\;0.5> & <0.8,\;0.3>\\ <0.7,\;0.2> & <0.6,\;0.5> & <0.5,\;0.6> & <0.6,\;0.2> & <0.6,\;0.5>\\ <0.6,\;0.4> & <0.7,\;0.5> & <0.6,\;0.5> & <0.7,\;0.4> & <0.7,\;0.3> \end{array}],$$

$$M_{2}=[\begin{array}{lllll} <0.7,\;0.1> & <0.7,\;0.3> & <0.5,\;0.3> & <0.6,\;0.1> & <0.8,\;0.2>\\ <0.8,\;0.3> & <0.7,\;0.5> & <0.7,\;0.2> & <0.9,\;0.2> & <0.6,\;0.2>\\ <0.9,\;0.2> & <0.8,\;0.3> & <0.3,\;0.5> & <0.8,\;0.4> & <0.9,\;0.2>\\ <0.6,\;0.3> & <0.7,\;0.5> & <0.6,\;0.4> & <0.8,\;0.3> & <0.7,\;0.6>\\ <0.7,\;0.3> & <0.8,\;0.4> & <0.4,\;0.3> & <0.7,\;0.2> & <0.6,\;0.3> \end{array}],$$

$$M_{3}=[\begin{array}{lllll} <0.8,\;0.2> & <0.6,\;0.2> & <0.7,\;0.3> & <0.8,\;0.1> & <0.7,\;0.1>\\ <0.8,\;0.6> & <0.7,\;0.4> & <0.8,\;0.4> & <0.7,\;0.4> & <0.6,\;0.4>\\ <0.7,\;0.2> & <0.8,\;0.3> & <0.6,\;0.2> & <0.9,\;0.2> & <0.8,\;0.2>\\ <0.4,\;0.7> & <0.9,\;0.2> & <0.5,\;0.2> & <0.5,\;0.5> & <0.9,\;0.6>\\ <0.6,\;0.4> & <0.7,\;0.3> & <0.6,\;0.3> & <0.7,\;0.3> & <0.8,\;0.5> \end{array}],$$

$$M_{4}=[\begin{array}{lllll} <0.9,\;0.3> & <0.6,\;0.2> & <0.6,\;0.3> & <0.7,\;0.3> & <0.7,\;0.2>\\ <0.6,\;0.6> & <0.7,\;0.7> & <0.6,\;0.2> & <0.6,\;0.2> & <0.6,\;0.2>\\ <0.7,\;0.1> & <0.9,\;0.4> & <0.7,\;0.4> & <0.8,\;0.3> & <0.9,\;0.4>\\ <0.7,\;0.3> & <0.5,\;0.4> & <0.5,\;0.4> & <0.9,\;0.4> & <0.6,\;0.2>\\ <0.8,\;0.4> & <0.8,\;0.5> & <0.5,\;0.2> & <0.7,\;0.4> & <0.8,\;0.3> \end{array}]$$

With the known conditions above, the determination of the best industry for investment can be carried out leveraging the proposed MCGDM method. Its process consists of the following eight steps:

1. Normalise the q-rung orthopair fuzzy decision matrices ***M***_*h*_ (*h* = 1, 2, 3, 4). Among the five criteria, the amount of capital profit (*C*_1_), the market potential (*C*_2_), the growth potential (*C*_4_), and the stability of policy (*C*_5_) are four benefit criteria and the risk of capital loss (*C*_3_) is a cost criterion. Based on this, the four matrices ***M***_*h*_ are normalised according to Eq (51) and four normalised matrices are obtained as follows:
  $$M_{N,1}={\left[Q_{1,i,j}\right]}_{5\times 5}=\left[\begin{array}{lllll} <0.7,\;0.2> & <0.8,\;0.2> & <0.4,\;0.5> & <0.7,\;0.1> & <0.9,\;0.2>\\ <0.8,\;0.6> & <0.7,\;0.6> & <0.4,\;0.5> & <0.5,\;0.3> & <0.7,\;0.2>\\ <0.6,\;0.5> & <0.5,\;0.4> & <0.5,\;0.6> & <0.8,\;0.5> & <0.8,\;0.3>\\ <0.7,\;0.2> & <0.6,\;0.5> & <0.6,\;0.5> & <0.6,\;0.2> & <0.6,\;0.5>\\ <0.6,\;0.4> & <0.7,\;0.5> & <0.5,\;0.6> & <0.7,\;0.4> & <0.7,\;0.3> \end{array}\right],$$
  $$M_{N,2}={\left[Q_{2,i,j}\right]}_{5\times 5}=\left[\begin{array}{lllll} <0.7,\;0.1> & <0.7,\;0.3> & <0.3,\;0.5> & <0.6,\;0.1> & <0.8,\;0.2>\\ <0.8,\;0.3> & <0.7,\;0.5> & <0.2,\;0.7> & <0.9,\;0.2> & <0.6,\;0.2>\\ <0.9,\;0.2> & <0.8,\;0.3> & <0.5,\;0.3> & <0.8,\;0.4> & <0.9,\;0.2>\\ <0.6,\;0.3> & <0.7,\;0.5> & <0.4,\;0.6> & <0.8,\;0.3> & <0.7,\;0.6>\\ <0.7,\;0.3> & <0.8,\;0.4> & <0.3,\;0.4> & <0.7,\;0.2> & <0.6,\;0.3> \end{array}\right],$$
  $$M_{N,3}={\left[Q_{3,i,j}\right]}_{5\times 5}=\left[\begin{array}{lllll} <0.8,\;0.2> & <0.6,\;0.2> & <0.3,\;0.7> & <0.8,\;0.1> & <0.7,\;0.1>\\ <0.8,\;0.6> & <0.7,\;0.4> & <0.4,\;0.8> & <0.7,\;0.4> & <0.6,\;0.4>\\ <0.7,\;0.2> & <0.8,\;0.3> & <0.2,\;0.6> & <0.9,\;0.2> & <0.8,\;0.2>\\ <0.4,\;0.7> & <0.9,\;0.2> & <0.2,\;0.5> & <0.5,\;0.5> & <0.9,\;0.6>\\ <0.6,\;0.4> & <0.7,\;0.3> & <0.3,\;0.6> & <0.7,\;0.3> & <0.8,\;0.5> \end{array}\right],$$
  $$M_{N,4}={\left[Q_{4,i,j}\right]}_{5\times 5}=\left[\begin{array}{lllll} <0.9,\;0.3> & <0.6,\;0.2> & <0.3,\;0.6> & <0.7,\;0.3> & <0.7,\;0.2>\\ <0.6,\;0.6> & <0.7,\;0.7> & <0.2,\;0.6> & <0.6,\;0.2> & <0.6,\;0.2>\\ <0.7,\;0.1> & <0.9,\;0.4> & <0.4,\;0.7> & <0.8,\;0.3> & <0.9,\;0.4>\\ <0.7,\;0.3> & <0.5,\;0.4> & <0.4,\;0.5> & <0.9,\;0.4> & <0.6,\;0.2>\\ <0.8,\;0.4> & <0.8,\;0.5> & <0.2,\;0.5> & <0.7,\;0.4> & <0.8,\;0.3> \end{array}\right]$$
2. Calculate the power weights of *Q*_*h*,*i*,*j*_. According to Eq (52), the power weights of *Q*_*h*,*i*,*j*_ are computed and the computed results are listed in the following matrices:
  $${\left[W_{1,i,j}\right]}_{5\times 5}=\left[\begin{array}{lllll} 0.3023 & 0.2917 & 0.2995 & 0.3064 & 0.2946\\ 0.3095 & 0.3063 & 0.2942 & 0.3006 & 0.3007\\ 0.2805 & 0.2754 & 0.3089 & 0.2954 & 0.3044\\ 0.3095 & 0.3128 & 0.2913 & 0.3013 & 0.3106\\ 0.3033 & 0.3007 & 0.2903 & 0.3028 & 0.3080 \end{array}\right],\;{\left[W_{2,i,j}\right]}_{5\times 5}=\left[\begin{array}{lllll} 0.2183 & 0.2209 & 0.2212 & 0.2195 & 0.2248\\ 0.2056 & 0.2246 & 0.2258 & 0.2093 & 0.2256\\ 0.2212 & 0.2309 & 0.2080 & 0.2262 & 0.2197\\ 0.2332 & 0.2277 & 0.2284 & 0.2303 & 0.2278\\ 0.2200 & 0.2240 & 0.2217 & 0.2127 & 0.2189 \end{array}\right],$$
  $${\left[W_{3,i,j}\right]}_{5\times 5}=\left[\begin{array}{lllll} 0.2856 & 0.2843 & 0.2750 & 0.2793 & 0.2795\\ 0.2889 & 0.2736 & 0.2746 & 0.2850 & 0.2685\\ 0.2936 & 0.2939 & 0.2776 & 0.2736 & 0.2807\\ 0.2457 & 0.2579 & 0.2719 & 0.2682 & 0.2748\\ 0.2831 & 0.2741 & 0.2855 & 0.2826 & 0.2718 \end{array}\right],\;{\left[W_{4,i,j}\right]}_{5\times 5}=\left[\begin{array}{lllll} 0.1938 & 0.2031 & 0.2043 & 0.1948 & 0.2011\\ 0.1960 & 0.1954 & 0.2053 & 0.2051 & 0.2051\\ 0.2047 & 0.1998 & 0.2055 & 0.2048 & 0.1951\\ 0.2116 & 0.2016 & 0.2084 & 0.2001 & 0.1868\\ 0.1936 & 0.2012 & 0.2026 & 0.2019 & 0.2013 \end{array}\right],$$
3. Calculate the collective values of *Q*_*h*,*i*,*j*_. Taking the normalised q-rung orthopair fuzzy decision matrices ***M***_**N**,*h*_ and the power weight matrices [*W*_*h*,*i*,*j*_]_5×5_ as input, the collective values of *Q*_*h*,*i*,*j*_ can be computed according to Eq (53). Without loss of generality (It is worth nothing that the qROFWAHPPMM operator (see Eq (39)) will reduce to the qROFWAAPPMM operator (see Eq (35)) when *λ* = 1 and will reduce to the qROFWAEPPMM operator (see Eq (36)) when *λ* = 2), the qROFWAHPPMM operator in Eq (39) (When adapting this operator, *q* = 3, *λ* = 3, and **Δ** = (*δ*_1_, *δ*_2_, *δ*_3_, *δ*_4_) = (1, 0, 0, 0)) is used in Eq (53) to complete the computation. The computed results are listed in the following matrix:
  $$M_{N}={\left[Q_{i,j}\right]}_{5\times 5}=\left[\begin{array}{lllll} <0.7825,\;0.1861> & <0.6935,\;0.2188> & <0.3361,\;0.5722> & <0.7146,\;0.1240> & <0.8000,\;0.1648>\\ <0.7706,\;0.5248> & <0.7000,\;0.5351> & <0.3407,\;0.6445> & <0.7046,\;0.2758> & <0.6336,\;0.2414>\\ <0.7429,\;0.2259> & <0.7752,\;0.3443> & <0.4293,\;0.5426> & <0.8338,\;0.3349> & <0.8492,\;0.2595>\\ <0.6250,\;0.3312> & <0.7244,\;0.3799> & <0.4503,\;0.5218> & <0.7249,\;0.3241> & <0.7416,\;0.4673>\\ <0.6720,\;0.3757> & <0.7472,\;0.4148> & <0.3704,\;0.5306> & <0.7000,\;0.3189> & <0.7360,\;0.3457> \end{array}\right]$$
4. Calculate the power weights of *Q*_*i*,*j*_. According to Eq (54), the power weights of *Q*_*i*,*j*_ are computed and the computed results are listed in the following matrix:
  $${\left[W_{i,j}\right]}_{5\times 5}=\left[\begin{array}{lllll} 0.2086 & 0.2078 & 0.1192 & 0.2577 & 0.2067\\ 0.2038 & 0.2062 & 0.1337 & 0.2549 & 0.2014\\ 0.2035 & 0.2073 & 0.1280 & 0.2574 & 0.2038\\ 0.2031 & 0.2052 & 0.1384 & 0.2534 & 0.1999\\ 0.2063 & 0.2024 & 0.1302 & 0.2562 & 0.2049 \end{array}\right]$$
5. Calculate the collective values of *Q*_*i*,*j*_. On the basis of the two partitions ***C***_1_ = {*C*_1_, *C*_3_, *C*_5_} and ***C***_2_ = {*C*_2_, *C*_4_}, the matrix ***M***_**N**_ is divided into ***S***_*i*,1_ = {*Q*_*i*,1_, *Q*_*i*,3_, *Q*_*i*,5_} and ***S***_*i*,2_ = {*Q*_*i*,2_, *Q*_*i*,4_} (*i* = 1, 2, 3, 4, 5). Taking them and the power weight matrix [*W*_*i*,*j*_]_5×5_ as input, the collective values of *Q*_*i*,*j*_ can be computed according to Eq (55). Since the qROFWAHPPMM operator in Eq (39) has been used in the third step, this operator (When adapting the operator, *q* = 3, *λ* = 3, and **Δ** = (**Δ**_1_, **Δ**_2_) = ((*δ*_1_, *δ*_2_, *δ*_3_), (*δ*_1_, *δ*_2_)) = ((1, 2, 3), (1, 2))) is also leveraged in this step to complete the computation. The computed results are listed as follows:
  *Q*_1_ = <0.5453, 0.5668>, *Q*_2_ = <0.5159, 0.7220>, *Q*_3_ = <0.6024, 0.6563>, *Q*_4_ = <0.5303, 0.6991>, *Q*_5_ = <0.5278, 0.6953>
6. Calculate the scores and accuracies of *Q*_*i*_. The scores and accuracies of *Q*_*i*_ are respectively computed using the Equations in Definitions 2 and 3. The computed results are respectively listed as follows:
  *S*(*Q*_1_) = −0.0199, *S*(*Q*_2_) = −0.2390, *S*(*Q*_3_) = −0.0641, *S*(*Q*_4_) = −0.1925, *S*(*Q*_5_) = −0.1891
  *A*(*Q*_1_) = 0.3443, *A*(*Q*_2_) = 0.5137, *A*(*Q*_3_) = 0.5013, *A*(*Q*_4_) = 0.4908, *A*(*Q*_5_) = 0.4832
7. Generate a ranking of *A*_*i*_. On the basis of the *S*(*Q*_*i*_) and *A*(*Q*_*i*_), a ranking of *A*_*i*_ is generated according to the comparison rules in Definition 4. The ranking is as follow: *A*_1_ ≻ *A*_3_ ≻ *A*_5_ ≻ *A*_4_ ≻ *A*_2_
8. Determine the optimal alternative. The best industry for investment is determined as medical industry (*A*_1_).

### 5.2. Experiments

To explore the effect of using different specific operators and assigning different parameter values on the aggregation results, the following three experiments were carried out:

(1) **Experiment 1** aims to show the influence of using different specific operators on the aggregation results. In this experiment, the presented qROFWAAPPMM (see Eq (35)), qROFWAEPPMM (see Eq (36)), qROFWAHPPMM (see Eq (39)), and qROFWAFPPMM (see Eq (42)) operators are respectively used to calculate the collective values of *Q*_*h*,*i*,*j*_ and the collective values of *Q*_*i*,*j*_ in the numerical example (When calculating the collective values of *Q*_*h*,*i*,*j*_, *q* = 3, *λ* = 3, *ε* = 2, and **Δ** = (*δ*_1_, *δ*_2_, *δ*_3_, *δ*_4_) = (1, 0, 0, 0); When calculating the collective values of *Q*_*i*,*j*_, *q* = 3, *λ* = 3, *ε* = 2, and **Δ** = (**Δ**_1_, **Δ**_2_) = ((*δ*_1_, *δ*_2_, *δ*_3_), (*δ*_1_, *δ*_2_)) = ((1, 2, 3), (1, 2))). The results of the experiment are the calculated scores of *Q*_*i*_ and the generated rankings of *A*_*i*_, which are listed in Table 1. As can be seen from the table, there is slight difference among the scores of the same *Q*_*i*_ calculated by the four pairs of specific operators, and the rankings of *A*_*i*_ also indicate small difference with respect to the four pairs of specific operators. These indicate that the use of different specific operators has no obvious influence on the aggregation results.

**Table 1:** The results of Experiment 1.

Specific operator used in Eq (53)	Specific operator used in Eq (55)	Calculated scores of *Q*_*i*_					Generated ranking of *A*_*i*_
		*S*(*Q*_1_)	*S*(*Q*_2_)	*S*(*Q*_3_)	*S*(*Q*_4_)	*S*(*Q*_5_)	
qROFWAAPPMM	qROFWAAPPMM	0.0348	−0.1641	0.0305	−0.0782	−0.0964	*A*_1_ ≻ *A*_3_ ≻ *A*_4_ ≻ *A*_5_ ≻ *A*_2_
qROFWAEPPMM	qROFWAEPPMM	0.0013	−0.2096	−0.0272	−0.1477	−0.1521	*A*_1_ ≻ *A*_3_ ≻ *A*_4_ ≻ *A*_5_ ≻ *A*_2_
qROFWAHPPMM	qROFWAHPPMM	−0.0199	−0.2390	−0.0641	−0.1925	−0.1891	*A*_1_ ≻ *A*_3_ ≻ *A*_5_ ≻ *A*_4_ ≻ *A*_2_
qROFWAFPPMM	qROFWAFPPMM	0.0193	−0.1849	0.0041	−0.1103	−0.1219	*A*_1_ ≻ *A*_3_ ≻ *A*_4_ ≻ *A*_5_ ≻ *A*_2_

**Fig 1:**
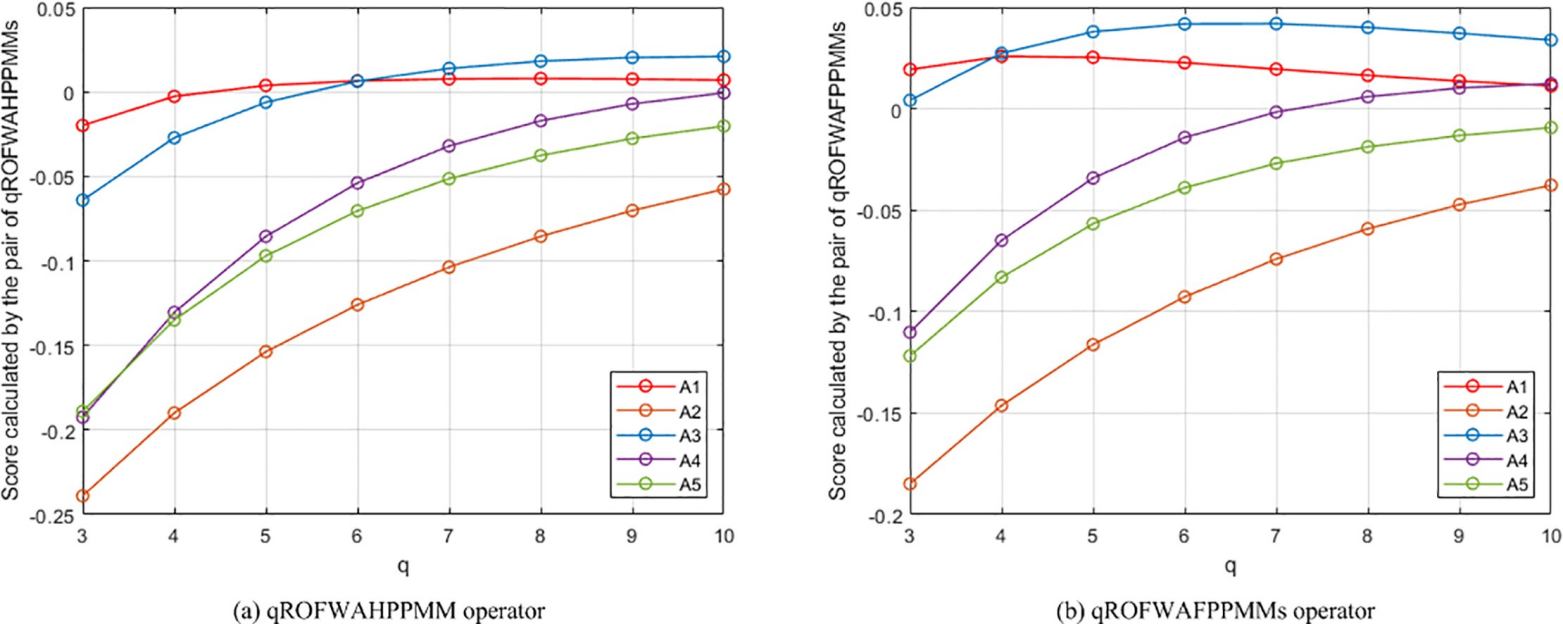
The results of Experiment 2.

**Fig 2:**
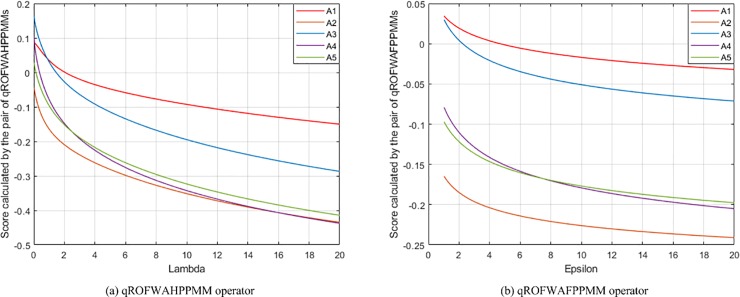
The results of Experiment 3.

(2) **Experiment 2** aims to show the influence of assigning different *q* values on the aggregation results. In this experiment, the presented qROFWAHPPMM (see Eq (39) and qROFWAFPPMM (see Eq (42)) operators (Since the qROFWAHPPMM operator is the generalisation of the qROFWAAPPMM and qROFWAEPPMM operators, they are not included in this experiment and will not be included in the subsequent experiments and comparisons for the sake of simplicity) are respectively used to calculate the collective values of *Q*_*h*,*i*,*j*_ and the collective values of *Q*_*i*,*j*_ in the numerical example (When calculating the collective values of *Q*_*h*,*i*,*j*_, *λ* = 3, *ε* = 2, and **Δ** = (*δ*_1_, *δ*_2_, *δ*_3_, *δ*_4_) = (1, 0, 0, 0); When calculating the collective values of *Q*_*i*,*j*_, *λ* = 3, *ε* = 2, and **Δ** = (**Δ**_1_, **Δ**_2_) = ((*δ*_1_, *δ*_2_, *δ*_3_), (*δ*_1_, *δ*_2_)) = ((1, 2, 3), (1, 2))). The results of the experiment are the calculated scores of *Q*_*i*_ and the generated rankings of *A*_*i*_, which are depicted in Fig 1. From the figure, it can be seen that the ranking will change as *q* changes. For the pair of qROFWAHPPMMs, the ranking is *A*_1_ ≻ *A*_3_ ≻ *A*_5_ ≻ *A*_4_ ≻ *A*_2_ when *q* = 3, changes to *A*_1_ ≻ *A*_3_ ≻ *A*_4_ ≻ *A*_5_ ≻ *A*_2_ when *q* = 4, 5, 6, and becomes *A*_3_ ≻ *A*_1_ ≻ *A*_4_ ≻ *A*_5_ ≻ *A*_2_ when *q* = 7, 8, 9, 10; The best alternative changes from *A*_1_ to *A*_3_ from *q* = 7. For the pair of qROFWAFPPMMs, the ranking is *A*_1_ ≻ *A*_3_ ≻ *A*_4_ ≻ *A*_5_ ≻ *A*_2_ when *q* = 3, changes to *A*_3_ ≻ *A*_1_ ≻ *A*_4_ ≻ *A*_5_ ≻ *A*_2_ when *q* = 4, 5, 6, 7, 8, 9, and becomes *A*_3_ ≻ *A*_4_ ≻ *A*_1_ ≻ *A*_5_ ≻ *A*_2_ when *q* = 10; The best alternative changes from *A*_1_ to *A*_3_ from *q* = 4. Based on these results, it is recommended that the smallest *q* which can meet 0 ≤ *μ*^*q*^ + *ν*^*q*^ ≤ 1 is assigned in practical applications. For example, since ***M***_**N**,3_ contains <0.9, 0.6>, *q* is assigned 3 since 0.9^2^ + 0.6^2^ > 1 and 0.9^3^ + 0.6^3^ < 1.

(3) **Experiment 3** aims to show the influence of assigning different *λ* (*ε*) values on the aggregation results. In this experiment, the presented qROFWAHPPMM (see Eq (39) and qROFWAFPPMM (see Eq (42)) operators are respectively used to calculate the collective values of *Q*_*h*,*i*,*j*_ and the collective values of *Q*_*i*,*j*_ in the numerical example (When calculating the collective values of *Q*_*h*,*i*,*j*_, *q* = 3 and **Δ** = (*δ*_1_, *δ*_2_, *δ*_3_, *δ*_4_) = (1, 0, 0, 0); When calculating the collective values of *Q*_*i*,*j*_, *q* = 3 and **Δ** = (**Δ**_1_, **Δ**_2_) = ((*δ*_1_, *δ*_2_, *δ*_3_), (*δ*_1_, *δ*_2_)) = ((1, 2, 3), (1, 2))). The results of the experiment are the calculated scores of *Q*_*i*_ and the generated rankings of *A*_*i*_, which are depicted in Fig 2. It can be seen from the figure that the scores computed by the pair of qROFWAHPPMMs (qROFWAFPPMMs) gradually decrease as *λ* (*ε*) gradually increases. Therefore, the parameter *λ* (*ε*) can be seen as a pessimistic factor for MCGDM problems. Generally, if the attitude of a decision maker is neutral, a small *λ* (*ε*) (e.g. *λ* = 1, 2, 3; *ε* = 2, 3, 4) is recommended. If the attitude is pessimistic enough, a bigger *λ* (*ε*) can be assigned when the pair of qROFWAHPPMMs (qROFWAFPPMMs) is used. Otherwise, a smaller *λ* (*ε*) is recommended.

### 5.3. Comparisons

As mentioned in the introduction, more than twenty different aggregation operators of qROFNs have been presented within academia. Representative examples are the WA and WG [26], WBM and WGBM [27], WABM [28], WPBM and WPGBM [29], WHM and WGHM [30], WHM* and WPHM [31], WMSM and WGMSM [32], WPMSM [33], WPPMSM [34], WMM and WGMM [35], WEBM [36], WE [37], and WP [38] operators. In this subsection, qualitative and quantitative comparisons between the MCGDM methods based on these operators and the proposed MCGDM method are carried out to demonstrate its feasibility and effectiveness.

#### 5.3.1. Qualitative comparison

Generally, a qualitative comparison among different MCGDM methods can be carried out by comparing their characteristics. For the twenty existing methods and the proposed method, the generality and flexibility in the aggregation of qROFNs, the capability to deal with the interrelationships among different criteria, and the capability to reduce the negative influence of the unduly high or unduly low criterion values on the aggregation results are selected as the comparison characteristics. The results of the comparison are shown in Table 2. The details of the comparison are explained as follows:

**Table 2:** The results of the qualitative comparison. Note: Heterogeneous interrelationships refer to the situation in which the criteria are divided into several parts and there are interrelationships among different criteria in each part whereas the criteria in different parts are independent of each other.

Method	Generality and flexibility	Capability to deal with the interrelationships among different criteria				Capability to reduce the negative effect
		Independent	Between any two	Among any multiple	Heterogeneous	
WA [26]	Limited	Yes	No	No	No	No
WG [26]	Limited	Yes	No	No	No	No
WBM [27]	Limited	Yes	Yes	No	No	No
WGBM [27]	Limited	Yes	Yes	No	No	No
WABM [28]	Satisfying	Yes	Yes	No	No	No
WPBM [29]	Limited	Yes	Yes	No	Yes	No
WPGBM [29]	Limited	Yes	Yes	No	Yes	No
WHM [30]	Limited	Yes	Yes	No	No	No
WGHM [30]	Limited	Yes	Yes	No	No	No
WHM* [31]	Limited	Yes	Yes	No	No	No
WPHM [31]	Limited	Yes	Yes	No	Yes	No
WMSM [32]	Limited	Yes	Yes	Yes	No	No
WGMSM [32]	Limited	Yes	Yes	Yes	No	No
WPMSM [33]	Limited	Yes	Yes	Yes	No	Yes
WPPMSM [34]	Limited	Yes	Yes	Yes	Yes	Yes
WMM [35]	Limited	Yes	Yes	Yes	No	No
WGMM [35]	Limited	Yes	Yes	Yes	No	No
WEBM [36]	Limited	Yes	Yes	No	Yes	No
WE [37]	Limited	Yes	No	No	No	No
WP [38]	Moderate	Yes	No	No	No	No
WAPPMM	Satisfying	Yes	Yes	Yes	Yes	Yes

1. Generality and flexibility: For the WP method, any one of the twenty different WP operators can be used in the aggregation. Therefore, its generality and flexibility can be seen as moderate. The generality and flexibility of the WABM method and the proposed (WAPPMM) method are desirable since the aggregations are based on the operations of any family of ATTs. The aggregations in the remaining methods are based on the operation of a specific family of ATT. Relatively, they have limited generality and flexibility.
2. When all criteria are independent of each other: It is no doubt that all of the listed methods can deal with this case.
3. When there are interrelationships between any two criteria: The WE, WP, WA, and WG methods are only suitable for the independent case. All other methods have the capability of dealing with the case in which there are interrelationships between any two criteria.
4. When there are interrelationships among any multiple criteria: The WMSM, WGMSM, WPMSM, WPPMSM, WMM, and WGMM methods and the proposed method can handle this situation because of the use of the MSM or MM operator.
5. When there are heterogeneous interrelationships among different criteria: The WPBM, WPGBM, WPHM, WPPMSM, and WEBM methods and the proposed method can deal with this case due to the combination of the partitioned average operator.
6. Capability to reduce the negative effect: The WPMSM and WPPMSM methods and the proposed method have this capability because of the combination of the PA operator.

As can be summarised from the qualitative comparison above, the proposed method has desirable generality and flexibility at both aggregating the q-rung orthopair fuzzy information and dealing with the interrelationships of criteria, and has the capability to reduce the negative influence caused by the deviation of some criterion values.

#### 5.3.2. Quantitative comparison

**Fig 3:**
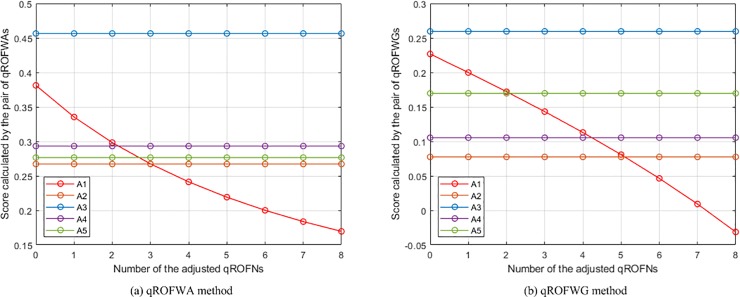
The changes of the places of *A*_1_ in the rankings of the WA and WG methods.

**Fig 4:**
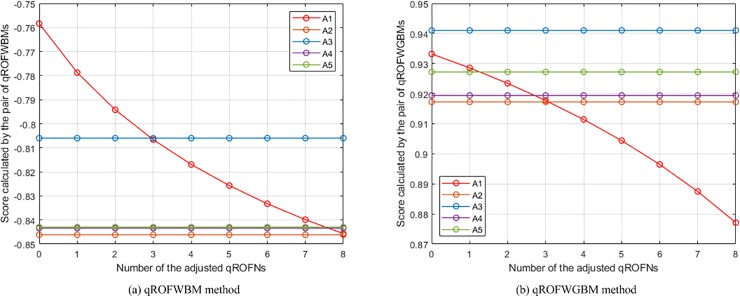
The changes of the places of *A*_1_ in the rankings of the WBM and WGBM methods.

**Fig 5:**
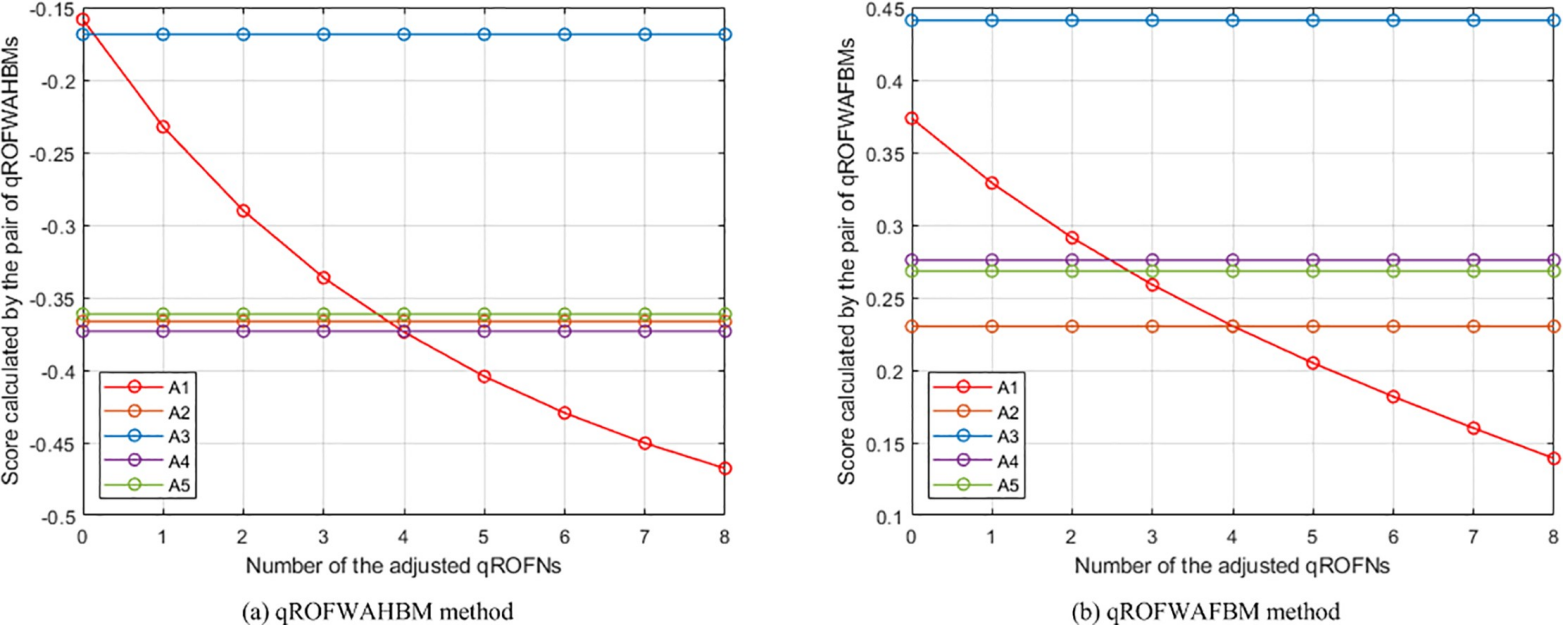
The changes of the places of *A*_1_ in the rankings of the WABM method.

**Fig 6:**
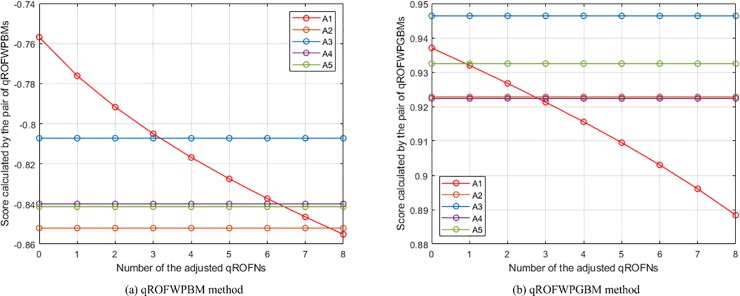
The changes of the places of *A*_1_ in the rankings of the WPBM and WPGBM methods.

**Fig 7:**
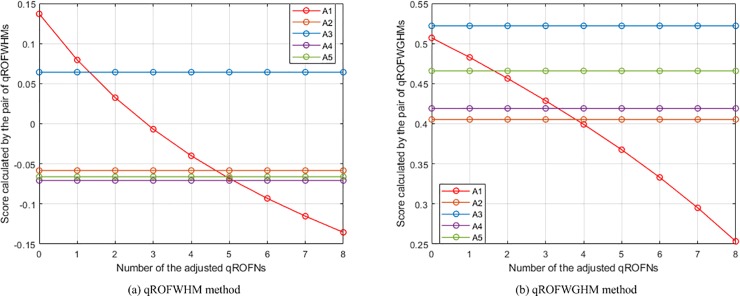
The changes of the places of *A*_1_ in the rankings of the WHM and WGHM methods.

**Fig 8:**
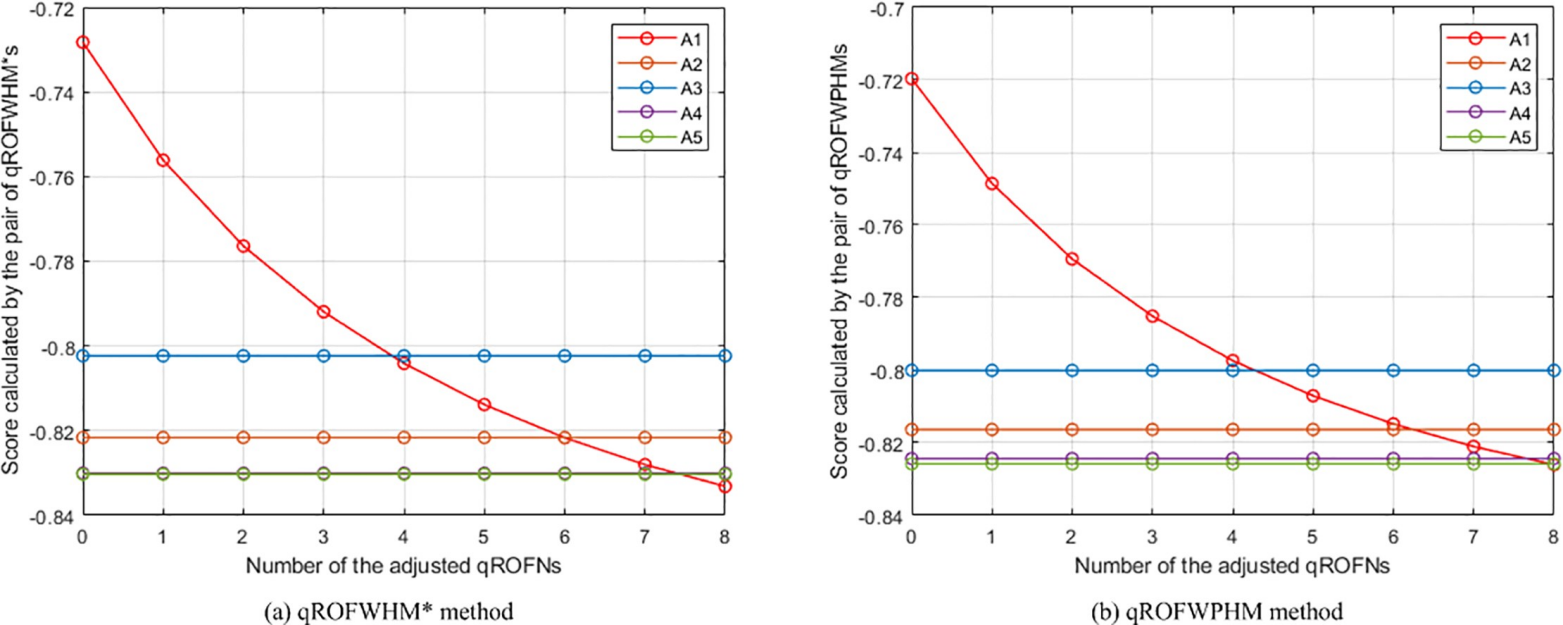
The changes of the places of *A*_1_ in the rankings of the WHM* and WPHM methods.

**Fig 9:**
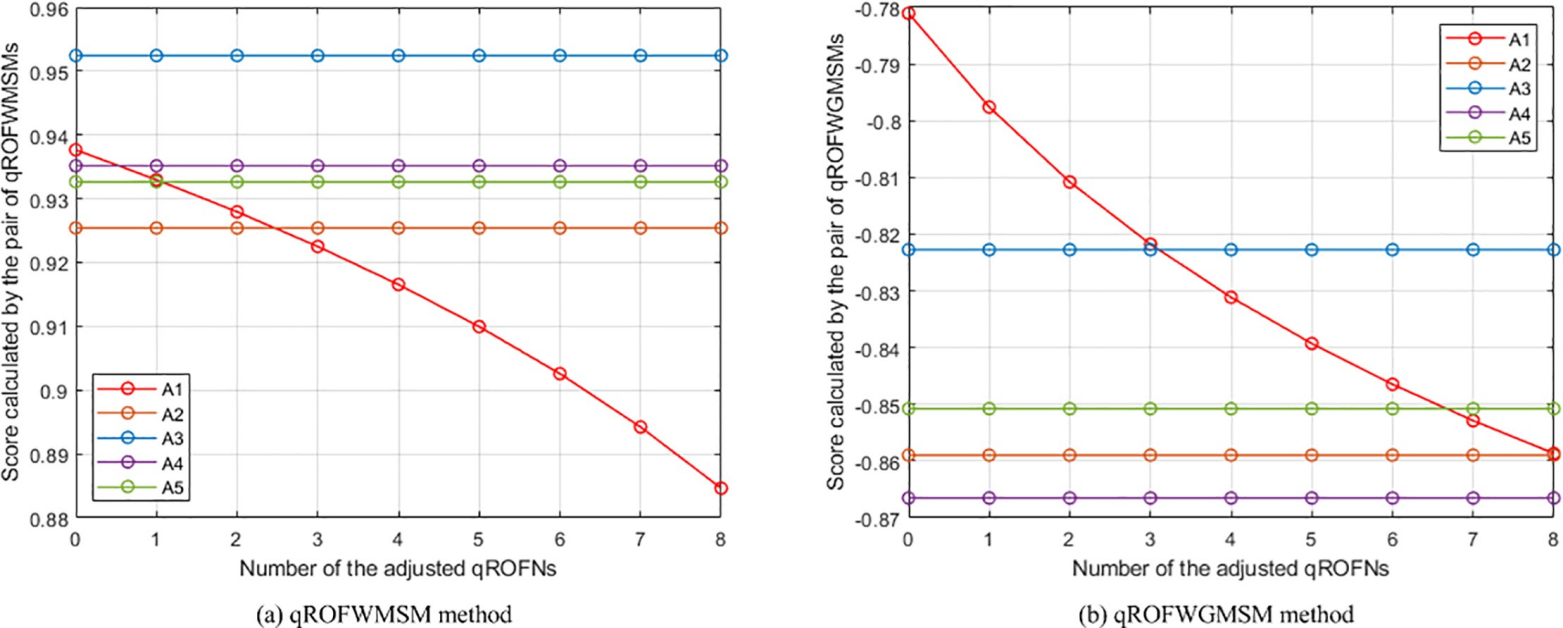
The changes of the places of *A*_1_ in the rankings of the WMSM and WGMSM methods.

**Fig 10:**
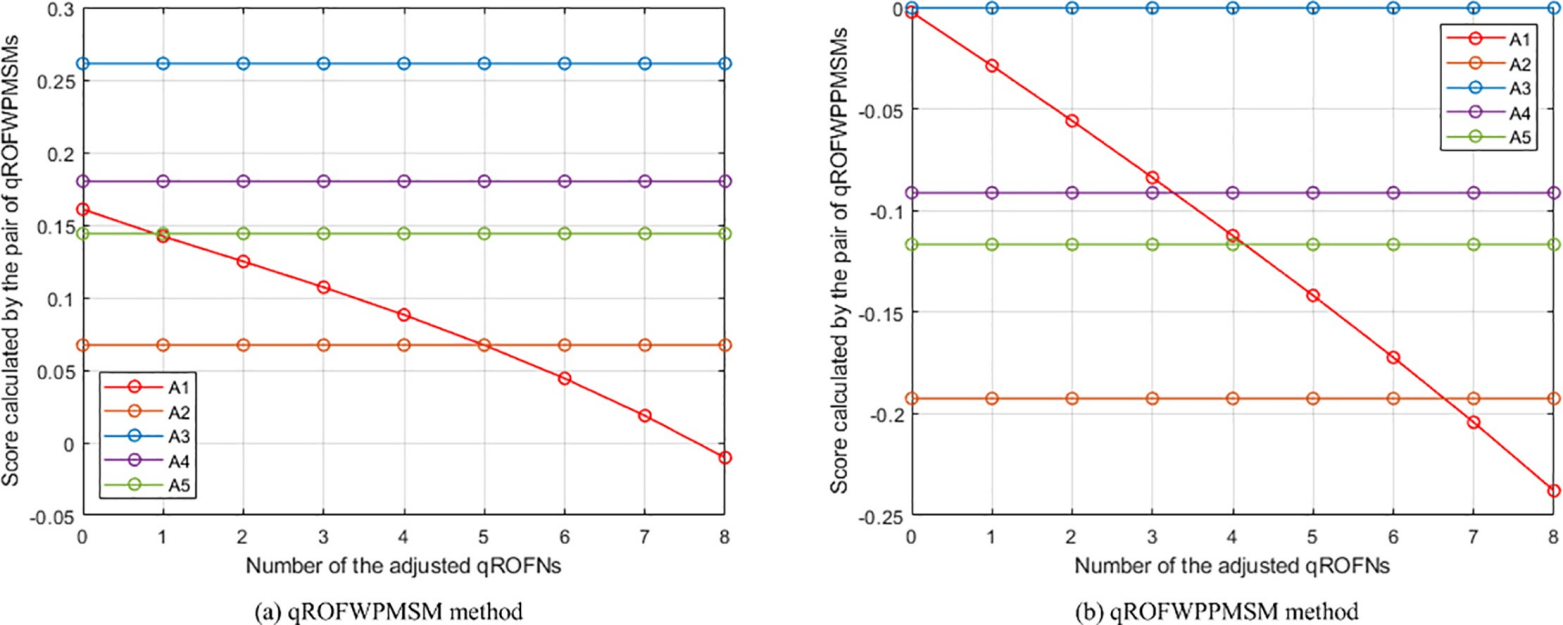
The changes of the places of *A*_1_ in the rankings of the WPMSM and WPPMSM methods.

**Fig 11:**
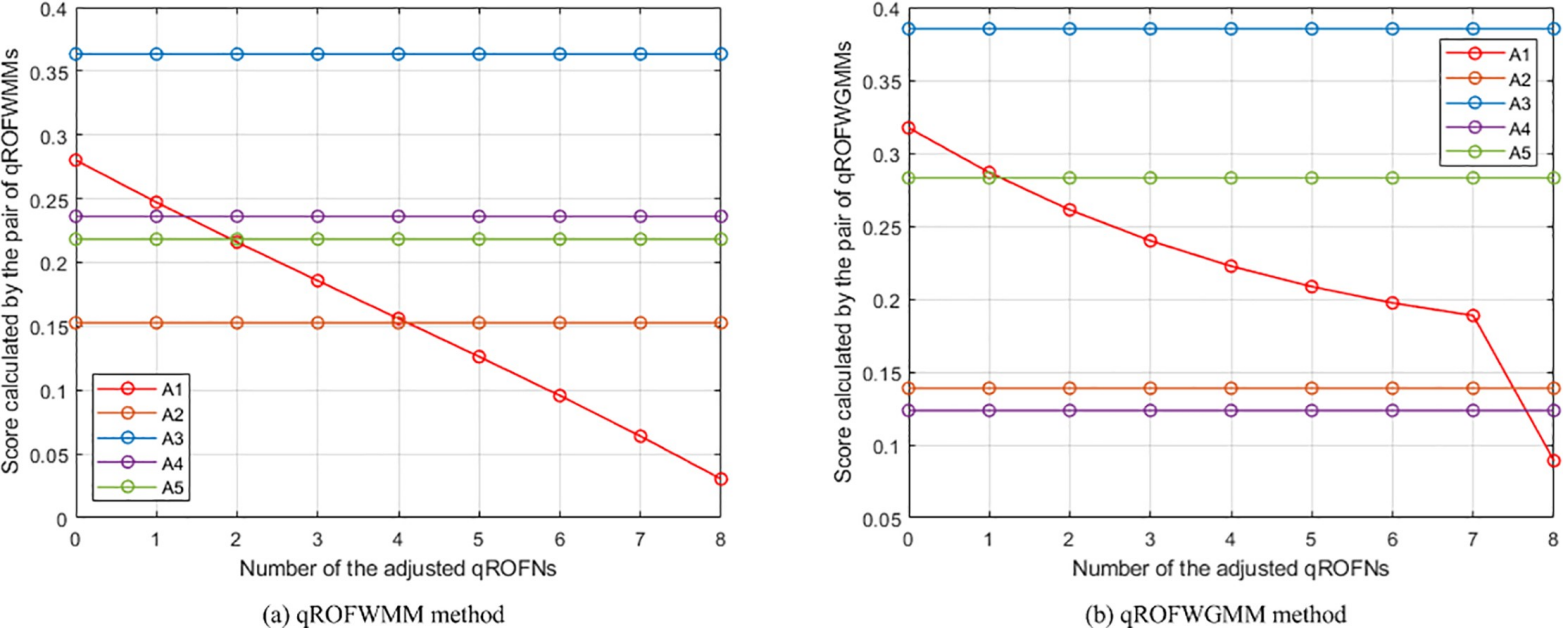
The changes of the places of *A*_1_ in the rankings of the WMM and WGMM methods.

**Fig 12:**
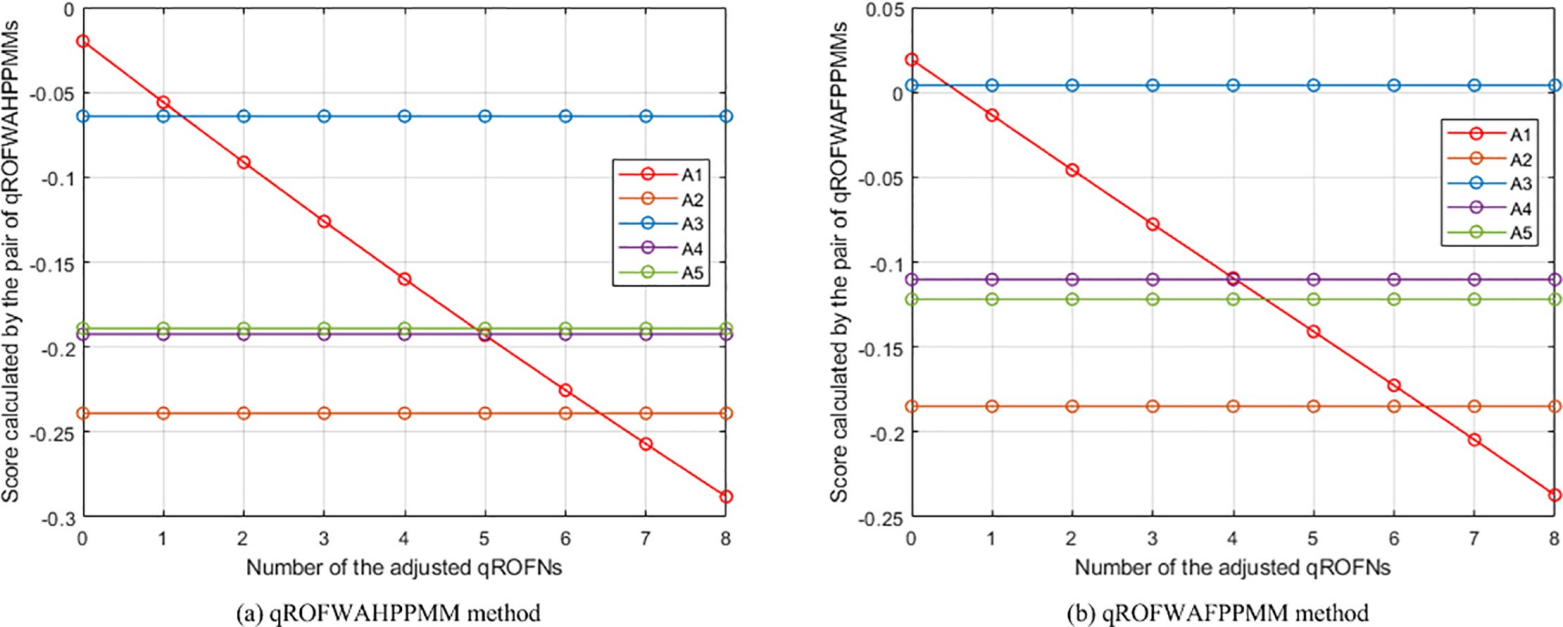
The changes of the places of *A*_1_ in the rankings of the WAPPMM method.

In general, a quantitative comparison among different MCGDM methods can be carried out using the same numerical example. Here the numerical example in subsection 5.1 is used to quantitatively compare the proposed method to the WA, WG, WBM, WGBM, WABM, WPBM, WPGBM, WHM, WGHM, WHM*, WPHM, WMSM, WGMSM, WPMSM, WPPMSM, WMM, and WGMM methods (Please note that the WEBM, WE, and WP operators were not included in the quantitative comparison because the WEBM method was pre-sented to handle the situation with unknown criterion weights, the WE method was presented to deal with the case where the values of criteria are expressed by a fuzzy number in Zadeh’s FS and the values of weights are described by qROFNs, the WP method was presented to control the uncertainty of the evaluation results of some experts, and the proposed method does not consider these characteristics). In this comparison, the qROFNs *Q*_*h*,1,4_ and *Q*_*h*,1,5_ (*h* = 1, 2, 3, 4) in ***M***_*h*_ were constantly adjusted from high score to low score according to Table 3. It is easy to guess that such adjustments will lower the ranking of *A*_1_, because the score becomes lower and lower. To confirm this conjecture, the methods were implemented to generate the changes of the places of *A*_1_ in their respective rankings. The specific generation process is as follows:

1. Calculate the collective values of *Q*_*h*,*i*,*j*_. A specific operator presented in each method, as listed in Table 4, was leveraged to compute the collective values of *Q*_*h*,*i*,*j*_ in the numerical example on the basis of certain *Q*_*h*,1,4_ and *Q*_*h*,1,5_ and certain parameters (whose values are also listed in Table 4).
2. Calculate the collective values of *Q*_*i*,*j*_. For each method, the same specific operator used in the calculation of the collective values of *Q*_*h*,*i*,*j*_, as listed in Table 4, was leveraged to compute the collective values of *Q*_*i*,*j*_ on the basis of the computed collective values of *Q*_*h*,*i*,*j*_ of each group of *Q*_*h*,1,4_ and *Q*_*h*,1,5_ and certain parameters (whose values are also listed in Table 4).
3. Calculate the scores of *Q*_*i*_. For each method, the scores of *Q*_*i*_ of each group of *Q*_*h*,1,4_ and *Q*_*h*,1,5_ were computed according to the calculated collective values of *Q*_*i*,*j*_ of this group of *Q*_*h*,1,4_ and *Q*_*h*,1,5_ and the Equation in Definition 2.
4. Generate the changes of the places of *A*_1_. According to the calculated scores of *Q*_*i*_ of each group of *Q*_*h*,1,4_ and *Q*_*h*,1,5_ of each method (The calculated scores of *Q*_1_ of the nine groups of *Q*_*h*,1,4_ and *Q*_*h*,1,5_ of each method are listed in Table 5), the changes of the places of *A*_1_ in the rankings of all comparison methods were generated and are depicted in Figs 3−12.

**Table 3:** The values of the adjusted qROFNs in the quantitative comparison.

No.	*Q*_1,1,4_	*Q*_1,1,5_	*Q*_2,1,4_	*Q*_2,1,5_	*Q*_3,1,4_	*Q*_3,1,5_	*Q*_4,1,4_	*Q*_4,1,5_
0	<0.70, 0.10>	<0.90, 0.20>	<0.60, 0.10>	<0.80, 0.20>	<0.80, 0.10>	<0.70, 0.10>	<0.70, 0.30>	<0.70, 0.20>
1	<0.65, 0.15>	<0.85, 0.25>	<0.55, 0.15>	<0.75, 0.25>	<0.75, 0.15>	<0.65, 0.15>	<0.65, 0.35>	<0.65, 0.25>
2	<0.60, 0.20>	<0.80, 0.30>	<0.50, 0.20>	<0.70, 0.30>	<0.70, 0.20>	<0.60, 0.20>	<0.60, 0.40>	<0.60, 0.30>
3	<0.55, 0.25>	<0.75, 0.35>	<0.45, 0.25>	<0.65, 0.35>	<0.65, 0.25>	<0.55, 0.25>	<0.55, 0.45>	<0.55, 0.35>
4	<0.50, 0.30>	<0.70, 0.40>	<0.40, 0.30>	<0.60, 0.40>	<0.60, 0.30>	<0.50, 0.30>	<0.50, 0.50>	<0.50, 0.40>
5	<0.45, 0.35>	<0.65, 0.45>	<0.35, 0.35>	<0.55, 0.45>	<0.55, 0.35>	<0.45, 0.35>	<0.45, 0.55>	<0.45, 0.45>
6	<0.40, 0.40>	<0.60, 0.50>	<0.30, 0.40>	<0.50, 0.50>	<0.50, 0.40>	<0.40, 0.40>	<0.40, 0.60>	<0.40, 0.50>
7	<0.35, 0.45>	<0.55, 0.55>	<0.25, 0.45>	<0.45, 0.55>	<0.45, 0.45>	<0.35, 0.45>	<0.35, 0.65>	<0.35, 0.55>
8	<0.30, 0.50>	<0.50, 0.60>	<0.20, 0.50>	<0.40, 0.60>	<0.40, 0.50>	<0.30, 0.50>	<0.30, 0.70>	<0.30, 0.60>

**Table 4:** The specific operators and their parameter values for calculating the collective values of *Q*_h_,*i*_,*j*_ and *Q*_i_,_*j*_.

Method	Operator leveraged to calculate the collective values of *Q*_*h*,*i*,*j*_	Operator leveraged to calculate the collective values of *Q*_*i*,*j*_	Values of parameters when calculating the collective values of *Q*_*h*,*i*,*j*_	Values of parameters when calculating the collective values of *Q*_*i*,*j*_
WA [26]	qROFWA	qROFWA	*q* = 3	*q* = 3
WG [26]	qROFWG	qROFWG	*q* = 3	*q* = 3
WBM [27]	qROFWBM	qROFWBM	*q* = 3; *δ*_1_ = 1; *δ*_2_ = 0	*q* = 3; *δ*_1_ = 1; *δ*_2_ = 2
WGBM [27]	qROFWGBM	qROFWGBM	*q* = 3; *δ*_1_ = 1; *δ*_2_ = 0	*q* = 3; *δ*_1_ = 1; *δ*_2_ = 2
WABM [28]	qROFWAHBM	qROFWAHBM	*q* = 3; *δ*_1_ = 1; *δ*_2_ = 0; *λ* = 3	*q* = 3; *δ*_1_ = 1; *δ*_2_ = 2; *λ* = 3
WABM [28]	qROFWAFBM	qROFWAFBM	*q* = 3; *δ*_1_ = 1; *δ*_2_ = 0; *ε* = 2	*q* = 3; *δ*_1_ = 1; *δ*_2_ = 2; *ε* = 2
WPBM [29]	qROFWPBM	qROFWPBM	*q* = 3; *δ*_1_ = 1; *δ*_2_ = 0	*q* = 3; ((*δ*_1_, *δ*_2_), (*δ*_1_, *δ*_2_)) = ((1, 2), (1, 2))
WPGBM [29]	qROFWPGBM	qROFWPGBM	*q* = 3; *δ*_1_ = 1; *δ*_2_ = 0	*q* = 3; ((*δ*_1_, *δ*_2_), (*δ*_1_, *δ*_2_)) = ((1, 2), (1, 2))
WHM [30]	qROFWHM	qROFWHM	*q* = 3; *δ*_1_ = 1; *δ*_2_ = 0	*q* = 3; *δ*_1_ = 1; *δ*_2_ = 2
WGHM [30]	qROFWGHM	qROFWGHM	*q* = 3; *δ*_1_ = 1; *δ*_2_ = 0	*q* = 3; *δ*_1_ = 1; *δ*_2_ = 2
WHM* [31]	qROFWHM*	qROFWHM*	*q* = 3; *δ*_1_ = 1; *δ*_2_ = 0	*q* = 3; *δ*_1_ = 1; *δ*_2_ = 2
WPHM [31]	qROFWPHM	qROFWPHM	*q* = 3; *δ*_1_ = 1; *δ*_2_ = 0	*q* = 3; ((*δ*_1_, *δ*_2_), (*δ*_1_, *δ*_2_)) = ((1, 2), (1, 2))
WMSM [32]	qROFWMSM	qROFWMSM	*q* = 3; *δ* = 1	*q* = 3; *δ* = 5
WGMSM [32]	qROFWGMSM	qROFWGMSM	*q* = 3; *δ* = 1	*q* = 3; *δ* = 5
WPMSM [33]	qROFWPMSM	qROFWPMSM	*q* = 3; *δ* = 1	*q* = 3; *δ* = 5
WPPMSM [34]	qROFWPPMSM	qROFWPPMSM	*q* = 3; *δ* = 1	*q* = 3; ((*δ*_1_), (*δ*_1_)) = ((3), (2))
WMM [35]	qROFWMM	qROFWMM	*q* = 3; (*δ*_1_, *δ*_2_, *δ*_3_, *δ*_4_) = (1, 0, 0, 0)	*q* = 3; (*δ*_1_, *δ*_2_, *δ*_3_, *δ*_4_, *δ*_5_) = (1, 2, 3, 4, 5)
WGMM [35]	qROFWGMM	qROFWGMM	*q* = 3; (*δ*_1_, *δ*_2_, *δ*_3_, *δ*_4_) = (1, 0, 0, 0)	*q* = 3; (*δ*_1_, *δ*_2_, *δ*_3_, *δ*_4_, *δ*_5_) = (1, 2, 3, 4, 5)
WAPPMM	qROFWAHPPMM	qROFWAHPPMM	*q* = 3; (*δ*_1_,*δ*_2_,*δ*_3_,*δ*_4_) = (1, 0, 0, 0); *λ* = 3	*q* = 3; ((*δ*_1_,*δ*_2_,*δ*_3_), (*δ*_1_,*δ*_2_)) = ((1, 2, 3), (1, 2)); *λ* = 3
WAPPMM	qROFWAFPPMM	qROFWAFPPMM	*q* = 3; (*δ*_1_,*δ*_2_,*δ*_3_,*δ*_4_) = (1, 0, 0, 0); *ε* = 2	*q* = 3; ((*δ*_1_,*δ*_2_,*δ*_3_), (*δ*_1_,*δ*_2_)) = ((1, 2, 3), (1, 2)); *ε* = 2

**Table 5:** The calculated scores of *Q*_1_ of the nine groups of *Q*_h,1,4_ and *Q*_h,1,5_ of all comparison methods.

Method	Used specific operators	Calculated scores of *Q*_1_ of the nine groups of *Q*_*h*,1,4_ and *Q*_*h*,1,5_ in Table 3									Ranking of *A*_1_
		0	1	2	3	4	5	6	7	8	
WA [26]	qROFWAs	0.3812	0.3354	0.2983	0.2674	0.2413	0.2191	0.2001	0.1837	0.1695	Fig 3(A)
WG [26]	qROFWGs	0.2268	0.1998	0.1720	0.1432	0.1129	0.0808	0.0464	0.0092	−0.0313	Fig 3(B)
WBM [27]	qROFWBMs	−0.7584	−0.7788	−0.7942	−0.8066	−0.8169	−0.8257	−0.8333	−0.8399	−0.8457	Fig 4(A)
WGBM [27]	qROFWGBMs	0.9332	0.9286	0.9234	0.9177	0.9114	0.9044	0.8965	0.8875	0.8771	Fig 4(B)
WABM [28]	qROFWAHBMs	−0.1584	−0.2324	−0.2902	−0.3364	−0.3738	−0.4043	−0.4295	−0.4503	−0.4677	Fig 5(A)
WABM [28]	qROFWAFBMs	0.3735	0.3289	0.2912	0.2587	0.2303	0.2049	0.1817	0.1600	0.1391	Fig 5(B)
WPBM [29]	qROFWPBMs	−0.7568	−0.7761	−0.7917	−0.8050	−0.8168	−0.8275	−0.8373	−0.8465	−0.8552	Fig 6(A)
WPGBM [29]	qROFWPGBMs	0.9371	0.9320	0.9267	0.9213	0.9156	0.9095	0.9030	0.8961	0.8884	Fig 6(B)
WHM [30]	qROFWHMs	0.1370	0.0795	0.0325	−0.0068	−0.0400	−0.0683	−0.0931	−0.1153	−0.1354	Fig 7(A)
WGHM [30]	qROFWGHMs	0.5070	0.4827	0.4565	0.4287	0.3992	0.3676	0.3331	0.2951	0.2534	Fig 7(B)
WHM* [31]	qROFWHM*s	−0.7283	−0.7561	−0.7764	−0.7919	−0.8041	−0.8139	−0.8217	−0.8280	−0.8332	Fig 8(A)
WPHM [31]	qROFWPHMs	−0.7197	−0.7486	−0.7694	−0.7851	−0.7974	−0.8071	−0.8148	−0.8211	−0.8261	Fig 8(B)
WMSM [32]	qROFWMSMs	0.9377	0.9330	0.9279	0.9225	0.9165	0.9100	0.9026	0.8942	0.8846	Fig 9(A)
WGMSM [32]	qROFWGMSMs	−0.7811	−0.7976	−0.8108	−0.8218	−0.8311	−0.8393	−0.8465	−0.8529	−0.8587	Fig 9(B)
WPMSM [33]	qROFWPMSMs	0.1608	0.1421	0.1248	0.1071	0.0881	0.0673	0.0443	0.0186	−0.0103	Fig 10(A)
WPPMSM [34]	qROFWPPMSMs	−0.0024	−0.0288	−0.0559	−0.0838	−0.1124	−0.1418	−0.1723	−0.2043	−0.2380	Fig 10(B)
WMM [35]	qROFWMMs	0.2802	0.2472	0.2159	0.1857	0.1560	0.1262	0.0957	0.0639	0.0303	Fig 11(A)
WGMM [35]	qROFWGMMs	0.3174	0.2867	0.2611	0.2399	0.2225	0.2084	0.1973	0.1886	0.0891	Fig 11(B)
WAPPMM	qROFWAHPPMMs	−0.0199	−0.0559	−0.0913	−0.1261	−0.1600	−0.1931	−0.2255	−0.2571	−0.2881	Fig 12(A)
WAPPMM	qROFWAFPPMMs	0.0193	−0.0135	−0.0457	−0.0777	−0.1094	−0.1409	−0.1726	−0.2046	−0.2372	Fig 12(B)

From the ten figures, it can be seen that the results of all comparison methods are consistent with the conjecture. This verifies the feasibility and effectiveness of the proposed method. In addition, as can be found from the figures of WA, WBM, WAHBM, WAFBM, WPBM, WHM, WHM*, WPHM, WMSM, WPMSM, WPPMSM, WMM, WAHPPMM, and WAFPPMM (In addition to these operators, the remaining comparison operators belong to geometric operators. Because the presented operators do not be-long to geometric operators, the geometric operators are not included in the discussion of the quantitative comparison), the rankings of *A*_1_ generated by the WA, WAFBM, WMSM, WPMSM, WPPMSM, and WMM methods do not start from the first, which is obviously different from the WBM, WAHBM, WPBM, WHM, WHM*, WPHM, WAHPPMM, and WAFPPMM methods. From the given data in the numerical example it is not difficult to determine that *A*_1_ is the best alternative. Therefore, the results of the latter group of methods are more reasonable than that of the former group of methods.

Among the methods in the latter group, the WBM, WAHBM, WHM, and WHM* methods can deal with the case in which all criteria are independent of each other or there are interrelationships between any two criteria, and the WPBM and WPHM methods are applicable for the situation where the criteria are divided into several partitions and there are interrelationships between any two criteria in each partition whereas the criteria in different partitions are independent of each other. These six methods are different with the proposed (WAHPPMM, WAFPPMM) method in characteristics.

Except the six methods, it is also of necessity to compare the results of the WMM method and the proposed (WAHPPMM, WAFPPMM) method. The difference in characteristics between them is that the proposed method can provide desirable generality and flexibility, deal with the heterogeneous relationships among criteria, and reduce the negative effect of the deviation of criterion values. The intuitive manifestation of such difference is that their results are significantly different. As can be seen from Fig 11(A) and Fig 12(A), the ranking generated by the WMM method is *A*_3_ ≻ *A*_1_ ≻ *A*_4_ ≻ *A*_5_ ≻ *A*_2_ at 0, changes to *A*_3_ ≻ *A*_4_ ≻ *A*_5_ ≻ *A*_1_ ≻ *A*_2_ at 2, and becomes *A*_3_ ≻ *A*_4_ ≻ *A*_5_ ≻ *A*_2_ ≻ *A*_1_ at 5; while the ranking generated by the WAHPPMM method is *A*_1_ ≻ *A*_3_ ≻ *A*_5_ ≻ *A*_4_ ≻ *A*_2_ at 0, changes to *A*_3_ ≻ *A*_1_ ≻ *A*_5_ ≻ *A*_4_ ≻ *A*_2_ at 2 and *A*_3_ ≻ *A*_5_ ≻ *A*_4_ ≻ *A*_1_ ≻ *A*_2_ at 5, and becomes *A*_3_ ≻ *A*_5_ ≻ *A*_4_ ≻ *A*_2_ ≻ *A*_1_ at 7. Hence, the rankings of the two methods are different, which is caused by the capability to handle the interrelationships among criteria. In addition, the place of *A*_1_ in the rankings generated by the WMM method descends faster than that in the rankings of the WAHPPMM method. This is because the WAHPPMM method has the capability to reduce the influence of the distortion of criterion values and the WMM method does not have such capability.

Finally, it should be pointed out that the quantitative comparison does not aim to find out the best method, but to demonstrate the feasibility and effectiveness of the proposed method and illustrate the difference of different methods. Generally, it is difficult to conclude that one MCGDM method is better than the others because each method have its specific characteristics, which determine its specific application scenario. A wider range of methods offer decision makers a greater flexibility when selecting a proper method for their specific application scenario.

## 6. Conclusion

In this paper, an Archimedean power partitioned MM operator and a weighted Archimedean power partitioned MM operator have been presented to solve the MCGDM problems based on qROFNs. The idempotency and boundedness of the Archimedean power partitioned MM operator have been proved and the four specific expressions of the two operators have been constructed leveraging the operational rules of qROFNs based on the Algebraic, Einstein, Hamacher, and Frank families of ATTs and their additive generators. On the basis of the presented weighted Archimedean power partitioned MM operator, a method for solving the MCGDM problems based on qROFNs has been proposed. The paper has also provided a numerical example coupled with a set of experiments to illustrate the working process of the proposed method and reported qualitative and quantitative comparisons to demonstrate its feasibility and effectiveness. The results of the comparisons suggest that the proposed method is general and flexible at both aggregation of criterion values and capture of criterion interrelationships, and concurrently has the capability to handle the heterogeneous interrelationships of criteria and reduce the negative effect of the biased criterion values.

Future work will focus especially on extending the presented operators from the aspect of dealing with more complex interrelationships of criteria and risk attitudes of decision makers. Further, the application of the proposed method in solving practical decision making problems, such as manufacturing process selection, part build orientation determination, medical diagnosis, and resource evaluation, will also be studied.

## Supporting information

S1 File Appendixes A, B, and C: The proofs of Theorems 1, 2, and 3.
(PDF)

S2 File The Java code of all comparison methods. https://github.com/YuchuChingQin/AOsOfqROFNsForMCGDM.
(ZIP)
