# Supplementary material for: Multi-criteria group decision making based on Archimedean power partitioned Muirhead mean operators of q-rung orthopair fuzzy numbers
Source: PLoS One. 2019 Sep 5;14(9):e0221759. doi: 10.1371/journal.pone.0221759 (PMC6728046; doi:10.1371/journal.pone.0221759)
Supplement: S1 File — Appendixes A, B, and C: The proofs of Theorems 1, 2, and 3. (PDF) [file pone.0221759.s001.pdf]

Appendix A. Proof of Theorem 1

Proof.

(1) Let

$$\xi_{p(i_k)} = \left(1+T(Q_{p(i_k)})\right) \Big/ \sum_{j=1}^{n}\left(1+T(Q_j)\right)$$

Then we have

$$\frac{1}{N}\left(\bigoplus_{k=1}^{N}\left(\frac{1}{|\mathbf{S}_k|!}\bigoplus_{p\in \mathbf{P}_{|\mathbf{S}_k|}}\bigotimes_{i_k=1}^{|\mathbf{S}_k|}\left(\frac{n\left(1+T(Q_{p(i_k)})\right)}{\sum_{j=1}^{n}\left(1+T(Q_j)\right)}Q_{p(i_k)}\right)^{\delta_{i_k}}\right)^{\frac{1}{\sum_{i_k=1}^{|\mathbf{S}_k|}\delta_{i_k}}}\right) = \frac{1}{N}\left(\bigoplus_{k=1}^{N}\left(\frac{1}{|\mathbf{S}_k|!}\bigoplus_{p\in \mathbf{P}_{|\mathbf{S}_k|}}\bigotimes_{i_k=1}^{|\mathbf{S}_k|}\left((n\xi_{p(i_k)})Q_{p(i_k)}\right)^{\delta_{i_k}}\right)^{\frac{1}{\sum_{i_k=1}^{|\mathbf{S}_k|}\delta_{i_k}}}\right)$$

According to the multiplication operation in Equation (3), we have

$$(n\xi_{p(i_k)})Q_{p(i_k)} = \left\langle g^{-1}\left((n\xi_{p(i_k)})g(\mu_{p(i_k)})\right),\ f^{-1}\left((n\xi_{p(i_k)})f(\nu_{p(i_k)})\right)\right\rangle$$

According to the power operation in Equation (4), we can obtain

$$\left((n\xi_{p(i_k)})Q_{p(i_k)}\right)^{\delta_{i_k}} = \left\langle f^{-1}\left(\delta_{i_k} f\left(g^{-1}\left((n\xi_{p(i_k)})g(\mu_{p(i_k)})\right)\right)\right),\ g^{-1}\left(\delta_{i_k} g\left(f^{-1}\left((n\xi_{p(i_k)})f(\nu_{p(i_k)})\right)\right)\right)\right\rangle$$

According to the product operation in Equation (2), we have

$$\bigotimes_{i_k=1}^{|\mathbf{S}_k|}\left((n\xi_{p(i_k)})Q_{p(i_k)}\right)^{\delta_{i_k}} = \left\langle f^{-1}\left(\sum_{i_k=1}^{|\mathbf{S}_k|}\left(\delta_{i_k} f\left(g^{-1}\left((n\xi_{p(i_k)})g(\mu_{p(i_k)})\right)\right)\right)\right),\ g^{-1}\left(\sum_{i_k=1}^{|\mathbf{S}_k|}\left(\delta_{i_k} g\left(f^{-1}\left((n\xi_{p(i_k)})f(\nu_{p(i_k)})\right)\right)\right)\right)\right\rangle$$

According to the sum operation in Equation (1), we can obtain

$$\bigoplus_{p\in \mathbf{P}_{|\mathbf{S}_k|}}\bigotimes_{i_k=1}^{|\mathbf{S}_k|}\left((n\xi_{p(i_k)})Q_{p(i_k)}\right)^{\delta_{i_k}} = \left\langle g^{-1}\left(\sum_{p\in \mathbf{P}_{|\mathbf{S}_k|}} g\left(f^{-1}\left(\sum_{i_k=1}^{|\mathbf{S}_k|}\left(\delta_{i_k} f\left(g^{-1}\left((n\xi_{p(i_k)})g(\mu_{p(i_k)})\right)\right)\right)\right)\right)\right),\right.$$
$$\left. f^{-1}\left(\sum_{p\in \mathbf{P}_{|\mathbf{S}_k|}} f\left(g^{-1}\left(\sum_{i_k=1}^{|\mathbf{S}_k|}\left(\delta_{i_k} g\left(f^{-1}\left((n\xi_{p(i_k)})f(\nu_{p(i_k)})\right)\right)\right)\right)\right)\right)\right\rangle$$

According to the multiplication operation in Equation (3), we have

$$\frac{1}{|\mathbf{S}_k|!}\bigoplus_{p\in \mathbf{P}_{|\mathbf{S}_k|}}\bigotimes_{i_k=1}^{|\mathbf{S}_k|}\left((n\xi_{p(i_k)})Q_{p(i_k)}\right)^{\delta_{i_k}} = \left\langle g^{-1}\left(\frac{1}{|\mathbf{S}_k|!}\sum_{p\in \mathbf{P}_{|\mathbf{S}_k|}} g\left(f^{-1}\left(\sum_{i_k=1}^{|\mathbf{S}_k|}\left(\delta_{i_k} f\left(g^{-1}\left((n\xi_{p(i_k)})g(\mu_{p(i_k)})\right)\right)\right)\right)\right)\right),\right.$$
$$\left. f^{-1}\left(\frac{1}{|\mathbf{S}_k|!}\sum_{p\in \mathbf{P}_{|\mathbf{S}_k|}} f\left(g^{-1}\left(\sum_{i_k=1}^{|\mathbf{S}_k|}\left(\delta_{i_k} g\left(f^{-1}\left((n\xi_{p(i_k)})f(\nu_{p(i_k)})\right)\right)\right)\right)\right)\right)\right\rangle$$

According to the power operation in Equation (4), we can obtain

$$\left(\frac{1}{|\mathbf{S}_k|!}\bigoplus_{p\in \mathbf{P}_{|\mathbf{S}_k|}}\bigotimes_{i_k=1}^{|\mathbf{S}_k|}\left((n\xi_{p(i_k)})Q_{p(i_k)}\right)^{\delta_{i_k}}\right)^{\frac{1}{\sum_{i_k=1}^{|\mathbf{S}_k|}\delta_{i_k}}} = \left\langle f^{-1}\left(\frac{1}{\sum_{i_k=1}^{|\mathbf{S}_k|}\delta_{i_k}} f\left(g^{-1}\left(\frac{1}{|\mathbf{S}_k|!}\sum_{p\in \mathbf{P}_{|\mathbf{S}_k|}} g\left(f^{-1}\left(\sum_{i_k=1}^{|\mathbf{S}_k|}\left(\delta_{i_k} f\left(g^{-1}\left((n\xi_{p(i_k)})g(\mu_{p(i_k)})\right)\right)\right)\right)\right)\right)\right)\right),\right.$$
$$\left. g^{-1}\left(\frac{1}{\sum_{i_k=1}^{|\mathbf{S}_k|}\delta_{i_k}} g\left(f^{-1}\left(\frac{1}{|\mathbf{S}_k|!}\sum_{p\in \mathbf{P}_{|\mathbf{S}_k|}} f\left(g^{-1}\left(\sum_{i_k=1}^{|\mathbf{S}_k|}\left(\delta_{i_k} g\left(f^{-1}\left((n\xi_{p(i_k)})f(\nu_{p(i_k)})\right)\right)\right)\right)\right)\right)\right)\right)\right\rangle$$

According to the sum operation in Equation (1), we have

$$\bigoplus_{k=1}^{N}\left(\frac{1}{|\boldsymbol{S}_k|!}\bigoplus_{p\in \boldsymbol{P}_{|S_k|}}\bigotimes_{i_k=1}^{|\boldsymbol{S}_k|}\left((n\xi_{p(i_k)})Q_{p(i_k)}\right)^{\delta_{i_k}}\right)^{\frac{1}{\sum_{i_k=1}^{|S_k|}\delta_{i_k}}}=\left\langle g^{-1}\left(\sum_{k=1}^{N}g\left(f^{-1}\left(\frac{1}{\sum_{i_k=1}^{|S_k|}\delta_{i_k}}f\left(g^{-1}\left(\frac{1}{|\boldsymbol{S}_k|!}\sum_{p\in \boldsymbol{P}_{|S_k|}}g\left(f^{-1}\left(\sum_{i_k=1}^{|S_k|}\left(\delta_{i_k}f\left(g^{-1}\left((n\xi_{p(i_k)})g(\mu_{p(i_k)})\right)\right)\right)\right)\right)\right)\right)\right)\right)\right),\right.$$

$$\left. f^{-1}\left(\sum_{k=1}^{N}f\left(g^{-1}\left(\frac{1}{\sum_{i_k=1}^{|S_k|}\delta_{i_k}}g\left(f^{-1}\left(\frac{1}{|\boldsymbol{S}_k|!}\sum_{p\in \boldsymbol{P}_{|S_k|}}f\left(g^{-1}\left(\sum_{i_k=1}^{|S_k|}\left(\delta_{i_k}g\left(f^{-1}\left((n\xi_{p(i_k)})f(\nu_{p(i_k)})\right)\right)\right)\right)\right)\right)\right)\right)\right)\right)\right\rangle$$

Finally, the following equation is obtained according to the multiplication operation in Equation (3)

$$\frac{1}{N}\left(\bigoplus_{k=1}^{N}\left(\frac{1}{|\boldsymbol{S}_k|!}\bigoplus_{p\in \boldsymbol{P}_{|S_k|}}\bigotimes_{i_k=1}^{|\boldsymbol{S}_k|}\left((n\xi_{p(i_k)})Q_{p(i_k)}\right)^{\delta_{i_k}}\right)^{\frac{1}{\sum_{i_k=1}^{|S_k|}\delta_{i_k}}}\right)=$$

$$\left\langle g^{-1}\left(\frac{1}{N}\sum_{k=1}^{N}g\left(f^{-1}\left(\frac{1}{\sum_{i_k=1}^{|S_k|}\delta_{i_k}}f\left(g^{-1}\left(\frac{1}{|\boldsymbol{S}_k|!}\sum_{p\in \boldsymbol{P}_{|S_k|}}g\left(f^{-1}\left(\sum_{i_k=1}^{|S_k|}\left(\delta_{i_k}f\left(g^{-1}\left((n\xi_{p(i_k)})g(\mu_{p(i_k)})\right)\right)\right)\right)\right)\right)\right)\right)\right)\right),\right.$$

$$\left. f^{-1}\left(\frac{1}{N}\sum_{k=1}^{N}f\left(g^{-1}\left(\frac{1}{\sum_{i_k=1}^{|S_k|}\delta_{i_k}}g\left(f^{-1}\left(\frac{1}{|\boldsymbol{S}_k|!}\sum_{p\in \boldsymbol{P}_{|S_k|}}f\left(g^{-1}\left(\sum_{i_k=1}^{|S_k|}\left(\delta_{i_k}g\left(f^{-1}\left((n\xi_{p(i_k)})f(\nu_{p(i_k)})\right)\right)\right)\right)\right)\right)\right)\right)\right)\right)\right\rangle=\langle\mu,\ \nu\rangle$$

Therefore, we have

$$\frac{1}{N}\left(\bigoplus_{k=1}^{N}\left(\frac{1}{|\boldsymbol{S}_k|!}\bigoplus_{p\in \boldsymbol{P}_{|S_k|}}\bigotimes_{i_k=1}^{|\boldsymbol{S}_k|}\left(\frac{n\left(1+T(Q_{p(i_k)})\right)}{\sum_{j=1}^{n}\left(1+T(Q_j)\right)}Q_{p(i_k)}\right)^{\delta_{i_k}}\right)^{\frac{1}{\sum_{i_k=1}^{|S_k|}\delta_{i_k}}}\right)=\langle\mu,\ \nu\rangle$$

(2) To prove $qROFAPPMM^{\Delta}(Q_1, Q_2, \ldots, Q_n)$ is still a qROFN, we need to prove $0 \le \mu \le 1$, $0 \le \nu \le 1$, and $0 \le \mu^q + \nu^q \le 1$. We firstly prove $0 \le \mu \le 1$ and $0 \le \nu \le 1$. The proof process is as follow:

1) According to the definition of a qROFN (Definition 1), we have $0 \le \mu_{p(i_k)} \le 1$. Because $g(t)$ is monotonically increasing, we further have

$$(n\xi_{p(i_k)})g(0)\le(n\xi_{p(i_k)})g(\mu_{p(i_k)})\le(n\xi_{p(i_k)})g(1)$$

Since $g^{-1}(t)$ is monotonically increasing, we can obtain

$$g^{-1}\left((n\xi_{p(i_k)})g(0)\right)\le g^{-1}\left((n\xi_{p(i_k)})g(\mu_{p(i_k)})\right)\le g^{-1}\left((n\xi_{p(i_k)})g(1)\right)$$

Because $f(t)$ is monotonically decreasing, we further have

$$\sum_{i_k=1}^{|S_k|}\left(\delta_{i_k}f\left(g^{-1}\left((n\xi_{p(i_k)})g(0)\right)\right)\right)\ge\sum_{i_k=1}^{|S_k|}\left(\delta_{i_k}f\left(g^{-1}\left((n\xi_{p(i_k)})g(\mu_{p(i_k)})\right)\right)\right)\ge\sum_{i_k=1}^{|S_k|}\left(\delta_{i_k}f\left(g^{-1}\left((n\xi_{p(i_k)})g(1)\right)\right)\right)$$

Since $f^{-1}(t)$ is monotonically decreasing, we can obtain

$$f^{-1}\left(\sum_{i_k=1}^{|S_k|}\left(\delta_{i_k}f\left(g^{-1}\left((n\xi_{p(i_k)})g(0)\right)\right)\right)\right)\le f^{-1}\left(\sum_{i_k=1}^{|S_k|}\left(\delta_{i_k}f\left(g^{-1}\left((n\xi_{p(i_k)})g(\mu_{p(i_k)})\right)\right)\right)\right)\le f^{-1}\left(\sum_{i_k=1}^{|S_k|}\left(\delta_{i_k}f\left(g^{-1}\left((n\xi_{p(i_k)})g(1)\right)\right)\right)\right)$$

Because $g(t)$ is monotonically increasing, we further have

$$g\left(f^{-1}\left(\sum_{i_k=1}^{|\boldsymbol{S}_k|}\left(\delta_{i_k} f\left(g^{-1}\left((n\xi_{p(i_k)})g(0)\right)\right)\right)\right)\right) \le \frac{1}{|\boldsymbol{S}_k|!}\sum_{p\in \boldsymbol{P}_{|\boldsymbol{S}_k|}} g\left(f^{-1}\left(\sum_{i_k=1}^{|\boldsymbol{S}_k|}\left(\delta_{i_k} f\left(g^{-1}\left((n\xi_{p(i_k)})g(\mu_{p(i_k)})\right)\right)\right)\right)\right) \le$$
$$g\left(f^{-1}\left(\sum_{i_k=1}^{|\boldsymbol{S}_k|}\left(\delta_{i_k} f\left(g^{-1}\left((n\xi_{p(i_k)})g(1)\right)\right)\right)\right)\right)$$

Since $g^{-1}(t)$ is monotonically increasing, we can obtain

$$f^{-1}\left(\sum_{i_k=1}^{|\boldsymbol{S}_k|}\left(\delta_{i_k} f\left(g^{-1}\left((n\xi_{p(i_k)})g(0)\right)\right)\right)\right) \le g^{-1}\left(\frac{1}{|\boldsymbol{S}_k|!}\sum_{p\in \boldsymbol{P}_{|\boldsymbol{S}_k|}} g\left(f^{-1}\left(\sum_{i_k=1}^{|\boldsymbol{S}_k|}\left(\delta_{i_k} f\left(g^{-1}\left((n\xi_{p(i_k)})g(\mu_{p(i_k)})\right)\right)\right)\right)\right)\right) \le f^{-1}\left(\sum_{i_k=1}^{|\boldsymbol{S}_k|}\left(\delta_{i_k} f\left(g^{-1}\left((n\xi_{p(i_k)})g(1)\right)\right)\right)\right)$$

Because $f(t)$ is monotonically decreasing, we further have

$$\frac{1}{\sum_{i_k=1}^{|\boldsymbol{S}_k|}\delta_{i_k}}\sum_{i_k=1}^{|\boldsymbol{S}_k|}\left(\delta_{i_k} f\left(g^{-1}\left((n\xi_{p(i_k)})g(0)\right)\right)\right) \ge \frac{1}{\sum_{i_k=1}^{|\boldsymbol{S}_k|}\delta_{i_k}} f\left(g^{-1}\left(\frac{1}{|\boldsymbol{S}_k|!}\sum_{p\in \boldsymbol{P}_{|\boldsymbol{S}_k|}} g\left(f^{-1}\left(\sum_{i_k=1}^{|\boldsymbol{S}_k|}\left(\delta_{i_k} f\left(g^{-1}\left((n\xi_{p(i_k)})g(\mu_{p(i_k)})\right)\right)\right)\right)\right)\right)\right) \ge$$
$$\frac{1}{\sum_{i_k=1}^{|\boldsymbol{S}_k|}\delta_{i_k}}\sum_{i_k=1}^{|\boldsymbol{S}_k|}\left(\delta_{i_k} f\left(g^{-1}\left((n\xi_{p(i_k)})g(1)\right)\right)\right)$$

Since $f^{-1}(t)$ is monotonically decreasing, we can obtain

$$f^{-1}\left(\frac{1}{\sum_{i_k=1}^{|\boldsymbol{S}_k|}\delta_{i_k}}\sum_{i_k=1}^{|\boldsymbol{S}_k|}\left(\delta_{i_k} f\left(g^{-1}\left((n\xi_{p(i_k)})g(0)\right)\right)\right)\right) \le f^{-1}\left(\frac{1}{\sum_{i_k=1}^{|\boldsymbol{S}_k|}\delta_{i_k}} f\left(g^{-1}\left(\frac{1}{|\boldsymbol{S}_k|!}\sum_{p\in \boldsymbol{P}_{|\boldsymbol{S}_k|}} g\left(f^{-1}\left(\sum_{i_k=1}^{|\boldsymbol{S}_k|}\left(\delta_{i_k} f\left(g^{-1}\left((n\xi_{p(i_k)})g(\mu_{p(i_k)})\right)\right)\right)\right)\right)\right)\right)\right) \le$$
$$f^{-1}\left(\frac{1}{\sum_{i_k=1}^{|\boldsymbol{S}_k|}\delta_{i_k}}\sum_{i_k=1}^{|\boldsymbol{S}_k|}\left(\delta_{i_k} f\left(g^{-1}\left((n\xi_{p(i_k)})g(1)\right)\right)\right)\right)$$

Because $g(t)$ is monotonically increasing, we further have

$$\frac{1}{N}\sum_{k=1}^{N} g\left(f^{-1}\left(\frac{1}{\sum_{i_k=1}^{|\boldsymbol{S}_k|}\delta_{i_k}}\sum_{i_k=1}^{|\boldsymbol{S}_k|}\left(\delta_{i_k} f\left(g^{-1}\left((n\xi_{p(i_k)})g(0)\right)\right)\right)\right)\right)$$
$$\le \frac{1}{N}\sum_{k=1}^{N} g\left(f^{-1}\left(\frac{1}{\sum_{i_k=1}^{|\boldsymbol{S}_k|}\delta_{i_k}} f\left(g^{-1}\left(\frac{1}{|\boldsymbol{S}_k|!}\sum_{p\in \boldsymbol{P}_{|\boldsymbol{S}_k|}} g\left(f^{-1}\left(\sum_{i_k=1}^{|\boldsymbol{S}_k|}\left(\delta_{i_k} f\left(g^{-1}\left((n\xi_{p(i_k)})g(\mu_{p(i_k)})\right)\right)\right)\right)\right)\right)\right)\right)\right) \le$$
$$\frac{1}{N}\sum_{k=1}^{N} g\left(f^{-1}\left(\frac{1}{\sum_{i_k=1}^{|\boldsymbol{S}_k|}\delta_{i_k}}\sum_{i_k=1}^{|\boldsymbol{S}_k|}\left(\delta_{i_k} f\left(g^{-1}\left((n\xi_{p(i_k)})g(1)\right)\right)\right)\right)\right)$$

Since

$$\sum_{k=1}^{N}\sum_{p\in \boldsymbol{P}_{|\boldsymbol{S}_k|}}\sum_{i_k=1}^{|\boldsymbol{S}_k|}(n\xi_{p(i_k)}) = n\left(\sum_{i_1=1}^{|\boldsymbol{S}_1|}\xi_{p(i_1)} + \sum_{i_2=1}^{|\boldsymbol{S}_2|}\xi_{p(i_2)} + \ldots + \sum_{i_N=1}^{|\boldsymbol{S}_N|}\xi_{p(i_N)}\right) = n\frac{1}{n} = 1$$

we can obtain

$$g(0)\le\frac{1}{N}\sum_{k=1}^{N}g\left(f^{-1}\left(\frac{1}{\sum_{i_k=1}^{|S_k|}\delta_{i_k}}f\left(g^{-1}\left(\frac{1}{|\boldsymbol{S}_k|!}\sum_{p\in\boldsymbol{P}_{|S_k|}}g\left(f^{-1}\left(\sum_{i_k=1}^{|S_k|}\left(\delta_{i_k}f\left(g^{-1}\left((n\xi_{p(i_k)})g(\mu_{p(i_k)})\right)\right)\right)\right)\right)\right)\right)\right)\right)\le g(1)$$

Finally, because $g^{-1}(t)$ is monotonically increasing and $f^{-1}(t)$ is monotonically decreasing, we have

$$0\le g^{-1}\left(\frac{1}{N}\sum_{k=1}^{N}g\left(f^{-1}\left(\frac{1}{\sum_{i_k=1}^{|S_k|}\delta_{i_k}}f\left(g^{-1}\left(\frac{1}{|\boldsymbol{S}_k|!}\sum_{p\in\boldsymbol{P}_{|S_k|}}g\left(f^{-1}\left(\sum_{i_k=1}^{|S_k|}\left(\delta_{i_k}f\left(g^{-1}\left((n\xi_{p(i_k)})g(\mu_{p(i_k)})\right)\right)\right)\right)\right)\right)\right)\right)\right)\right)\le 1$$

That is $0\le\mu\le 1$. Similarly, we can prove $0\le\nu\le 1$.

2) We then prove $0\le\mu^q+\nu^q\le 1$. The proof process is as follow:

Since $0\le\mu\le 1$ and $0\le\nu\le 1$, we have $0\le\mu^q\le 1$ and $0\le\nu^q\le 1$, and thus $0\le\mu^q+\nu^q\le 2$.

According to the definition of a qROFN (Definition 1), we have ${\mu_{p(i_k)}}^q+{\nu_{p(i_k)}}^q\le 1$ and thus ${\mu_{p(i_k)}}^q\le 1-{\nu_{p(i_k)}}^q$. Since $g(t)$ is monotonically increasing, we further have

$$(n\xi_{p(i_k)})g(\mu^q_{p(i_k)})\le(n\xi_{p(i_k)})g(1-\nu^q_{p(i_k)})$$

Because $g(1-t)=f(t)$, we have

$$(n\xi_{p(i_k)})g(\mu^q_{p(i_k)})\le(n\xi_{p(i_k)})f(\nu^q_{p(i_k)})$$

Since $g^{-1}(t)$ is monotonically increasing, we can obtain

$$g^{-1}\left((n\xi_{p(i_k)})g(\mu^q_{p(i_k)})\right)\le g^{-1}\left((n\xi_{p(i_k)})f(\nu^q_{p(i_k)})\right)$$

Because $g^{-1}(t)=1-f^{-1}(t)$, we have

$$g^{-1}\left((n\xi_{p(i_k)})g(\mu^q_{p(i_k)})\right)\le 1-f^{-1}\left((n\xi_{p(i_k)})f(\nu^q_{p(i_k)})\right)$$

Since $f(t)$ is monotonically decreasing, we further have

$$\sum_{i_k=1}^{|S_k|}\left(\delta_{i_k}f\left(g^{-1}\left((n\xi_{p(i_k)})g(\mu^q_{p(i_k)})\right)\right)\right)\ge\sum_{i_k=1}^{|S_k|}\left(\delta_{i_k}f\left(1-f^{-1}\left((n\xi_{p(i_k)})f(\nu^q_{p(i_k)})\right)\right)\right)$$

Because $f(1-t)=g(t)$, we can obtain

$$\sum_{i_k=1}^{|S_k|}\left(\delta_{i_k}f\left(g^{-1}\left((n\xi_{p(i_k)})g(\mu^q_{p(i_k)})\right)\right)\right)\ge\sum_{i_k=1}^{|S_k|}\left(\delta_{i_k}g\left(f^{-1}\left((n\xi_{p(i_k)})f(\nu^q_{p(i_k)})\right)\right)\right)$$

Since $f^{-1}(t)$ is monotonically decreasing, we have

$$f^{-1}\left(\sum_{i_k=1}^{|S_k|}\left(\delta_{i_k}f\left(g^{-1}\left((n\xi_{p(i_k)})g(\mu^q_{p(i_k)})\right)\right)\right)\right)\le f^{-1}\left(\sum_{i_k=1}^{|S_k|}\left(\delta_{i_k}g\left(f^{-1}\left((n\xi_{p(i_k)})f(\nu^q_{p(i_k)})\right)\right)\right)\right)$$

Because $f^{-1}(t)=1-g^{-1}(t)$, we further have

$$f^{-1}\left(\sum_{i_k=1}^{|S_k|}\left(\delta_{i_k}f\left(g^{-1}\left((n\xi_{p(i_k)})g(\mu^q_{p(i_k)})\right)\right)\right)\right)\le 1-g^{-1}\left(\sum_{i_k=1}^{|S_k|}\left(\delta_{i_k}g\left(f^{-1}\left((n\xi_{p(i_k)})f(\nu^q_{p(i_k)})\right)\right)\right)\right)$$

Since $g(t)$ is monotonically increasing, we can obtain

$$\frac{1}{|\boldsymbol{S}_k|!}\sum_{p\in\boldsymbol{P}_{|S_k|}}g\left(f^{-1}\left(\sum_{i_k=1}^{|S_k|}\left(\delta_{i_k}f\left(g^{-1}\left((n\xi_{p(i_k)})g(\mu^q_{p(i_k)})\right)\right)\right)\right)\right)\le\frac{1}{|\boldsymbol{S}_k|!}\sum_{p\in\boldsymbol{P}_{|S_k|}}g\left(1-g^{-1}\left(\sum_{i_k=1}^{|S_k|}\left(\delta_{i_k}g\left(f^{-1}\left((n\xi_{p(i_k)})f(\nu^q_{p(i_k)})\right)\right)\right)\right)\right)$$

Because $g(1-t)=f(t)$, we can obtain

$$\frac{1}{|\boldsymbol{S}_k|!}\sum_{p\in\boldsymbol{P}_{|S_k|}}g\left(f^{-1}\left(\sum_{i_k=1}^{|S_k|}\left(\delta_{i_k}f\left(g^{-1}\left((n\xi_{p(i_k)})g(\mu^q_{p(i_k)})\right)\right)\right)\right)\right)\le\frac{1}{|\boldsymbol{S}_k|!}\sum_{p\in\boldsymbol{P}_{|S_k|}}f\left(g^{-1}\left(\sum_{i_k=1}^{|S_k|}\left(\delta_{i_k}g\left(f^{-1}\left((n\xi_{p(i_k)})f(\nu^q_{p(i_k)})\right)\right)\right)\right)\right)$$

Since $g^{-1}(t)$ is monotonically increasing, we have

$$g^{-1}\left(\frac{1}{|\boldsymbol{S}_k|!}\sum_{p\in\boldsymbol{P}_{|S_k|}} g\left(f^{-1}\left(\sum_{i_k=1}^{|S_k|}\left(\delta_{i_k} f\left(g^{-1}\left((n\xi_{p(i_k)})g(\mu^q_{p(i_k)})\right)\right)\right)\right)\right)\right) \le g^{-1}\left(\frac{1}{|\boldsymbol{S}_k|!}\sum_{p\in\boldsymbol{P}_{|S_k|}} f\left(g^{-1}\left(\sum_{i_k=1}^{|S_k|}\left(\delta_{i_k} g\left(f^{-1}\left((n\xi_{p(i_k)})f(\nu^q_{p(i_k)})\right)\right)\right)\right)\right)\right)$$

Because $g^{-1}(t) = 1 - f^{-1}(t)$, we further have

$$g^{-1}\left(\frac{1}{|\boldsymbol{S}_k|!}\sum_{p\in\boldsymbol{P}_{|S_k|}} g\left(f^{-1}\left(\sum_{i_k=1}^{|S_k|}\left(\delta_{i_k} f\left(g^{-1}\left((n\xi_{p(i_k)})g(\mu^q_{p(i_k)})\right)\right)\right)\right)\right)\right) \le 1 - f^{-1}\left(\frac{1}{|\boldsymbol{S}_k|!}\sum_{p\in\boldsymbol{P}_{|S_k|}} f\left(g^{-1}\left(\sum_{i_k=1}^{|S_k|}\left(\delta_{i_k} g\left(f^{-1}\left((n\xi_{p(i_k)})f(\nu^q_{p(i_k)})\right)\right)\right)\right)\right)\right)$$

Since $f(t)$ is monotonically decreasing, we can obtain

$$\frac{1}{\sum_{i_k=1}^{|S_k|}\delta_{i_k}} f\left(g^{-1}\left(\frac{1}{|\boldsymbol{S}_k|!}\sum_{p\in\boldsymbol{P}_{|S_k|}} g\left(f^{-1}\left(\sum_{i_k=1}^{|S_k|}\left(\delta_{i_k} f\left(g^{-1}\left((n\xi_{p(i_k)})g(\mu^q_{p(i_k)})\right)\right)\right)\right)\right)\right)\right) \ge$$

$$\frac{1}{\sum_{i_k=1}^{|S_k|}\delta_{i_k}} f\left(1 - f^{-1}\left(\frac{1}{|\boldsymbol{S}_k|!}\sum_{p\in\boldsymbol{P}_{|S_k|}} f\left(g^{-1}\left(\sum_{i_k=1}^{|S_k|}\left(\delta_{i_k} g\left(f^{-1}\left((n\xi_{p(i_k)})f(\nu^q_{p(i_k)})\right)\right)\right)\right)\right)\right)\right)$$

Because $f(1-t) = g(t)$, we can obtain

$$\frac{1}{\sum_{i_k=1}^{|S_k|}\delta_{i_k}} f\left(g^{-1}\left(\frac{1}{|\boldsymbol{S}_k|!}\sum_{p\in\boldsymbol{P}_{|S_k|}} g\left(f^{-1}\left(\sum_{i_k=1}^{|S_k|}\left(\delta_{i_k} f\left(g^{-1}\left((n\xi_{p(i_k)})g(\mu^q_{p(i_k)})\right)\right)\right)\right)\right)\right)\right) \ge$$

$$\frac{1}{\sum_{i_k=1}^{|S_k|}\delta_{i_k}} g\left(f^{-1}\left(\frac{1}{|\boldsymbol{S}_k|!}\sum_{p\in\boldsymbol{P}_{|S_k|}} f\left(g^{-1}\left(\sum_{i_k=1}^{|S_k|}\left(\delta_{i_k} g\left(f^{-1}\left((n\xi_{p(i_k)})f(\nu^q_{p(i_k)})\right)\right)\right)\right)\right)\right)\right)$$

Since $f^{-1}(t)$ is monotonically decreasing, we have

$$f^{-1}\left(\frac{1}{\sum_{i_k=1}^{|S_k|}\delta_{i_k}} f\left(g^{-1}\left(\frac{1}{|\boldsymbol{S}_k|!}\sum_{p\in\boldsymbol{P}_{|S_k|}} g\left(f^{-1}\left(\sum_{i_k=1}^{|S_k|}\left(\delta_{i_k} f\left(g^{-1}\left((n\xi_{p(i_k)})g(\mu^q_{p(i_k)})\right)\right)\right)\right)\right)\right)\right)\right) \le$$

$$f^{-1}\left(\frac{1}{\sum_{i_k=1}^{|S_k|}\delta_{i_k}} g\left(f^{-1}\left(\frac{1}{|\boldsymbol{S}_k|!}\sum_{p\in\boldsymbol{P}_{|S_k|}} f\left(g^{-1}\left(\sum_{i_k=1}^{|S_k|}\left(\delta_{i_k} g\left(f^{-1}\left((n\xi_{p(i_k)})f(\nu^q_{p(i_k)})\right)\right)\right)\right)\right)\right)\right)\right)$$

Because $f^{-1}(t) = 1 - g^{-1}(t)$, we can obtain

$$f^{-1}\left(\frac{1}{\sum_{i_k=1}^{|S_k|}\delta_{i_k}} f\left(g^{-1}\left(\frac{1}{|\boldsymbol{S}_k|!}\sum_{p\in\boldsymbol{P}_{|S_k|}} g\left(f^{-1}\left(\sum_{i_k=1}^{|S_k|}\left(\delta_{i_k} f\left(g^{-1}\left((n\xi_{p(i_k)})g(\mu^q_{p(i_k)})\right)\right)\right)\right)\right)\right)\right)\right) \le$$

$$1 - g^{-1}\left(\frac{1}{\sum_{i_k=1}^{|S_k|}\delta_{i_k}} g\left(f^{-1}\left(\frac{1}{|\boldsymbol{S}_k|!}\sum_{p\in\boldsymbol{P}_{|S_k|}} f\left(g^{-1}\left(\sum_{i_k=1}^{|S_k|}\left(\delta_{i_k} g\left(f^{-1}\left((n\xi_{p(i_k)})f(\nu^q_{p(i_k)})\right)\right)\right)\right)\right)\right)\right)\right)$$

Since $g(t)$ is monotonically increasing, we have

$$\frac{1}{N}\sum_{k=1}^{N} g\left(f^{-1}\left(\frac{1}{\sum_{i_k=1}^{|S_k|}\delta_{i_k}} f\left(g^{-1}\left(\frac{1}{|\boldsymbol{S}_k|!}\sum_{p\in\boldsymbol{P}_{|S_k|}} g\left(f^{-1}\left(\sum_{i_k=1}^{|S_k|}\left(\delta_{i_k} f\left(g^{-1}\left((n\xi_{p(i_k)})g(\mu^q_{p(i_k)})\right)\right)\right)\right)\right)\right)\right)\right)\right) \le$$

$$\frac{1}{N}\sum_{k=1}^{N} g\left(1-g^{-1}\left(\frac{1}{\sum_{i_k=1}^{|S_k|}\delta_{i_k}} g\left(f^{-1}\left(\frac{1}{|\boldsymbol{S}_k|!}\sum_{p\in \boldsymbol{P}_{|S_k|}} f\left(g^{-1}\left(\sum_{i_k=1}^{|S_k|}\left(\delta_{i_k} g\left(f^{-1}\left((n\xi_{p(i_k)}) f(\nu_{p(i_k)}^q)\right)\right)\right)\right)\right)\right)\right)\right)\right)$$

Because $g(1-t) = f(t)$, we can obtain

$$\frac{1}{N}\sum_{k=1}^{N} g\left(f^{-1}\left(\frac{1}{\sum_{i_k=1}^{|S_k|}\delta_{i_k}} f\left(g^{-1}\left(\frac{1}{|\boldsymbol{S}_k|!}\sum_{p\in \boldsymbol{P}_{|S_k|}} g\left(f^{-1}\left(\sum_{i_k=1}^{|S_k|}\left(\delta_{i_k} f\left(g^{-1}\left((n\xi_{p(i_k)}) g(\mu_{p(i_k)}^q)\right)\right)\right)\right)\right)\right)\right)\right)\right) \le$$

$$\frac{1}{N}\sum_{k=1}^{N} f\left(g^{-1}\left(\frac{1}{\sum_{i_k=1}^{|S_k|}\delta_{i_k}} g\left(f^{-1}\left(\frac{1}{|\boldsymbol{S}_k|!}\sum_{p\in \boldsymbol{P}_{|S_k|}} f\left(g^{-1}\left(\sum_{i_k=1}^{|S_k|}\left(\delta_{i_k} g\left(f^{-1}\left((n\xi_{p(i_k)}) f(\nu_{p(i_k)}^q)\right)\right)\right)\right)\right)\right)\right)\right)\right)$$

Since $g^{-1}(t)$ is monotonically increasing, we have

$$g^{-1}\left(\frac{1}{N}\sum_{k=1}^{N} g\left(f^{-1}\left(\frac{1}{\sum_{i_k=1}^{|S_k|}\delta_{i_k}} f\left(g^{-1}\left(\frac{1}{|\boldsymbol{S}_k|!}\sum_{p\in \boldsymbol{P}_{|S_k|}} g\left(f^{-1}\left(\sum_{i_k=1}^{|S_k|}\left(\delta_{i_k} f\left(g^{-1}\left((n\xi_{p(i_k)}) g(\mu_{p(i_k)}^q)\right)\right)\right)\right)\right)\right)\right)\right)\right)\right) \le$$

$$g^{-1}\left(\frac{1}{N}\sum_{k=1}^{N} f\left(g^{-1}\left(\frac{1}{\sum_{i_k=1}^{|S_k|}\delta_{i_k}} g\left(f^{-1}\left(\frac{1}{|\boldsymbol{S}_k|!}\sum_{p\in \boldsymbol{P}_{|S_k|}} f\left(g^{-1}\left(\sum_{i_k=1}^{|S_k|}\left(\delta_{i_k} g\left(f^{-1}\left((n\xi_{p(i_k)}) f(\nu_{p(i_k)}^q)\right)\right)\right)\right)\right)\right)\right)\right)\right)\right)$$

Finally, because $g^{-1}(t) = 1 - f^{-1}(t)$, we can obtain

$$g^{-1}\left(\frac{1}{N}\sum_{k=1}^{N} g\left(f^{-1}\left(\frac{1}{\sum_{i_k=1}^{|S_k|}\delta_{i_k}} f\left(g^{-1}\left(\frac{1}{|\boldsymbol{S}_k|!}\sum_{p\in \boldsymbol{P}_{|S_k|}} g\left(f^{-1}\left(\sum_{i_k=1}^{|S_k|}\left(\delta_{i_k} f\left(g^{-1}\left((n\xi_{p(i_k)}) g(\mu_{p(i_k)}^q)\right)\right)\right)\right)\right)\right)\right)\right)\right)\right) \le$$

$$1-f^{-1}\left(\frac{1}{N}\sum_{k=1}^{N} f\left(g^{-1}\left(\frac{1}{\sum_{i_k=1}^{|S_k|}\delta_{i_k}} g\left(f^{-1}\left(\frac{1}{|\boldsymbol{S}_k|!}\sum_{p\in \boldsymbol{P}_{|S_k|}} f\left(g^{-1}\left(\sum_{i_k=1}^{|S_k|}\left(\delta_{i_k} g\left(f^{-1}\left((n\xi_{p(i_k)}) f(\nu_{p(i_k)}^q)\right)\right)\right)\right)\right)\right)\right)\right)\right)\right)$$

When $q = 1$, according to the above inequality, we have

$$\mu \le 1 - \nu$$

Therefore, we can obtain

$$\mu + \nu \le 1$$

Now we need to prove the inequality also holds when $q = 2, 3, \ldots$. Let $m = 2, 3, \ldots$. The purpose is transformed into proof of $\mu^m + \nu^m \le 1$.

According to $\mu + \nu \le 1$ and the binomial theorem, we can obtain

$$(\mu+\nu)^m = \sum_{k=0}^{m}\left(C_m^k \mu^{m-k}\nu^k\right) = \mu^m + \nu^m + \sum_{k=1}^{m-1}\left(C_m^k \mu^{m-k}\nu^k\right) \le 1$$

Because $\mu \ge 0$ and $\nu \ge 0$, we have

$$\sum_{k=1}^{m-1}\left(C_m^k \mu^{m-k}\nu^k\right) \ge 0$$

Therefore, we can obtain $\mu^m + \nu^m \le 1$. Now it can be concluded that $\mu^q + \nu^q \le 1$ for $q = 1, 2, 3, \ldots$.

Since we have proved $0 \le \mu^q + \nu^q \le 2$ and $\mu^q + \nu^q \le 1$, we can obtain $0 \le \mu^q + \nu^q \le 1$. □

Appendix B. Proof of Theorem 2

Proof.

According to Theorem 1, we have

$$qROFAPPMM^{\Lambda}(Q_1,Q_2,...,Q_n)=$$

$$\left\langle g^{-1}\left(\frac{1}{N}\sum_{k=1}^{N} g\left(f^{-1}\left(\frac{1}{\sum_{i_k=1}^{|\boldsymbol{S}_k|}\delta_{i_k}} f\left(g^{-1}\left(\frac{1}{|\boldsymbol{S}_k|!}\sum_{p\in \boldsymbol{P}_{|\boldsymbol{S}_k|}} g\left(f^{-1}\left(\sum_{i_k=1}^{|\boldsymbol{S}_k|}\left(\delta_{i_k} f\left(g^{-1}\left(\frac{n\left(1+T(Q_{p(i_k)})\right)}{\sum_{j=1}^{n}\left(1+T(Q_j)\right)} g(\mu_{p(i_k)})\right)\right)\right)\right)\right)\right)\right)\right)\right)\right),\right.$$

$$\left. f^{-1}\left(\frac{1}{N}\sum_{k=1}^{N} f\left(g^{-1}\left(\frac{1}{\sum_{i_k=1}^{|\boldsymbol{S}_k|}\delta_{i_k}} g\left(f^{-1}\left(\frac{1}{|\boldsymbol{S}_k|!}\sum_{p\in \boldsymbol{P}_{|\boldsymbol{S}_k|}} f\left(g^{-1}\left(\sum_{i_k=1}^{|\boldsymbol{S}_k|}\left(\delta_{i_k} g\left(f^{-1}\left(\frac{n\left(1+T(Q_{p(i_k)})\right)}{\sum_{j=1}^{n}\left(1+T(Q_j)\right)} f(\nu_{p(i_k)})\right)\right)\right)\right)\right)\right)\right)\right)\right)\right)\right\rangle$$

Since $Q_i = Q = <\mu_Q, \nu_Q>$ for all $i = 1, 2, \ldots, n$, we have $D(Q_i, Q_j) = 0$ for all $j = 1, 2, \ldots, n$ and $j \neq i$. According to the expression of $T(Q_r)$ in Definition 12, we can obtain

$$\frac{n\sum_{k=1}^{N}\sum_{p\in \boldsymbol{P}_{|\boldsymbol{S}_k|}}\sum_{i_k=1}^{|\boldsymbol{S}_k|}\left(1+T(Q_{p(i_k)})\right)}{\sum_{j=1}^{n}\left(1+T(Q_j)\right)}=\frac{n\left(1+(n-1)\right)}{n\left(1+(n-1)\right)}=1$$

Therefore, we have

$$qROFAPPMM^{\Lambda}(Q_1,Q_2,...,Q_n)=\left\langle g^{-1}\left(\frac{1}{N}\sum_{k=1}^{N} g\left(f^{-1}\left(\frac{1}{\sum_{i_k=1}^{|\boldsymbol{S}_k|}\delta_{i_k}} f\left(g^{-1}\left(\frac{1}{|\boldsymbol{S}_k|!}\sum_{p\in \boldsymbol{P}_{|\boldsymbol{S}_k|}} g\left(f^{-1}\left(\sum_{i_k=1}^{|\boldsymbol{S}_k|}\left(\delta_{i_k} f(\mu_{p(i_k)})\right)\right)\right)\right)\right)\right)\right)\right),\right.$$

$$\left. f^{-1}\left(\frac{1}{N}\sum_{k=1}^{N} f\left(g^{-1}\left(\frac{1}{\sum_{i_k=1}^{|\boldsymbol{S}_k|}\delta_{i_k}} g\left(f^{-1}\left(\frac{1}{|\boldsymbol{S}_k|!}\sum_{p\in \boldsymbol{P}_{|\boldsymbol{S}_k|}} f\left(g^{-1}\left(\sum_{i_k=1}^{|\boldsymbol{S}_k|}\left(\delta_{i_k} g(\nu_{p(i_k)})\right)\right)\right)\right)\right)\right)\right)\right)\right\rangle$$

Since $\mu_i = \mu_Q$ and $p(i_k)$ is a permutation of $(1, 2, \ldots, |\boldsymbol{S}_k|)$, we have

$$\sum_{i_k=1}^{|\boldsymbol{S}_k|}\left(\delta_{i_k} f(\mu_{p(i_k)})\right)=\sum_{i_k=1}^{|\boldsymbol{S}_k|}\left(\delta_{i_k} f(\mu_Q)\right)=\left(\sum_{i_k=1}^{|\boldsymbol{S}_k|}\delta_{i_k}\right) f(\mu_Q)$$

and

$$f^{-1}\left(\sum_{i_k=1}^{|\boldsymbol{S}_k|}\left(\delta_{i_k} f(\mu_{p(i_k)})\right)\right)=f^{-1}\left(\left(\sum_{i_k=1}^{|\boldsymbol{S}_k|}\delta_{i_k}\right) f(\mu_Q)\right)$$

Then we can obtain

$$\frac{1}{|\boldsymbol{S}_k|!}\sum_{p\in \boldsymbol{P}_{|\boldsymbol{S}_k|}} g\left(f^{-1}\left(\sum_{i_k=1}^{|\boldsymbol{S}_k|}\left(\delta_{i_k} f(\mu_{p(i_k)})\right)\right)\right)=\frac{1}{|\boldsymbol{S}_k|!}\sum_{p\in \boldsymbol{P}_{|\boldsymbol{S}_k|}} g\left(f^{-1}\left(\left(\sum_{i_k=1}^{|\boldsymbol{S}_k|}\delta_{i_k}\right) f(\mu_Q)\right)\right)=g\left(f^{-1}\left(\left(\sum_{i_k=1}^{|\boldsymbol{S}_k|}\delta_{i_k}\right) f(\mu_Q)\right)\right)$$

and

$$g^{-1}\left(\frac{1}{|\boldsymbol{S}_k|!}\sum_{p\in \boldsymbol{P}_{|\boldsymbol{S}_k|}} g\left(f^{-1}\left(\sum_{i_k=1}^{|\boldsymbol{S}_k|}\left(\delta_{i_k} f(\mu_{p(i_k)})\right)\right)\right)\right)=g^{-1}\left(g\left(f^{-1}\left(\left(\sum_{i_k=1}^{|\boldsymbol{S}_k|}\delta_{i_k}\right) f(\mu_Q)\right)\right)\right)=f^{-1}\left(\left(\sum_{i_k=1}^{|\boldsymbol{S}_k|}\delta_{i_k}\right) f(\mu_Q)\right)$$

Further, we have

$$\frac{1}{\sum_{i_k=1}^{|S_k|}\delta_{i_k}} f\left(g^{-1}\left(\frac{1}{|\boldsymbol{S}_k|!}\sum_{p\in \boldsymbol{P}_{|S_k|}} g\left(f^{-1}\left(\sum_{i_k=1}^{|S_k|}\left(\delta_{i_k} f(\mu_{p(i_k)})\right)\right)\right)\right)\right) = \frac{1}{\sum_{i_k=1}^{|S_k|}\delta_{i_k}} f\left(f^{-1}\left(\left(\sum_{i_k=1}^{|S_k|}\delta_{i_k}\right) f(\mu_Q)\right)\right) = f(\mu_Q)$$

and

$$f^{-1}\left(\frac{1}{\sum_{i_k=1}^{|S_k|}\delta_{i_k}} f\left(g^{-1}\left(\frac{1}{|\boldsymbol{S}_k|!}\sum_{p\in \boldsymbol{P}_{|S_k|}} g\left(f^{-1}\left(\sum_{i_k=1}^{|S_k|}\left(\delta_{i_k} f(\mu_{p(i_k)})\right)\right)\right)\right)\right)\right) = f^{-1}\left(f(\mu_Q)\right) = \mu_Q$$

Finally, we can obtain

$$\frac{1}{N}\sum_{k=1}^{N} g\left(f^{-1}\left(\frac{1}{\sum_{i_k=1}^{|S_k|}\delta_{i_k}} f\left(g^{-1}\left(\frac{1}{|\boldsymbol{S}_k|!}\sum_{p\in \boldsymbol{P}_{|S_k|}} g\left(f^{-1}\left(\sum_{i_k=1}^{|S_k|}\left(\delta_{i_k} f(\mu_{p(i_k)})\right)\right)\right)\right)\right)\right)\right) = \frac{1}{N}\sum_{k=1}^{N} g(\mu_Q) = g(\mu_Q)$$

and

$$g^{-1}\left(\frac{1}{N}\sum_{k=1}^{N} g\left(f^{-1}\left(\frac{1}{\sum_{i_k=1}^{|S_k|}\delta_{i_k}} f\left(g^{-1}\left(\frac{1}{|\boldsymbol{S}_k|!}\sum_{p\in \boldsymbol{P}_{|S_k|}} g\left(f^{-1}\left(\sum_{i_k=1}^{|S_k|}\left(\delta_{i_k} f(\mu_{p(i_k)})\right)\right)\right)\right)\right)\right)\right)\right) = g^{-1}\left(g(\mu_Q)\right) = \mu_Q$$

Similarly, we can prove

$$f^{-1}\left(\frac{1}{N}\sum_{k=1}^{N} f\left(g^{-1}\left(\frac{1}{\sum_{i_k=1}^{|S_k|}\delta_{i_k}} g\left(f^{-1}\left(\frac{1}{|\boldsymbol{S}_k|!}\sum_{p\in \boldsymbol{P}_{|S_k|}} f\left(g^{-1}\left(\sum_{i_k=1}^{|S_k|}\left(\delta_{i_k} g(\nu_{p(i_k)})\right)\right)\right)\right)\right)\right)\right)\right) = f^{-1}\left(f(\nu_Q)\right) = \nu_Q$$

Therefore, we can obtain $qROFAPPMM^{\boldsymbol{\Delta}}(Q_1, Q_2, \ldots, Q_n) = <\mu_Q, \nu_Q>$. □

Appendix C. Proof of Theorem 3

Proof.

According to Theorem 1, we have $qROFAPPMM^{\boldsymbol{\Delta}}(Q_1, Q_2, \ldots, Q_n) = <\mu, \nu>$, where

$$\mu = g^{-1}\left(\frac{1}{N}\sum_{k=1}^{N} g\left(f^{-1}\left(\frac{1}{\sum_{i_k=1}^{|S_k|}\delta_{i_k}} f\left(g^{-1}\left(\frac{1}{|\boldsymbol{S}_k|!}\sum_{p\in \boldsymbol{P}_{|S_k|}} g\left(f^{-1}\left(\sum_{i_k=1}^{|S_k|}\left(\delta_{i_k} f\left(g^{-1}\left((n\xi_{p(i_k)})g(\mu_{p(i_k)})\right)\right)\right)\right)\right)\right)\right)\right)\right)\right),$$

$$\nu = f^{-1}\left(\frac{1}{N}\sum_{k=1}^{N} f\left(g^{-1}\left(\frac{1}{\sum_{i_k=1}^{|S_k|}\delta_{i_k}} g\left(f^{-1}\left(\frac{1}{|\boldsymbol{S}_k|!}\sum_{p\in \boldsymbol{P}_{|S_k|}} f\left(g^{-1}\left(\sum_{i_k=1}^{|S_k|}\left(\delta_{i_k} g\left(f^{-1}\left((n\xi_{p(i_k)}) f(\nu_{p(i_k)})\right)\right)\right)\right)\right)\right)\right)\right)\right)\right), \quad \text{and} \quad \xi_{p(i_k)} = \frac{1+T(Q_{p(i_k)})}{\sum_{j=1}^{n}\left(1+T(Q_j)\right)}$$

According to Theorem 2, we have $Q_{\mathrm{LB}} = qROFAPPMM^{\boldsymbol{\Delta}}(Q_{\mathrm{LB}}, Q_{\mathrm{LB}}, \ldots, Q_{\mathrm{LB}})$, $Q_{\mathrm{UB}} = qROFAPPMM^{\boldsymbol{\Delta}}(Q_{\mathrm{UB}}, Q_{\mathrm{UB}}, \ldots, Q_{\mathrm{UB}})$, and $n\xi_{p(i_k)} = 1$ for $qROFAPPMM^{\boldsymbol{\Delta}}(Q_{\mathrm{LB}}, Q_{\mathrm{LB}}, \ldots, Q_{\mathrm{LB}})$ and $qROFAPPMM^{\boldsymbol{\Delta}}(Q_{\mathrm{UB}}, Q_{\mathrm{UB}}, \ldots, Q_{\mathrm{UB}})$. Since $\mu_{\mathrm{LB}} \le \mu_{p(i_k)} \le \mu_{\mathrm{UB}}$ and $g(x)$ is monotonically increasing, we can obtain

$$g(\mu_{\mathrm{LB}}) = (n\xi_{p(i_k)})g(\mu_{\mathrm{LB}}) \le (n\xi_{p(i_k)})g(\mu_{p(i_k)}) \le (n\xi_{p(i_k)})g(\mu_{\mathrm{UB}}) = g(\mu_{\mathrm{UB}})$$

Because $g^{-1}(x)$ is monotonically increasing, we have

$$\mu_{\mathrm{LB}} = g^{-1}\left(g(\mu_{\mathrm{LB}})\right) \le g^{-1}\left((n\xi_{p(i_k)})g(\mu_{p(i_k)})\right) \le g^{-1}\left(g(\mu_{\mathrm{UB}})\right) = \mu_{\mathrm{UB}}$$

Since $f(x)$ is monotonically decreasing, we can obtain

$$\left(\sum_{i_k=1}^{|\boldsymbol{S}_k|}\delta_{i_k}\right) f(\mu_{\mathrm{LB}}) = \sum_{i_k=1}^{|\boldsymbol{S}_k|}\left(\delta_{i_k} f(\mu_{\mathrm{LB}})\right) \geq \sum_{i_k=1}^{|\boldsymbol{S}_k|}\left(\delta_{i_k} f\left(g^{-1}\left((n\xi_{p(i_k)})g(\mu_{p(i_k)})\right)\right)\right) \geq \sum_{i_k=1}^{|\boldsymbol{S}_k|}\left(\delta_{i_k} f(\mu_{\mathrm{UB}})\right) = \left(\sum_{i_k=1}^{|\boldsymbol{S}_k|}\delta_{i_k}\right) f(\mu_{\mathrm{UB}})$$

Because $f^{-1}(x)$ is monotonically decreasing, we have

$$f^{-1}\left(\left(\sum_{i_k=1}^{|\boldsymbol{S}_k|}\delta_{i_k}\right) f(\mu_{\mathrm{LB}})\right) \leq f^{-1}\left(\sum_{i_k=1}^{|\boldsymbol{S}_k|}\left(\delta_{i_k} f\left(g^{-1}\left((n\xi_{p(i_k)})g(\mu_{p(i_k)})\right)\right)\right)\right) \leq f^{-1}\left(\left(\sum_{i_k=1}^{|\boldsymbol{S}_k|}\delta_{i_k}\right) f(\mu_{\mathrm{UB}})\right)$$

Since $g(x)$ is monotonically increasing, we can obtain

$$g\left(f^{-1}\left(\left(\sum_{i_k=1}^{|\boldsymbol{S}_k|}\delta_{i_k}\right) f(\mu_{\mathrm{LB}})\right)\right) = \frac{1}{|\boldsymbol{S}_k|!}\sum_{p\in \boldsymbol{P}_{|\boldsymbol{S}_k|}} g\left(f^{-1}\left(\left(\sum_{i_k=1}^{|\boldsymbol{S}_k|}\delta_{i_k}\right) f(\mu_{\mathrm{LB}})\right)\right) \leq \frac{1}{|\boldsymbol{S}_k|!}\sum_{p\in \boldsymbol{P}_{|\boldsymbol{S}_k|}} g\left(f^{-1}\left(\sum_{i_k=1}^{|\boldsymbol{S}_k|}\left(\delta_{i_k} f\left(g^{-1}\left((n\xi_{p(i_k)})g(\mu_{p(i_k)})\right)\right)\right)\right)\right) \leq$$
$$\frac{1}{|\boldsymbol{S}_k|!}\sum_{p\in \boldsymbol{P}_{|\boldsymbol{S}_k|}} g\left(f^{-1}\left(\left(\sum_{i_k=1}^{|\boldsymbol{S}_k|}\delta_{i_k}\right) f(\mu_{\mathrm{UB}})\right)\right) = g\left(f^{-1}\left(\left(\sum_{i_k=1}^{|\boldsymbol{S}_k|}\delta_{i_k}\right) f(\mu_{\mathrm{UB}})\right)\right)$$

Because $g^{-1}(x)$ is monotonically increasing, we have

$$f^{-1}\left(\left(\sum_{i_k=1}^{|\boldsymbol{S}_k|}\delta_{i_k}\right) f(\mu_{\mathrm{LB}})\right) = g^{-1}\left(g\left(f^{-1}\left(\left(\sum_{i_k=1}^{|\boldsymbol{S}_k|}\delta_{i_k}\right) f(\mu_{\mathrm{LB}})\right)\right)\right) \leq g^{-1}\left(\frac{1}{|\boldsymbol{S}_k|!}\sum_{p\in \boldsymbol{P}_{|\boldsymbol{S}_k|}} g\left(f^{-1}\left(\sum_{i_k=1}^{|\boldsymbol{S}_k|}\left(\delta_{i_k} f\left(g^{-1}\left((n\xi_{p(i_k)})g(\mu_{p(i_k)})\right)\right)\right)\right)\right)\right) \leq$$
$$g^{-1}\left(g\left(f^{-1}\left(\left(\sum_{i_k=1}^{|\boldsymbol{S}_k|}\delta_{i_k}\right) f(\mu_{\mathrm{UB}})\right)\right)\right) = f^{-1}\left(\left(\sum_{i_k=1}^{|\boldsymbol{S}_k|}\delta_{i_k}\right) f(\mu_{\mathrm{UB}})\right)$$

Since $f(x)$ is monotonically decreasing, we can obtain

$$f(\mu_{\mathrm{LB}}) = \frac{1}{\sum_{i_k=1}^{|\boldsymbol{S}_k|}\delta_{i_k}} f\left(f^{-1}\left(\left(\sum_{i_k=1}^{|\boldsymbol{S}_k|}\delta_{i_k}\right) f(\mu_{\mathrm{LB}})\right)\right) \geq \frac{1}{\sum_{i_k=1}^{|\boldsymbol{S}_k|}\delta_{i_k}} f\left(g^{-1}\left(\frac{1}{|\boldsymbol{S}_k|!}\sum_{p\in \boldsymbol{P}_{|\boldsymbol{S}_k|}} g\left(f^{-1}\left(\sum_{i_k=1}^{|\boldsymbol{S}_k|}\left(\delta_{i_k} f\left(g^{-1}\left((n\xi_{p(i_k)})g(\mu_{p(i_k)})\right)\right)\right)\right)\right)\right)\right) \geq$$
$$\frac{1}{\sum_{i_k=1}^{|\boldsymbol{S}_k|}\delta_{i_k}} f\left(f^{-1}\left(\left(\sum_{i_k=1}^{|\boldsymbol{S}_k|}\delta_{i_k}\right) f(\mu_{\mathrm{UB}})\right)\right) = f(\mu_{\mathrm{UB}})$$

Because $f^{-1}(x)$ is monotonically decreasing, we have

$$\mu_{\mathrm{LB}} = f^{-1}\left(f(\mu_{\mathrm{LB}})\right) \leq f^{-1}\left(\frac{1}{\sum_{i_k=1}^{|\boldsymbol{S}_k|}\delta_{i_k}} f\left(g^{-1}\left(\frac{1}{|\boldsymbol{S}_k|!}\sum_{p\in \boldsymbol{P}_{|\boldsymbol{S}_k|}} g\left(f^{-1}\left(\sum_{i_k=1}^{|\boldsymbol{S}_k|}\left(\delta_{i_k} f\left(g^{-1}\left((n\xi_{p(i_k)})g(\mu_{p(i_k)})\right)\right)\right)\right)\right)\right)\right)\right) \leq f^{-1}\left(f(\mu_{\mathrm{UB}})\right) = \mu_{\mathrm{UB}}$$

Since $g(x)$ is monotonically increasing, we can obtain

$$g(\mu_{\mathrm{LB}}) = \frac{1}{N}\sum_{k=1}^{N} g(\mu_{\mathrm{LB}}) \leq \frac{1}{N}\sum_{k=1}^{N} g\left(f^{-1}\left(\frac{1}{\sum_{i_k=1}^{|\boldsymbol{S}_k|}\delta_{i_k}} f\left(g^{-1}\left(\frac{1}{|\boldsymbol{S}_k|!}\sum_{p\in \boldsymbol{P}_{|\boldsymbol{S}_k|}} g\left(f^{-1}\left(\sum_{i_k=1}^{|\boldsymbol{S}_k|}\left(\delta_{i_k} f\left(g^{-1}\left((n\xi_{p(i_k)})g(\mu_{p(i_k)})\right)\right)\right)\right)\right)\right)\right)\right)\right) \leq \frac{1}{N}\sum_{k=1}^{N} g(\mu_{\mathrm{UB}}) = g(\mu_{\mathrm{UB}})$$

Finally, because $g^{-1}(x)$ is monotonically increasing, we have

$$\mu_{\mathrm{LB}} = g^{-1}\left(g(\mu_{\mathrm{LB}})\right) \leq g^{-1}\left(\frac{1}{N}\sum_{k=1}^{N} g\left(f^{-1}\left(\frac{1}{\sum_{i_k=1}^{|\boldsymbol{S}_k|}\delta_{i_k}} f\left(g^{-1}\left(\frac{1}{|\boldsymbol{S}_k|!}\sum_{p\in \boldsymbol{P}_{|\boldsymbol{S}_k|}} g\left(f^{-1}\left(\sum_{i_k=1}^{|\boldsymbol{S}_k|}\left(\delta_{i_k} f\left(g^{-1}\left((n\xi_{p(i_k)})g(\mu_{p(i_k)})\right)\right)\right)\right)\right)\right)\right)\right)\right)\right) \leq g^{-1}\left(g(\mu_{\mathrm{UB}})\right) = \mu_{\mathrm{UB}}$$

That is $\mu_{\mathrm{LB}} \leq \mu \leq \mu_{\mathrm{UB}}$. Similarly, we can prove $v_{\mathrm{LB}} \geq v \geq v_{\mathrm{UB}}$. Since $S(qROFAPPMM^{\Delta}(Q_1, Q_2, \ldots, Q_n)) = \mu^q - v^q$, $S(qROFAPPMM^{\Delta}(Q_{\mathrm{LB}}, Q_{\mathrm{LB}}, \ldots, Q_{\mathrm{LB}})) = \mu_{\mathrm{LB}}^q - v_{\mathrm{LB}}^q$, $S(qROFAPPMM^{\Delta}(Q_{\mathrm{UB}}, Q_{\mathrm{UB}}, \ldots, Q_{\mathrm{UB}})) = \mu_{\mathrm{UB}}^q - v_{\mathrm{UB}}^q$, and $0 \leq \mu_{\mathrm{LB}} \leq \mu \leq \mu_{\mathrm{UB}} \leq 1$ and $1 \geq v_{\mathrm{LB}} \geq v \geq v_{\mathrm{UB}} \geq 0$, we can obtain

$$qROFAPPMM^{\Delta}(Q_{\mathrm{LB}}, Q_{\mathrm{LB}}, \ldots, Q_{\mathrm{LB}}) \leq qROFAPPMM^{\Delta}(Q_1, Q_2, \ldots, Q_n) \leq qROFAPPMM^{\Delta}(Q_{\mathrm{UB}}, Q_{\mathrm{UB}}, \ldots, Q_{\mathrm{UB}})$$

Therefore, we can obtain $Q_{\mathrm{LB}} \leq qROFAPPMM^{\Delta}(Q_1, Q_2, \ldots, Q_n) \leq Q_{\mathrm{UB}}$. □
