# Supplementary figures and images for: Multi-criteria group decision making based on Archimedean power partitioned Muirhead mean operators of q-rung orthopair fuzzy numbers
Source: PLoS One. 2019 Sep 5;14(9):e0221759. doi: 10.1371/journal.pone.0221759 (PMC6728046; doi:10.1371/journal.pone.0221759)

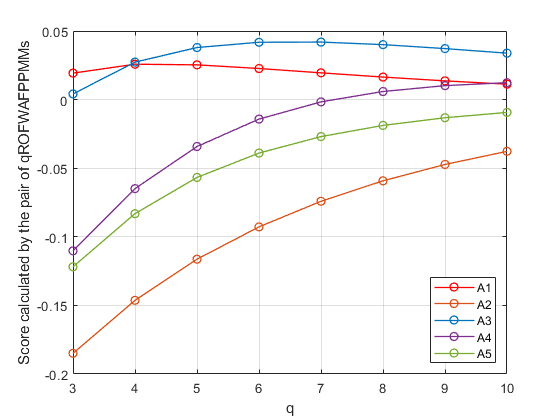

Supplement: S2 File — https://github.com/YuchuChingQin/AOsOfqROFNsForMCGDM. (ZIP) [file pone.0221759.s002.zip › AOsOfqROFNs/E2F.tif]

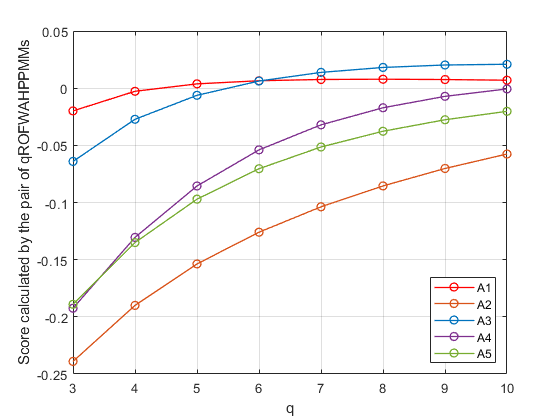

Supplement: S2 File — https://github.com/YuchuChingQin/AOsOfqROFNsForMCGDM. (ZIP) [file pone.0221759.s002.zip › AOsOfqROFNs/E2H.tif]

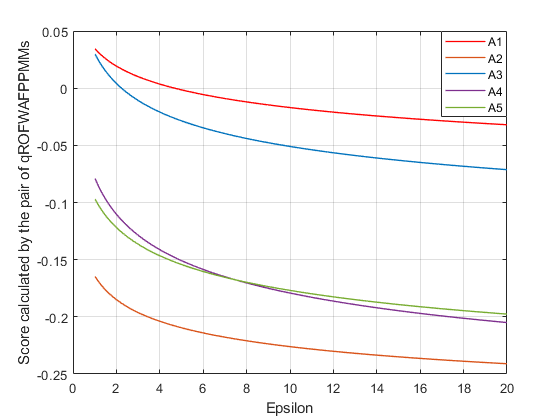

Supplement: S2 File — https://github.com/YuchuChingQin/AOsOfqROFNsForMCGDM. (ZIP) [file pone.0221759.s002.zip › AOsOfqROFNs/E3F.tif]

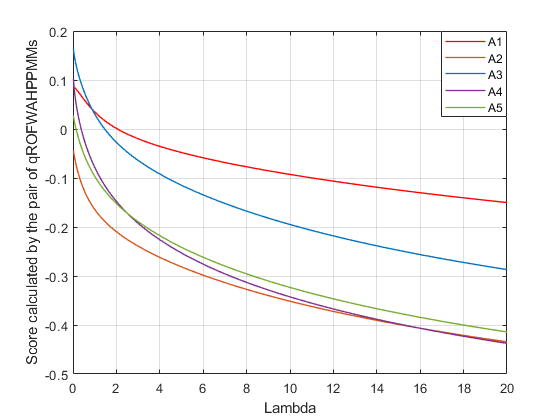

Supplement: S2 File — https://github.com/YuchuChingQin/AOsOfqROFNsForMCGDM. (ZIP) [file pone.0221759.s002.zip › AOsOfqROFNs/E3H.tif]

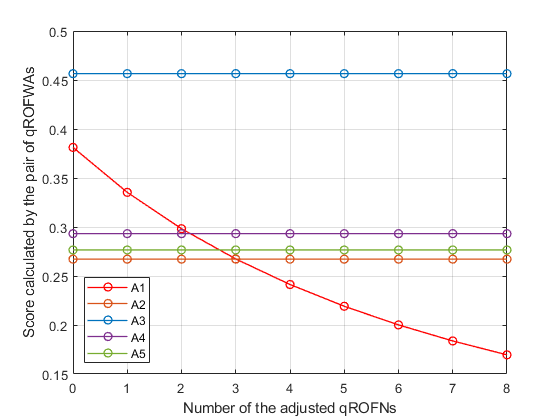

Supplement: S2 File — https://github.com/YuchuChingQin/AOsOfqROFNsForMCGDM. (ZIP) [file pone.0221759.s002.zip › AOsOfqROFNs/WA.tif]

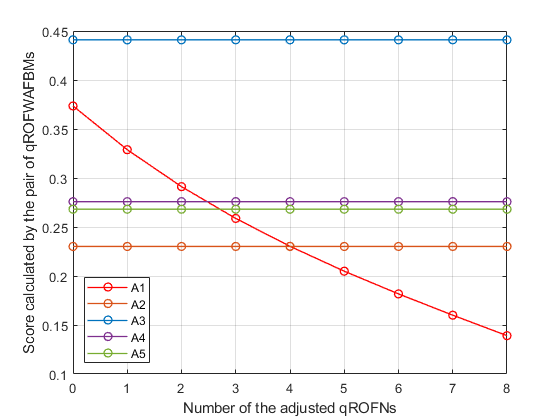

Supplement: S2 File — https://github.com/YuchuChingQin/AOsOfqROFNsForMCGDM. (ZIP) [file pone.0221759.s002.zip › AOsOfqROFNs/WAFBM.tif]

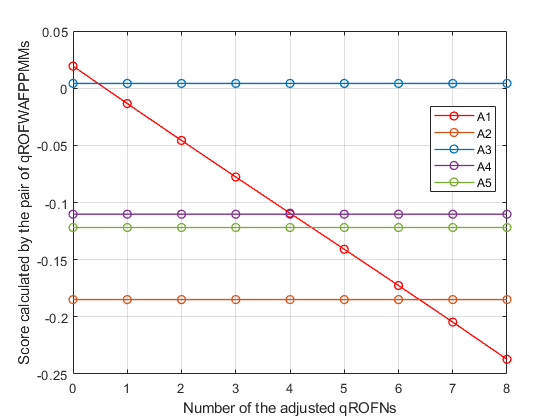

Supplement: S2 File — https://github.com/YuchuChingQin/AOsOfqROFNsForMCGDM. (ZIP) [file pone.0221759.s002.zip › AOsOfqROFNs/WAFPPMM.tif]

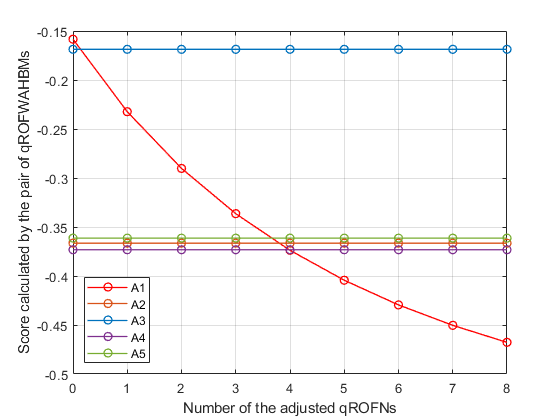

Supplement: S2 File — https://github.com/YuchuChingQin/AOsOfqROFNsForMCGDM. (ZIP) [file pone.0221759.s002.zip › AOsOfqROFNs/WAHBM.tif]

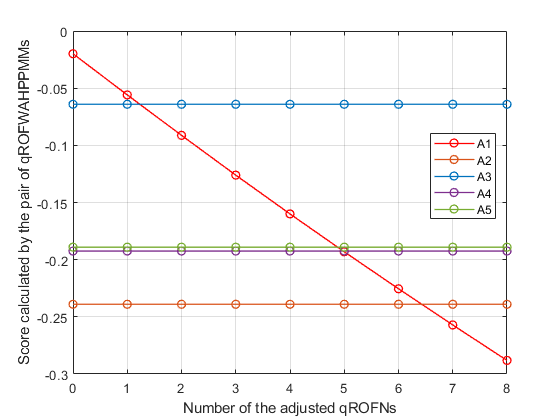

Supplement: S2 File — https://github.com/YuchuChingQin/AOsOfqROFNsForMCGDM. (ZIP) [file pone.0221759.s002.zip › AOsOfqROFNs/WAHPPMM.tif]

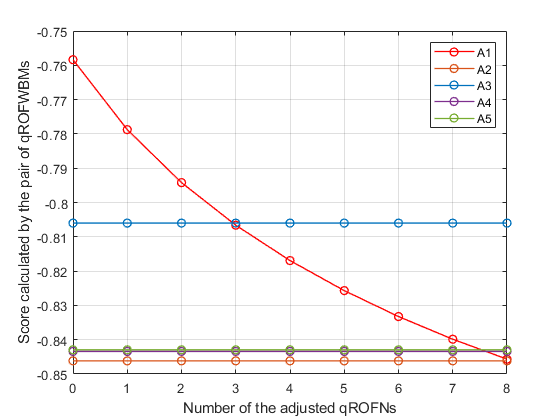

Supplement: S2 File — https://github.com/YuchuChingQin/AOsOfqROFNsForMCGDM. (ZIP) [file pone.0221759.s002.zip › AOsOfqROFNs/WBM.tif]

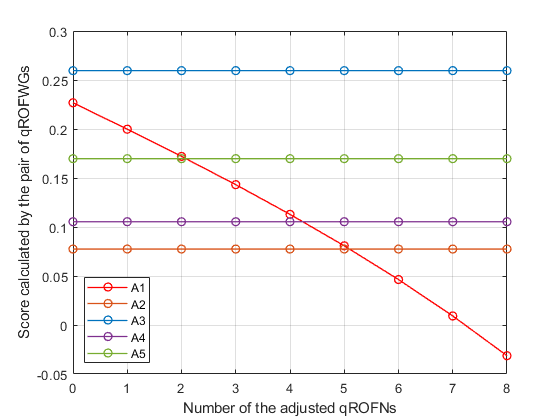

Supplement: S2 File — https://github.com/YuchuChingQin/AOsOfqROFNsForMCGDM. (ZIP) [file pone.0221759.s002.zip › AOsOfqROFNs/WG.tif]

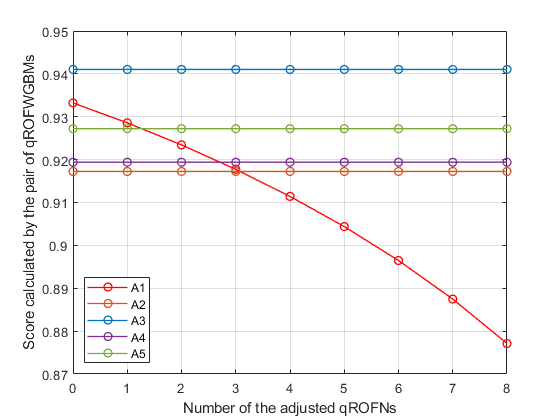

Supplement: S2 File — https://github.com/YuchuChingQin/AOsOfqROFNsForMCGDM. (ZIP) [file pone.0221759.s002.zip › AOsOfqROFNs/WGBM.tif]

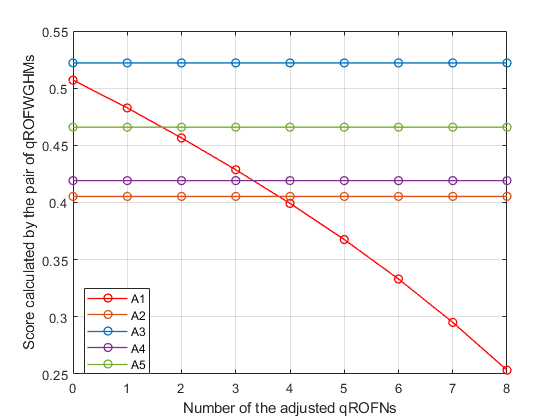

Supplement: S2 File — https://github.com/YuchuChingQin/AOsOfqROFNsForMCGDM. (ZIP) [file pone.0221759.s002.zip › AOsOfqROFNs/WGHM.tif]

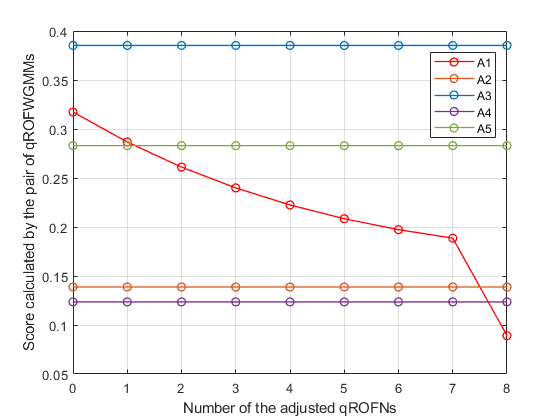

Supplement: S2 File — https://github.com/YuchuChingQin/AOsOfqROFNsForMCGDM. (ZIP) [file pone.0221759.s002.zip › AOsOfqROFNs/WGMM.tif]

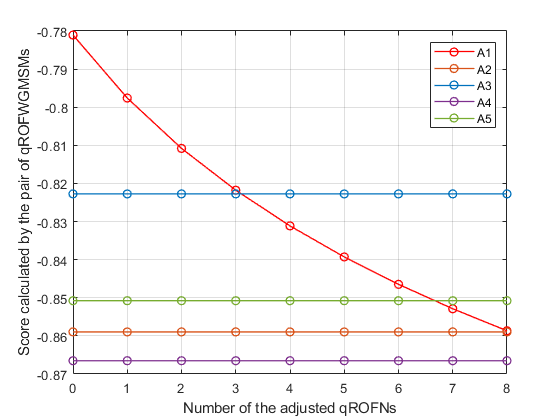

Supplement: S2 File — https://github.com/YuchuChingQin/AOsOfqROFNsForMCGDM. (ZIP) [file pone.0221759.s002.zip › AOsOfqROFNs/WGMSM.tif]

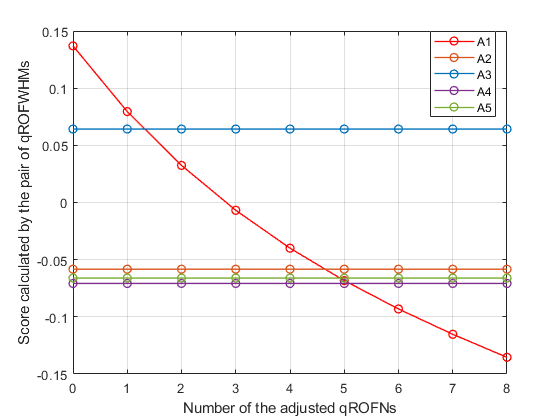

Supplement: S2 File — https://github.com/YuchuChingQin/AOsOfqROFNsForMCGDM. (ZIP) [file pone.0221759.s002.zip › AOsOfqROFNs/WHM.tif]

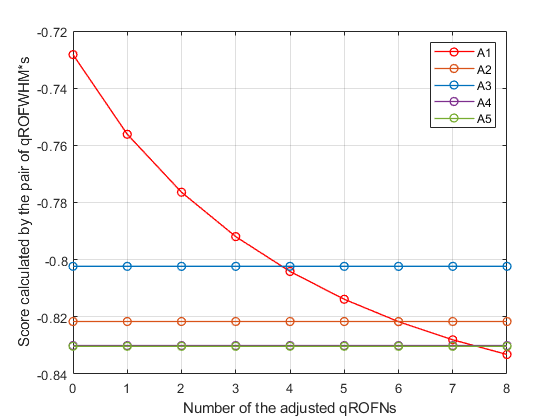

Supplement: S2 File — https://github.com/YuchuChingQin/AOsOfqROFNsForMCGDM. (ZIP) [file pone.0221759.s002.zip › AOsOfqROFNs/WHM2.tif]

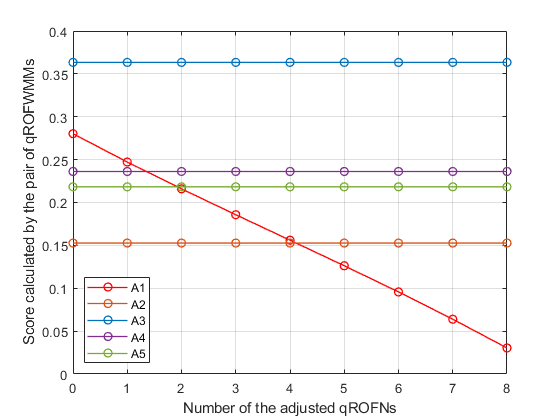

Supplement: S2 File — https://github.com/YuchuChingQin/AOsOfqROFNsForMCGDM. (ZIP) [file pone.0221759.s002.zip › AOsOfqROFNs/WMM.tif]

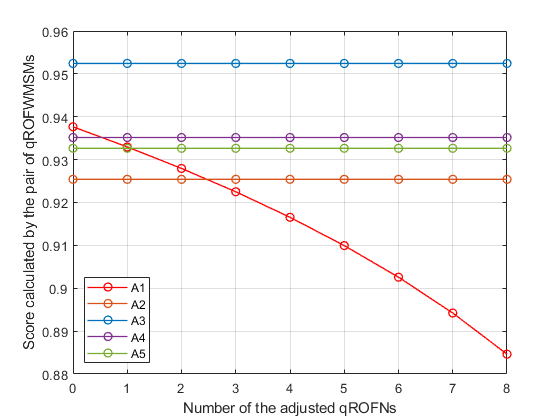

Supplement: S2 File — https://github.com/YuchuChingQin/AOsOfqROFNsForMCGDM. (ZIP) [file pone.0221759.s002.zip › AOsOfqROFNs/WMSM.tif]

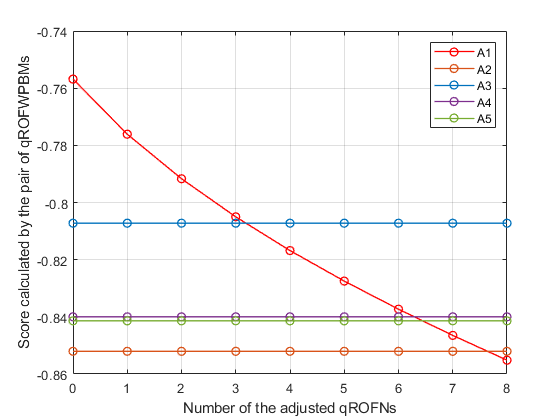

Supplement: S2 File — https://github.com/YuchuChingQin/AOsOfqROFNsForMCGDM. (ZIP) [file pone.0221759.s002.zip › AOsOfqROFNs/WPBM.tif]

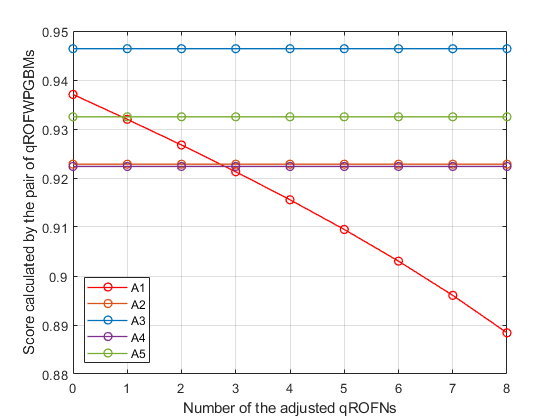

Supplement: S2 File — https://github.com/YuchuChingQin/AOsOfqROFNsForMCGDM. (ZIP) [file pone.0221759.s002.zip › AOsOfqROFNs/WPGBM.tif]

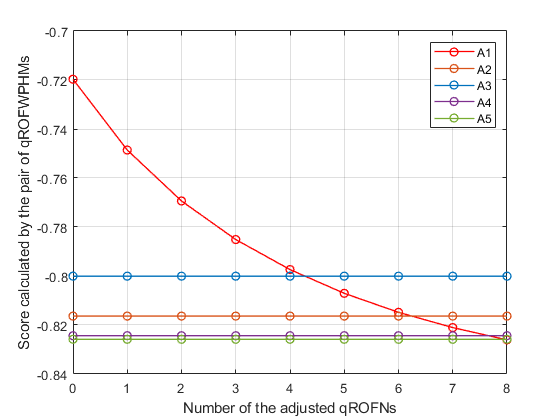

Supplement: S2 File — https://github.com/YuchuChingQin/AOsOfqROFNsForMCGDM. (ZIP) [file pone.0221759.s002.zip › AOsOfqROFNs/WPHM.tif]

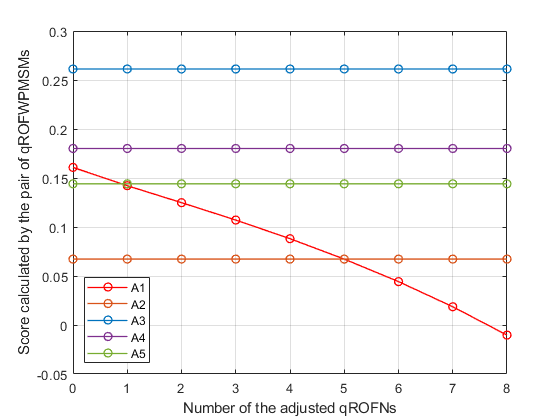

Supplement: S2 File — https://github.com/YuchuChingQin/AOsOfqROFNsForMCGDM. (ZIP) [file pone.0221759.s002.zip › AOsOfqROFNs/WPMSM.tif]

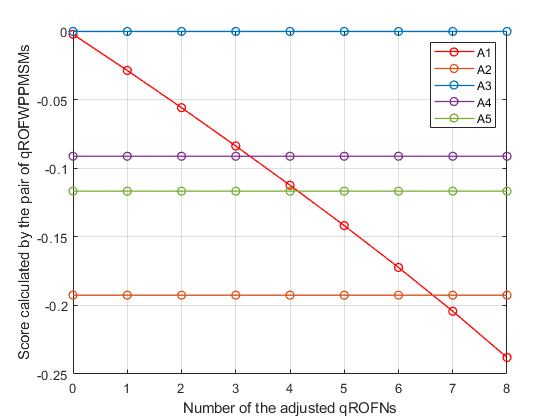

Supplement: S2 File — https://github.com/YuchuChingQin/AOsOfqROFNsForMCGDM. (ZIP) [file pone.0221759.s002.zip › AOsOfqROFNs/WPPMSM.tif]
